# Supplementary material for: Synthesis, Photochromic and Luminescent Properties of Ammonium Salts of Spiropyrans
Source: Molecules. 2022 Dec 2;27(23):8492. doi: 10.3390/molecules27238492 (PMC9736937; doi:10.3390/molecules27238492)
Supplement: Supplementary file 1 [file molecules-27-08492-s001.zip › molecules-2040246-Supplementary information revised.pdf]

Supplementary information

Synthesis, photochromic and luminescent properties of ammonium salts of spiropyrans

A.A. Khuzin,* D.I. Galimov, A.R. Tulyabaev, L.L. Khuzina

Institute of Petrochemistry and Catalysis Ufa Federal Research Center of the Russian Academy of Sciences, 141, Oktyabrya Prospect, 450075 Ufa, Russia

* E-mail address: artur.khuzin@gmail.com.

The ^1H and ^{13}C NMR spectra were run on a Bruker Avance-500 spectrometer at 500.17 and 125.78 MHz or on a Bruker Avance-400.13 spectrometer at 400.17 and 100.62 MHz, respectively. $CDCl_3$ was used as a solvent. High resolution mass spectrometry (HRMS) was performed on a MaXis impact, Bruker. Thin-layer chromatography was performed on silica gel plates (Sorbfil TLC, CTX-1VE) to monitor the reactions. Spots were made visible with UV light. Column chromatography was performed with silica gel (Sigma-Aldrich, high-purity grade, 60 Å/63–200 μm).

The spectrophotometric measurements were performed on a Cary 60 UV-Vis Spectrophotometer in 2- and 10-mm thick quartz cells. For photochemical measurements, the solutions were irradiated by an L8253 xenon lamp included in an LC-8 radiation unit (Hamamatsu) at a medium radiation power through colour glass filters, UFS-1 and SZS-9.

The photoluminescence (PL) and excitation spectra, luminescence lifetimes of test compounds were registered using Horiba Jobin Yvon Fluorolog-3 spectrofluorometer (model FL-3-22) equipped with doublegrating monochromators, dual lamp housing containing a 450 W Xenon lamp, laser-diode NanoLED, photomultiplier tube detector (Hamamatsu R928 PMT), and InGaAs photodiode (DSS-IGA020 L) for IR measurements. For measurements of luminescence lifetimes were used a pulsed (frequency is 1 MHz) laser-diode NanoLED-370 (λ_{exc} = 369 nm), NanoLED-455 (λ_{exc} = 453 nm) and NanoLED-590 (λ_{exc} = 592 nm). The PL and excitation spectra were corrected in all cases for source intensity (lamp and grating) and emission spectral response (detector and grating) by standard instrument correction provided in the instrument software FluorEssence 3.5.

The PL quantum yield (ϕ_{PL}) of **22** in merocyanine form in THF was calculated *via* Eq. 1. We were used the solutions (10^{-4} M) of anthracene in ethanol as the reference with the known and repeatedly measured quantum yield ϕ_{PL} = 0.291.

$$\frac{\phi_x}{\phi_{st}} = \frac{\varepsilon_{st} n_x^2 D_x}{\varepsilon_x n_{st}^2 D_{st}} \qquad (I)$$

where ϕ_{st} and ϕ_x are the PL quantum yields of the sample and the standard; ε_x and ε_{st} are absorbances at the excitation wavelength for the sample and the standard; n_x and n_{st} are the refractive indexes of the solvents (1.3611 and 1.4050 for ethanol and THF, respectively); D_x and D_{st} are the areas under the PL spectra of the sample and the standard.

Phenylhydrazine, 1,3-dibromopropane, 1,4-dibromobutane, 1,5-dibromopentane, 1,6-dibromohexane, 1,8-dibromooctane, triethylamine, 2M dimethylamine in H_2O solution were purchased from Aldrich and used as received.

Synthesis of compound 1: A mixture of 60 g (1 eq) of phenylhydrazine and 60 ml (1 eq) of 3-methyl-2-butanone was refluxed in glacial acetic acid for 2.5 h. After cooling, the mixture was neutralized, the organic phase was separated and distilled. Yield: 93% of theoretical. ^{1}H NMR (400 MHz, $CDCl_3$): δ = 1.28 (s, 6H, $2CH_3$), 2.27 (s, 3H, $1CH_3$), 7.18 (t, J = 7.3 Hz,1H), 7.27 (m, 2H), 7.53 (d, J = 7.5 Hz, 1H) ppm. ^{13}C NMR (100 MHz, $CDCl_3$): δ = 15.36, 23.05, 53.57, 119.81, 121.29, 125.12, 127.57, 145.58, 153.51, 188.05 ppm.

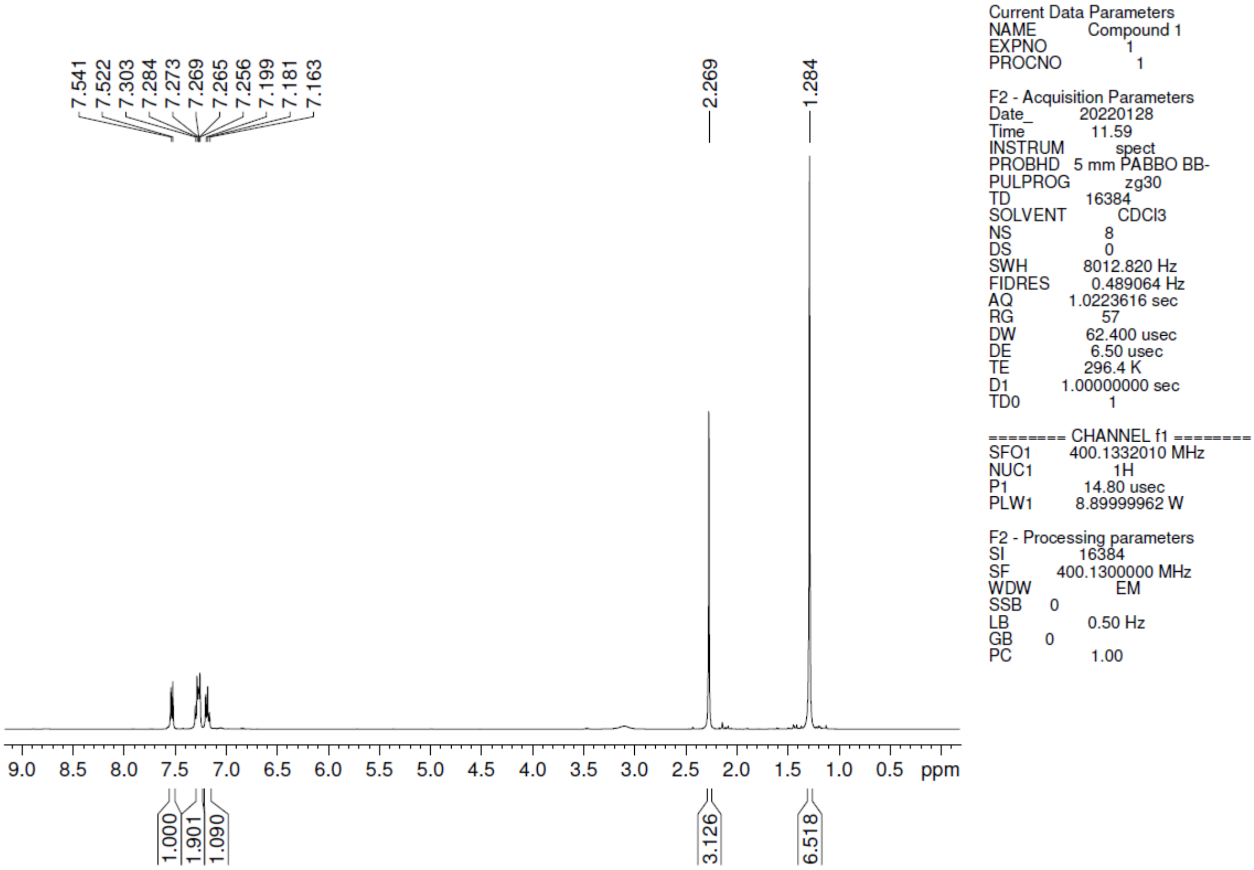

Figure S1. ^{1}H NMR spectra of compound **1** (400 MHz, $CDCl_3$).

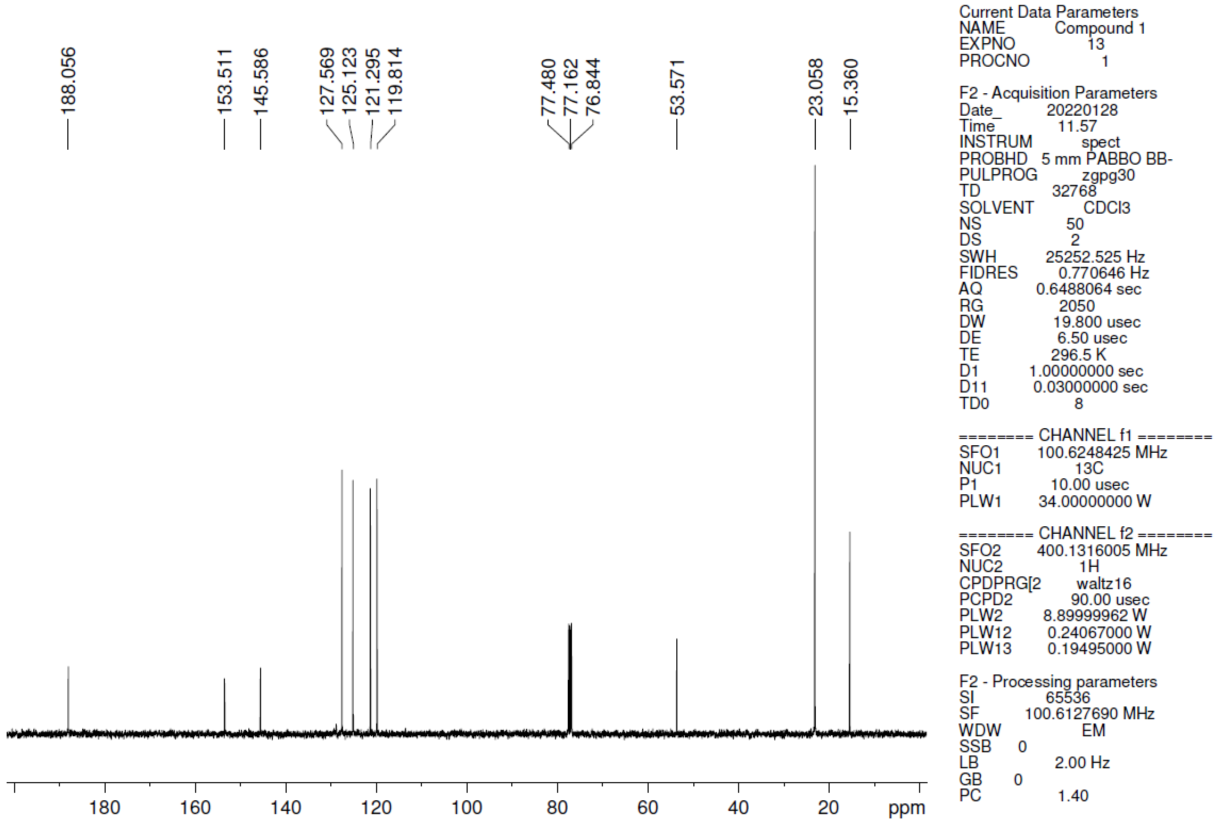

Figure S2. ^{13}C NMR spectra of compound **1** (100 MHz, $CDCl_3$).

General procedure for the synthesis of compounds 2-6: 2,3,3-Trimethyl-3H-indol **1** (1.0 eq) and dibromoalkane (3.0 eq) was refluxed in 95% ethanole for 8 h. Sequential purification via flash chromatography (acetone; dichloromethane:ethanole 10:1) afforded compounds **2-6** (25-30%) as a light yellow solid.

Compound 2: ^{1}H NMR (400 MHz, $CDCl_3$): δ = 1.32 (s, 6H), 1.88 (br. s, 2H), 2.01 (d, J = 3.0 Hz, 2H), 2.99 (br. s, 2H), 4.19 (br. s, 2H), 7.24 (m, 4H), 7.40 (d, J = 6.5 Hz, 1H) ppm. ^{13}C NMR (100 MHz, $CDCl_3$): δ = 16.40, 19.85, 23.25, 24.47, 46.02, 53.58, 114.57, 122.70, 128.94, 129.71, 141.19, 141.26, 195.23 ppm.

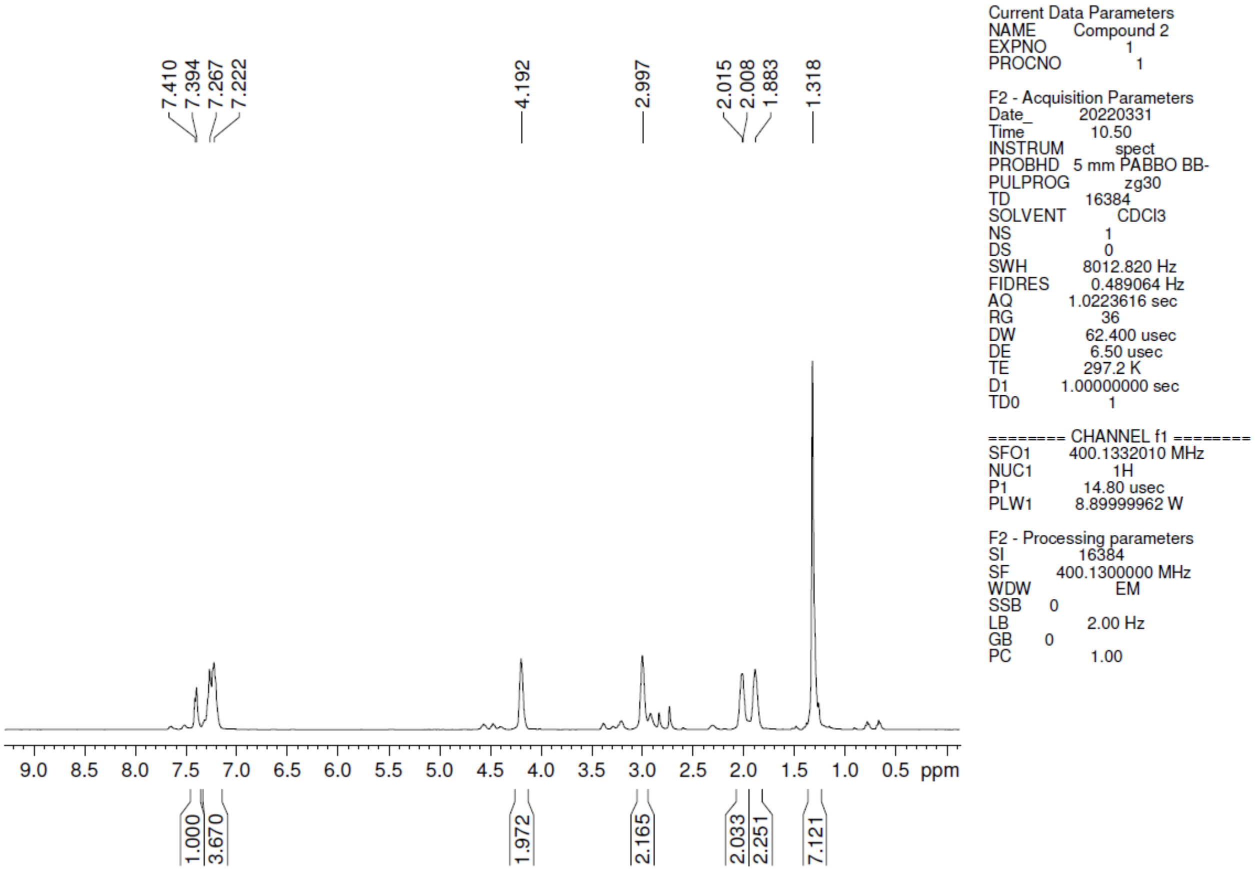

Figure S3. ^{1}H NMR spectra of compound **2** (400 MHz, $CDCl_3$).

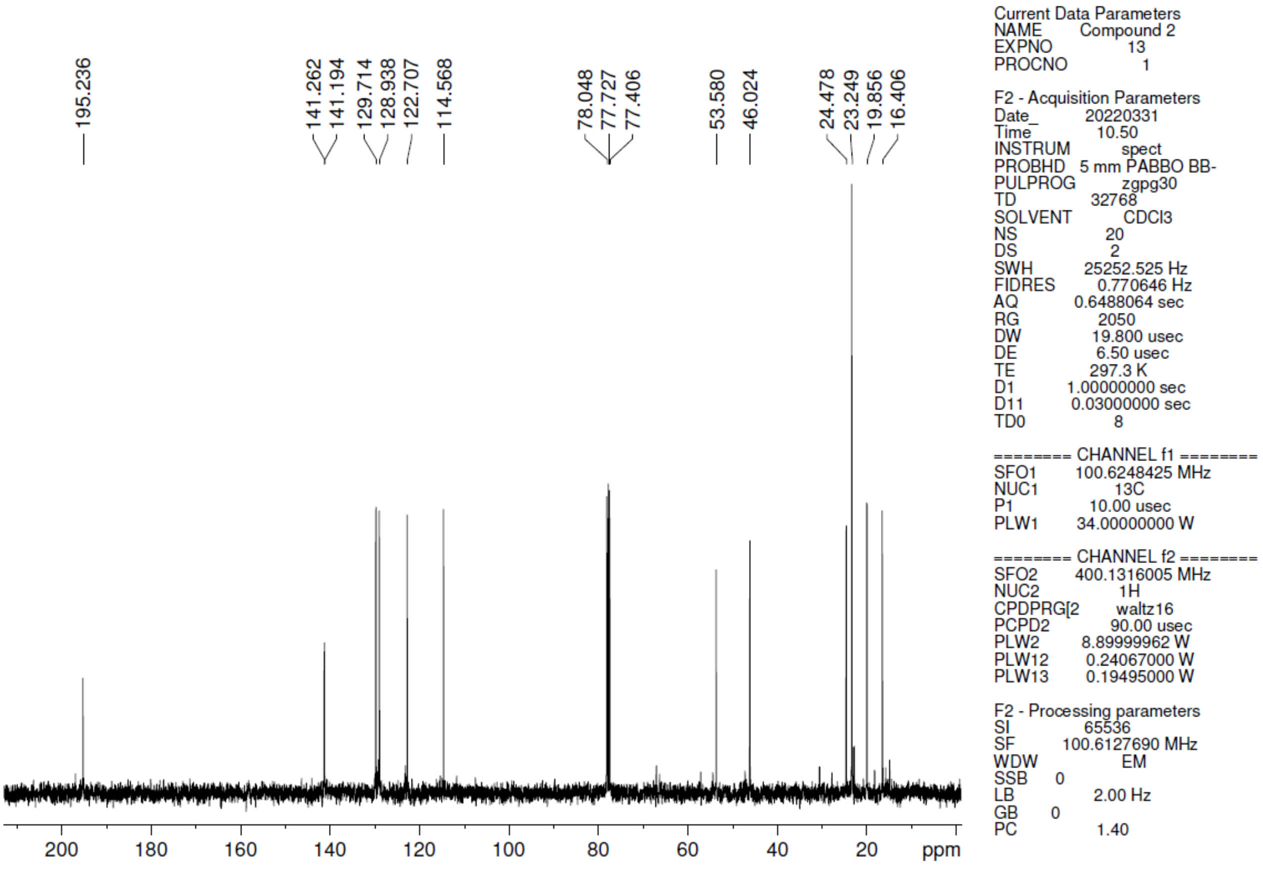

Figure S4. ^{13}C NMR spectra of compound **2** (100 MHz, $CDCl_3$).

Compound 3: ^{1}H NMR (400 MHz, $CDCl_3$): δ = 1.42 (s, 6H), 1.93 (br. s, 4H), 2.93 (s, 3H), 3.32 (t, *J* = 5.3 Hz, 2H), 4.59 (t, *J* = 6.6 Hz, 2H), 7.39 (m, 3H), 7.70 (m, 3H) ppm. ^{13}C NMR (100 MHz, $CDCl_3$): δ = 16.12, 22.92, 26.37, 29.12, 33.18, 48.19, 54.52, 115.52, 123.25, 129.43, 129.98, 140.71, 141.39, 196.18 ppm.

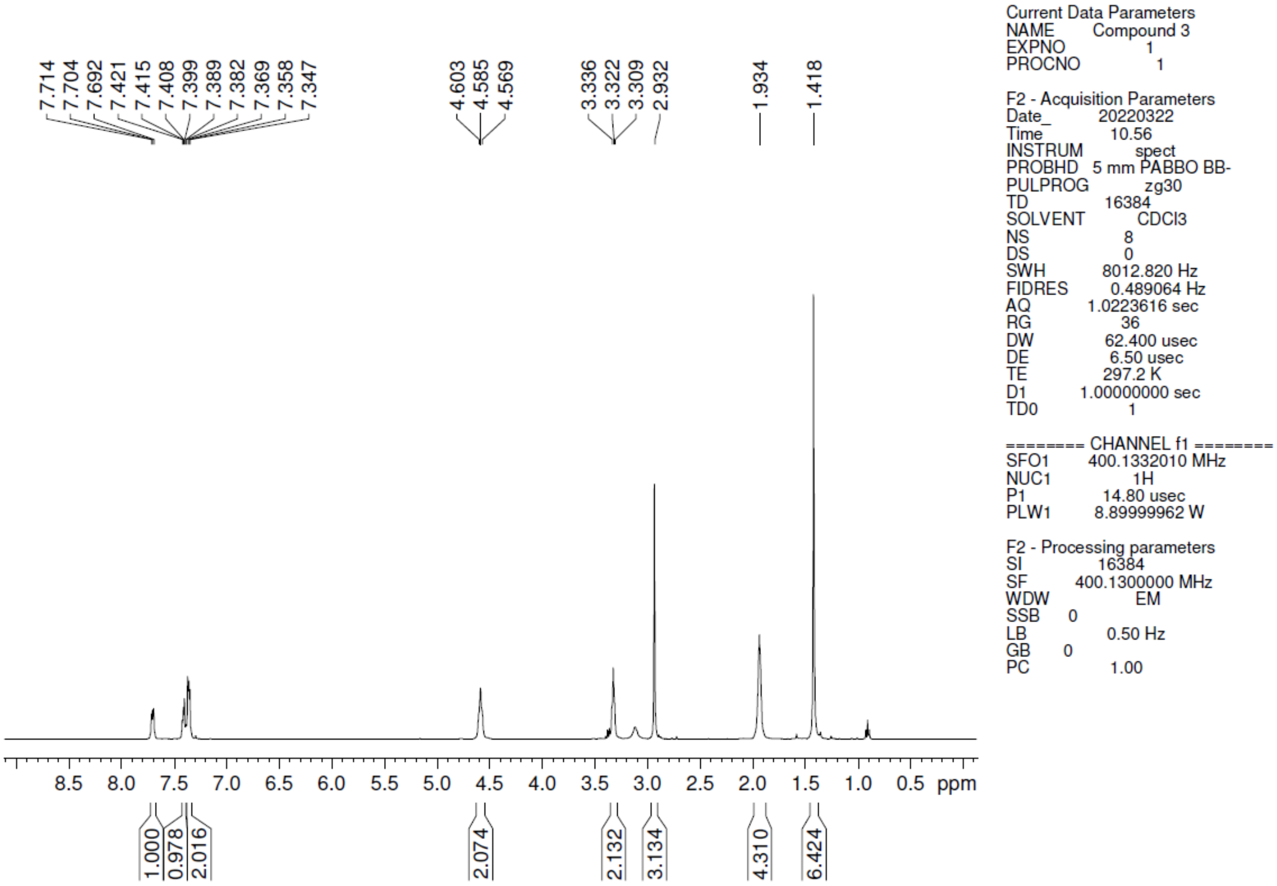

Figure S5. ^{1}H NMR spectra of compound **3** (400 MHz, $CDCl_3$).

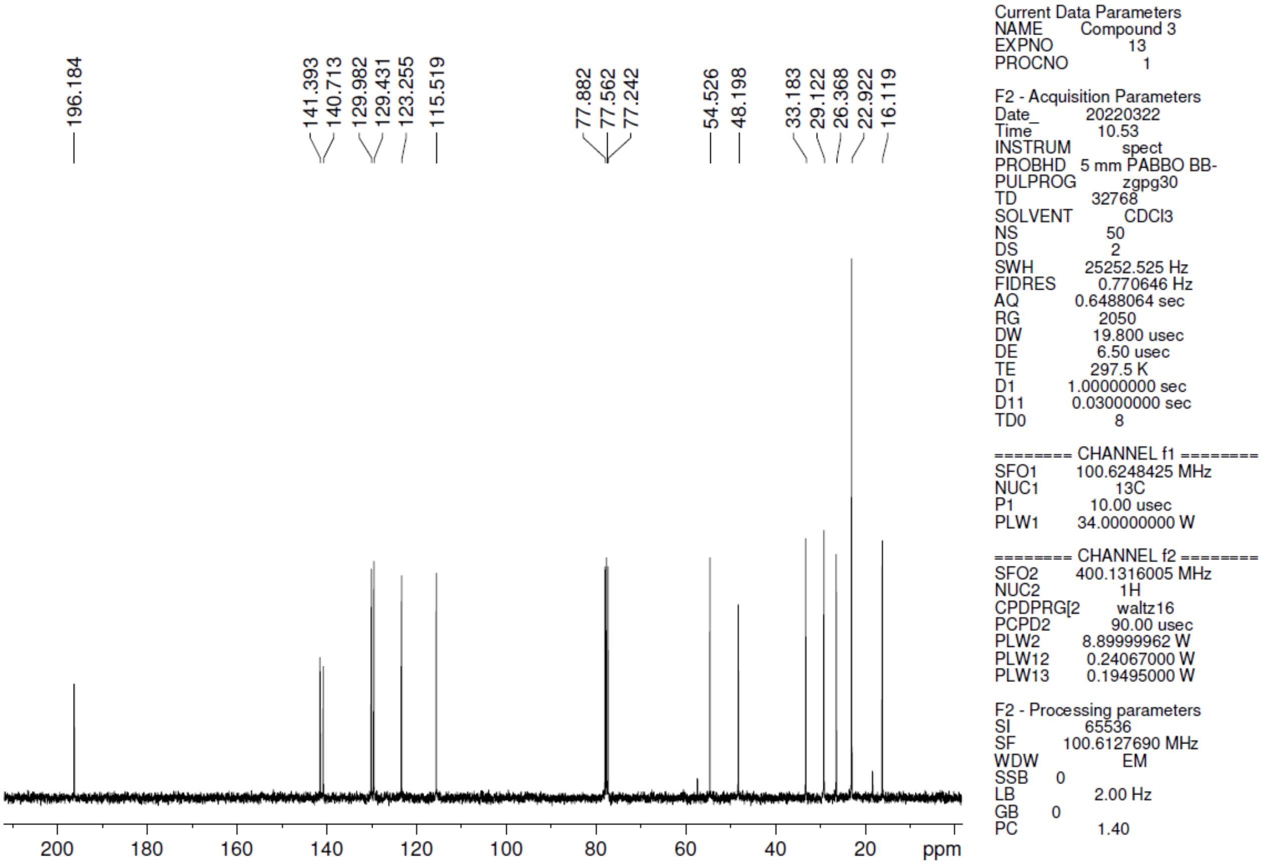

Figure S6. ^{13}C NMR spectra of compound **3** (100 MHz, $CDCl_3$).

Compound 4: ^{1}H NMR (400 MHz, $CDCl_3$): δ = 1.64 (s, 6H), 1.93 (dt, J = 15.6, 7.8 Hz, 2H), 2.02 (dt, J = 15.7, 7.9 Hz, 2H), 3.18 (s, 3H), 3.42 (t, J = 6.4 Hz, 2H) 4.81 (t, J = 7.6 Hz, 2H), 7.58 (s, 3H), 7.77 (d, J = 4.7 Hz, 1H) ppm. ^{13}C NMR (100 MHz, $CDCl_3$): δ = 16.52, 23.15, 25.14, 27.13, 31.64, 33.49, 49.39, 54.65, 115.50, 123.29, 129.57, 130.09, 140.97, 141.64, 196.31 ppm.

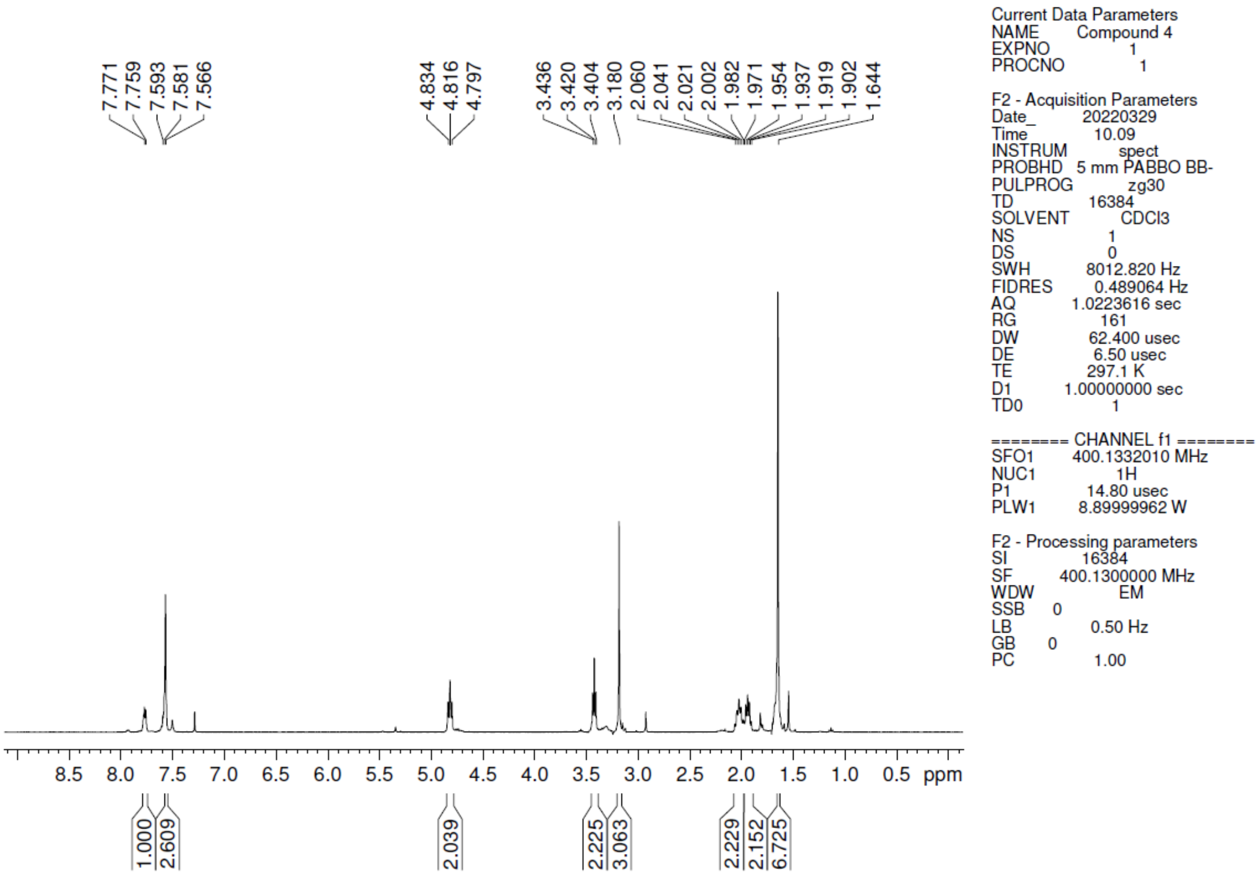

Figure S7. ^{1}H NMR spectra of compound **4** (400 MHz, $CDCl_3$).

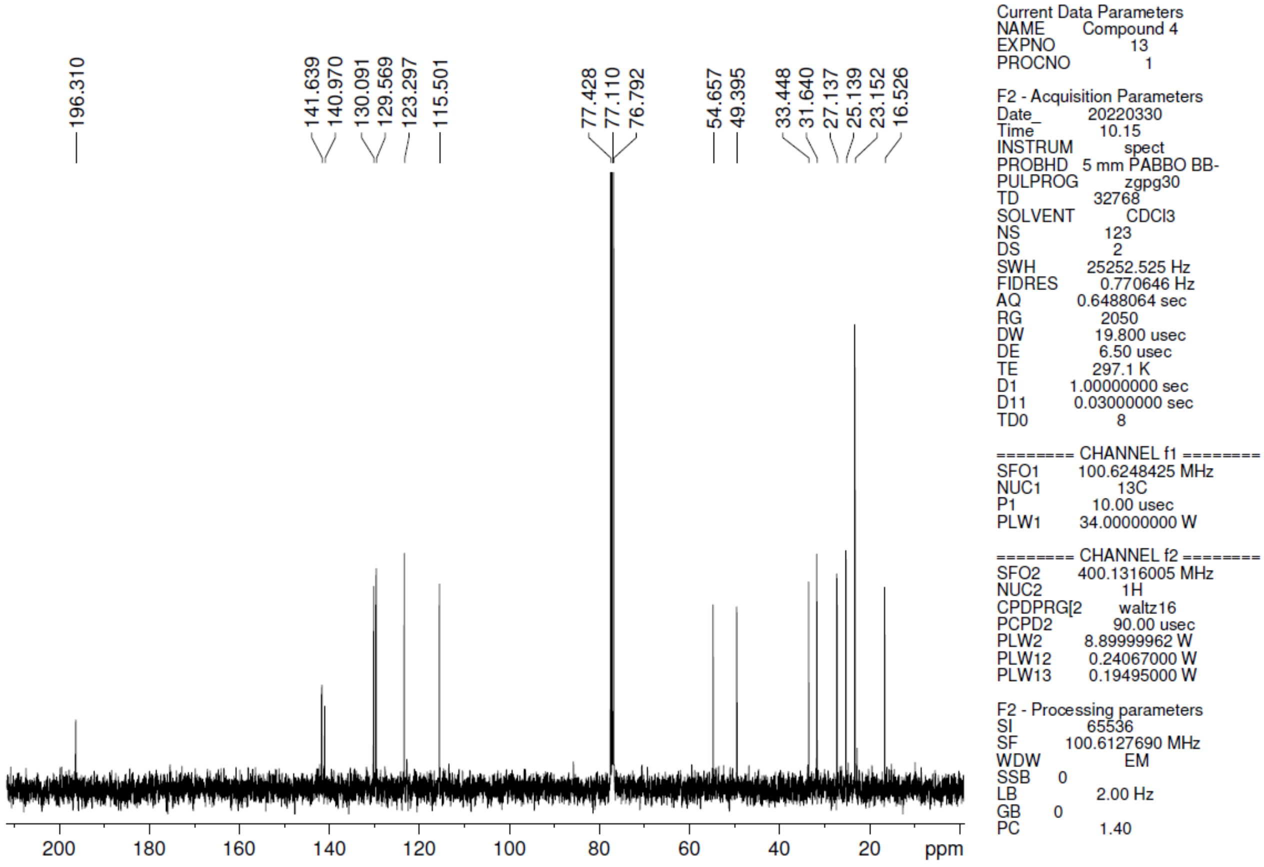

Figure S8. 13C NMR spectra of compound **4** (100 MHz, $CDCl_3$).

Compound 5: ^{1}H NMR (400 MHz, $CDCl_3$): δ = 1.37 (m, 4H), 1.48 (s, 6 H), 1.70 (m, 2H), 1.82 (m, 2H), 2.96 (s, 3H), 3.24 (t, *J* = 6.6 Hz, 2H) 4.56 (t, *J* = 7.7 Hz, 2H), 7.42 (m, 2H), 7.46 (m, 1H), 7.62 (m, 1H) ppm. ^{13}C NMR (100 MHz, $CDCl_3$): δ = 16.13, 22.97, 25.74, 27.49, 27.84, 32.11, 33.83, 49.14, 54.55, 115.34, 123.34, 129.43, 130.02, 140.74, 141.51, 195.94 ppm.

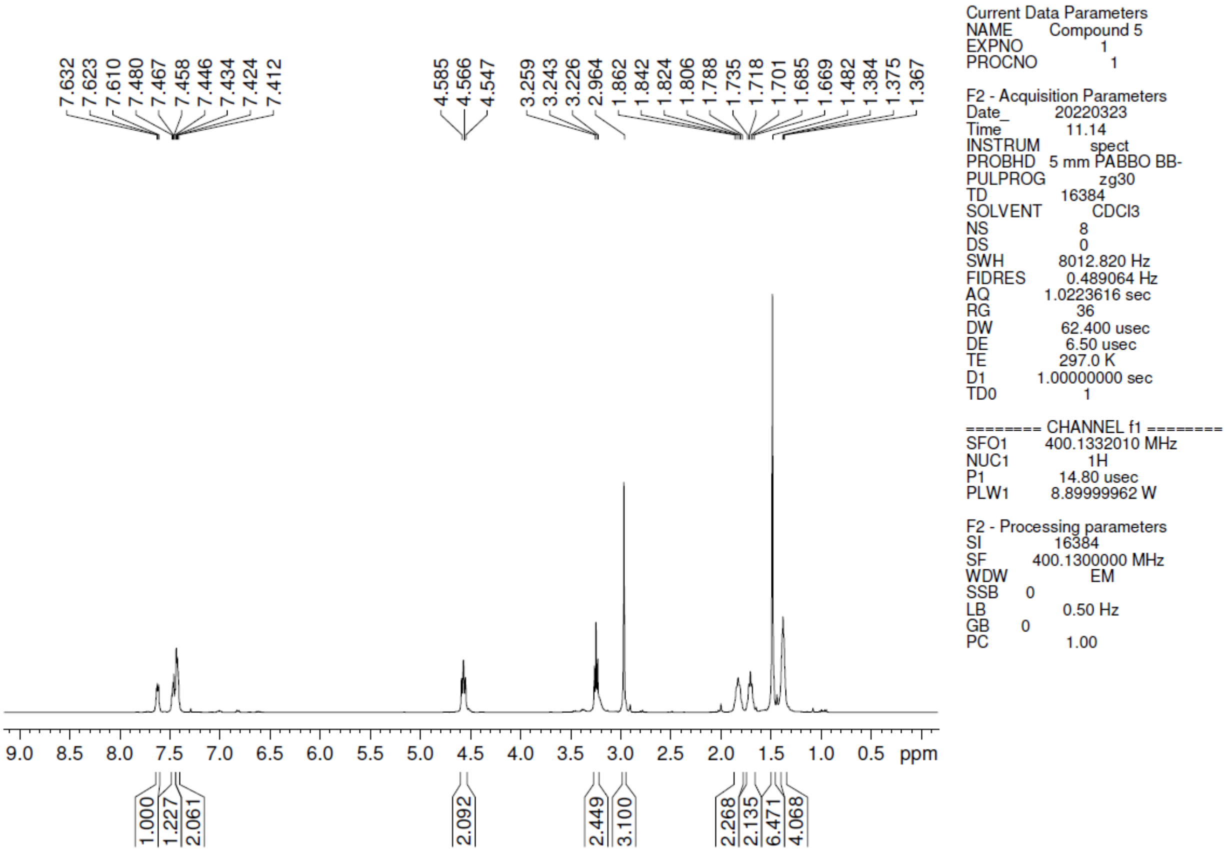

Figure S9. ^{1}H NMR spectra of compound **5** (400 MHz, $CDCl_3$).

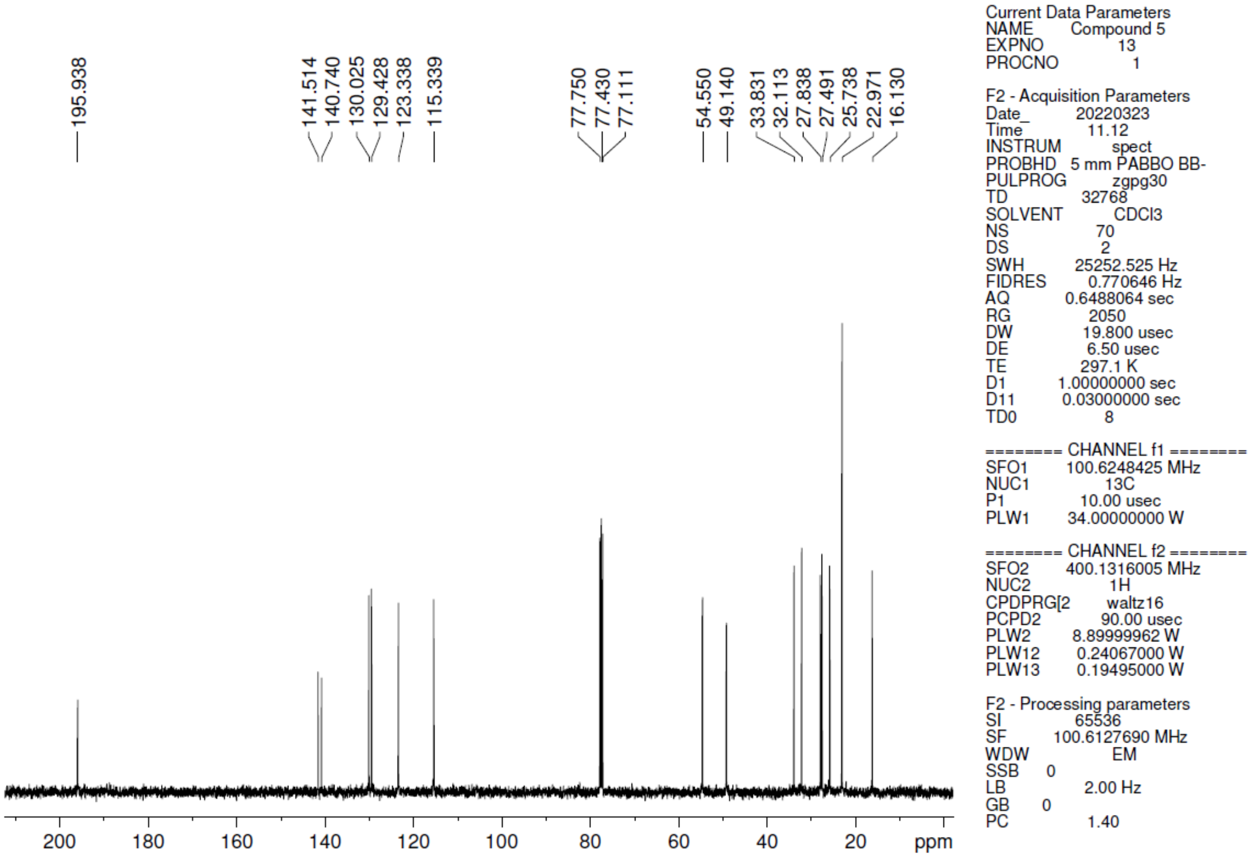

Figure S10. 13C NMR spectra of compound **5** (100 MHz, $CDCl_3$).

Compound 6: ^{1}H NMR (400 MHz, $CDCl_3$): δ = 1.34 (m, 9H), 1.58 (s, 6 H), 1.76 (m, 2H), 1.89 (m, 2H), 2.96 (s, 3H), 3.08 (s, 3H), 3.34 (m, 3H), 4.67 (t, J = 7.4 Hz, 2H), 7.52 (m, 3H), 7.65 (m, 1H) ppm. ^{13}C NMR (100 MHz, $CDCl_3$): δ = 16.27, 23.05, 26.57, 27.79, 27.98, 28.30, 28.86, 32.49, 34.05, 49.47, 54.61, 115.30, 123.35, 129.46, 130.05, 140.89, 141.65, 195.95 ppm.

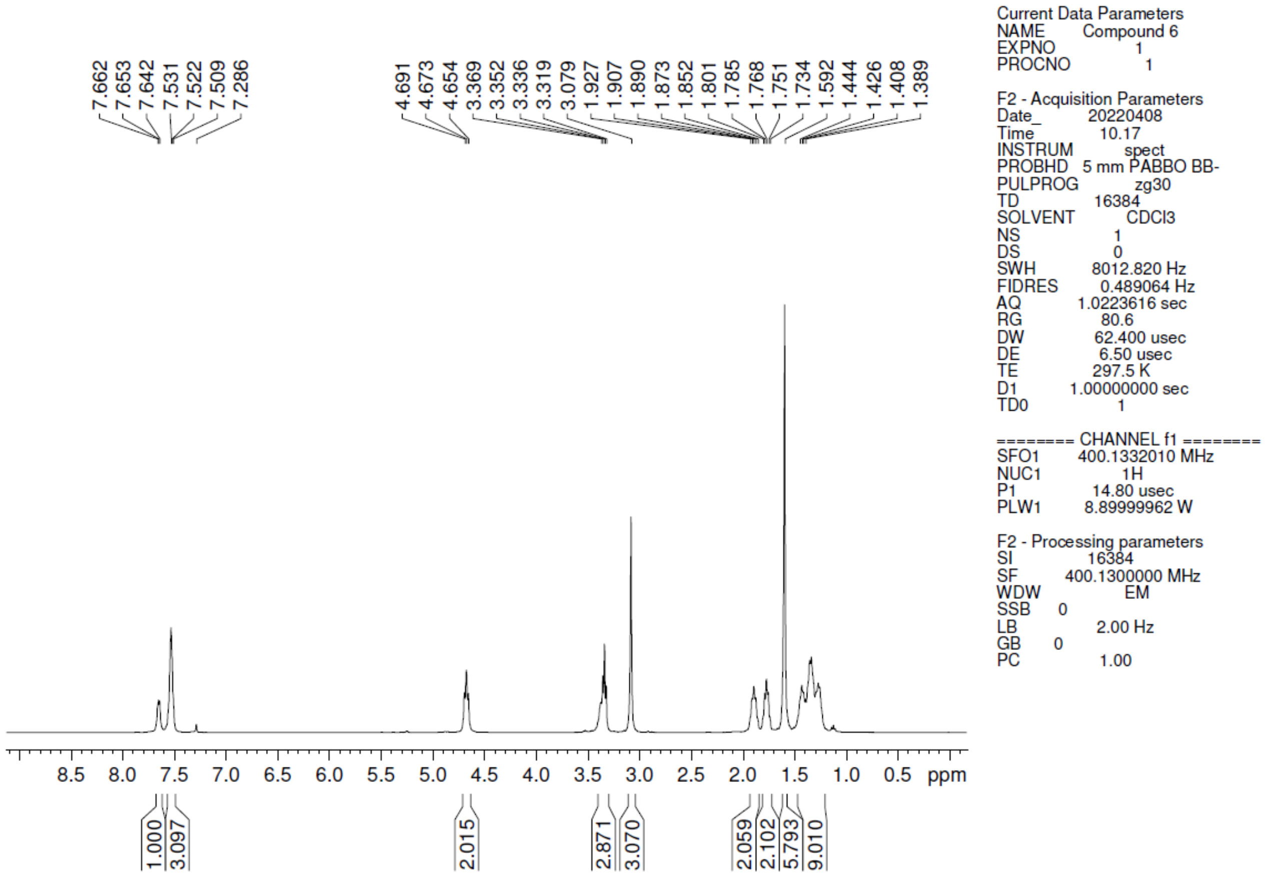

Figure S11. ^{1}H NMR spectra of compound **6** (400 MHz, $CDCl_3$).

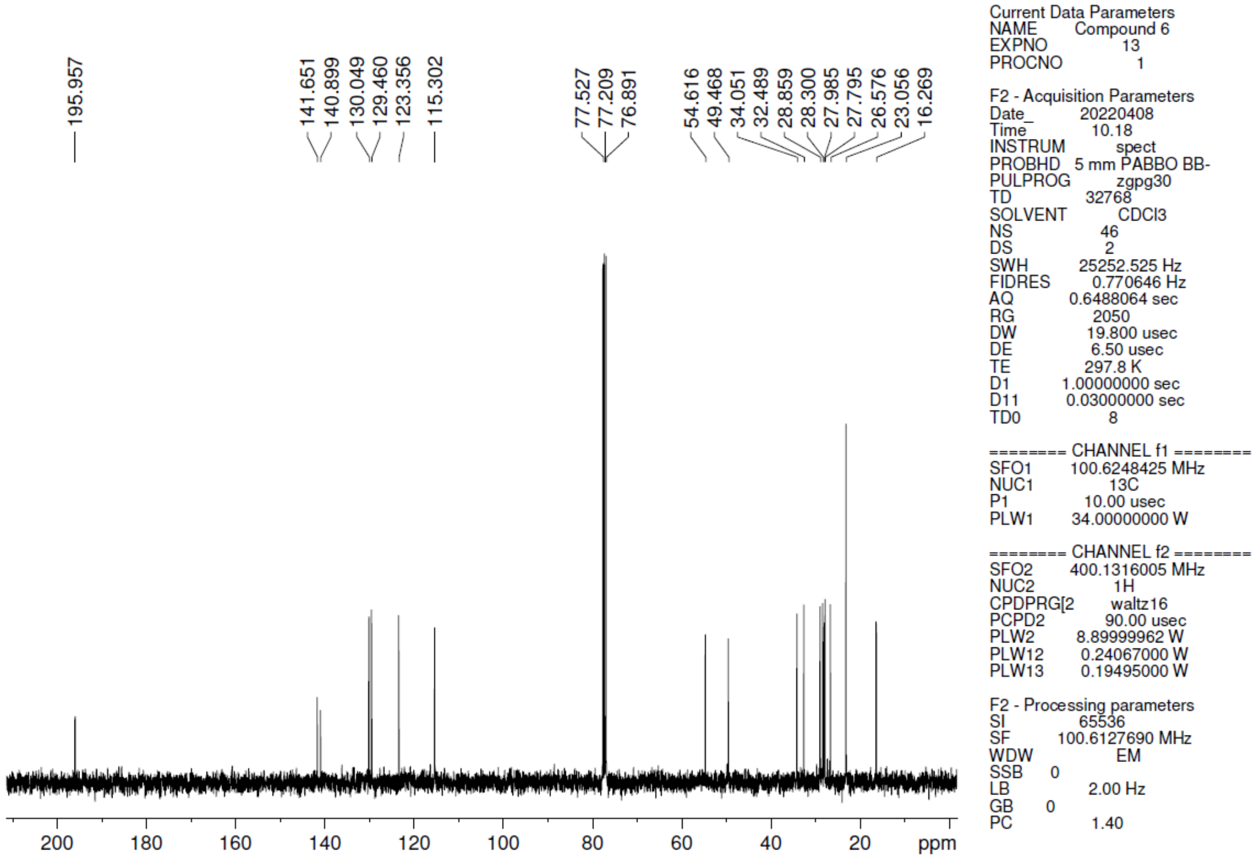

Figure S12. ^{1}H NMR spectra of compound **6** (100 MHz, $CDCl_3$).

General procedure for the synthesis of compounds 7-11: A solution of compounds **2-6** (1.0 eq), 2-hydroxy-5-nitrobenzaldehyde (1.0 eq) and Et_3N (1.1 eq) in EtOH were refluxed for 8 h. The white precipitate was removed. The crude product was purified by flash chromatography on silica gel with petroleum ether/EtOAc (10:1) as eluent to afford compouds **7-11** as light yellow solid (40-45%).

Compound 7: ^{1}H NMR (400 MHz, $CDCl_3$): δ = 1.22 (s, 3H), 1.32 (s, 3 H), 2.13 (m, 1H), 2.29 (m, 1H), 3.32 (m, 1H), 3.45 (m, 3H), 5.91 (d, *J* = 10.4 Hz, 1H), 6.68 (d, *J* = 7.8 Hz, 1H), 6.78 (d, *J* = 9.5 Hz, 1H), 6.93 (t, *J* = 7.4 Hz, 1H), 6.97 (d, *J* = 10.4 Hz, 1H), 7.13 (d, *J* = 7.1 Hz, 1H), 7.23 (t, *J* = 7.6 Hz, 1H), 8.04 (s, 1H), 8.06 (d, *J* = 2.5 Hz, 1H) ppm. ^{13}C NMR (100 MHz, $CDCl_3$): δ = 19.90, 25.97, 31.03, 31.77, 42.01, 52.65, 106.68, 106.86, 115.56, 118.52, 119.84, 121.74, 121.87, 122.80, 125.96, 127.84, 128.49, 136.03, 141.07, 146.83, 159.43 ppm.

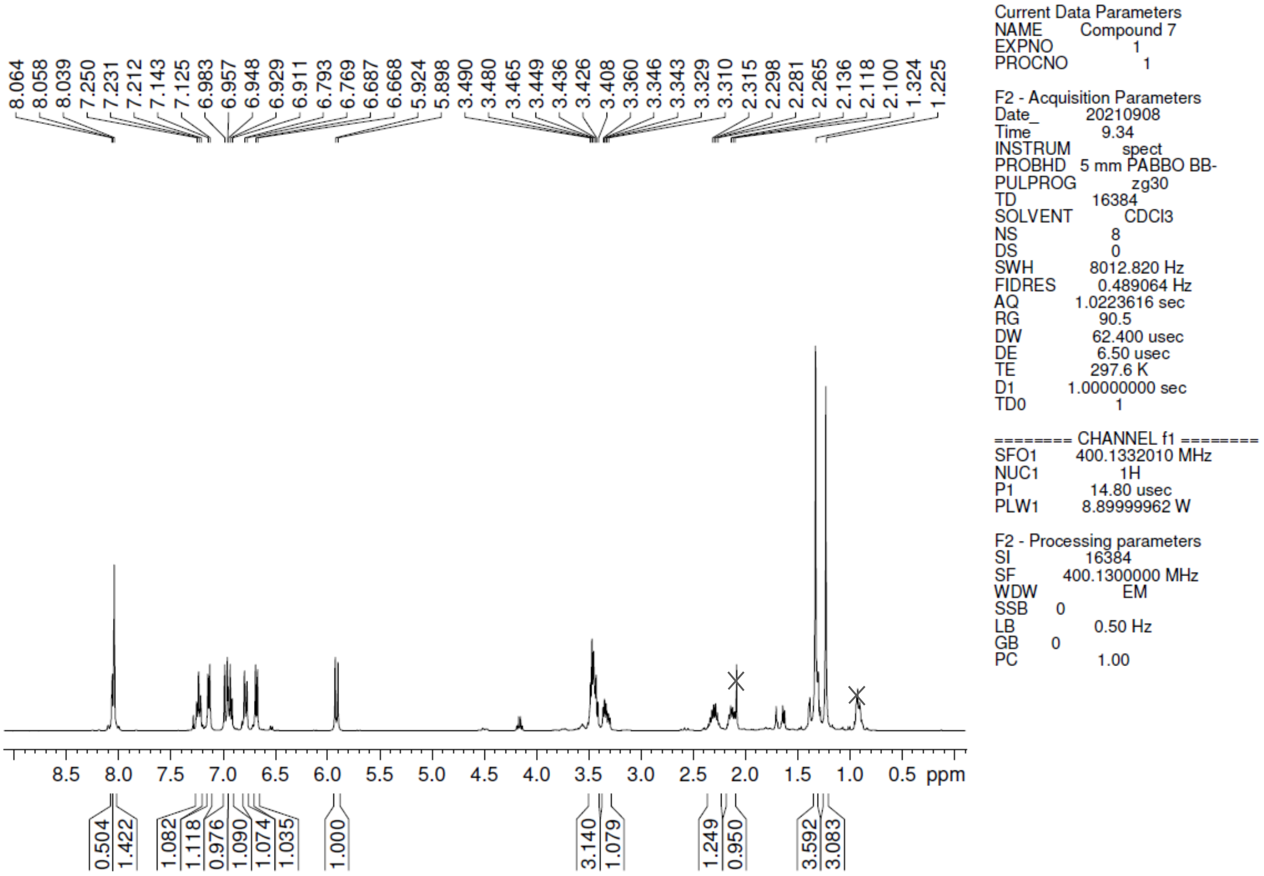

Figure S13. ^{1}H NMR spectra of compound **7** (400 MHz, $CDCl_3$).

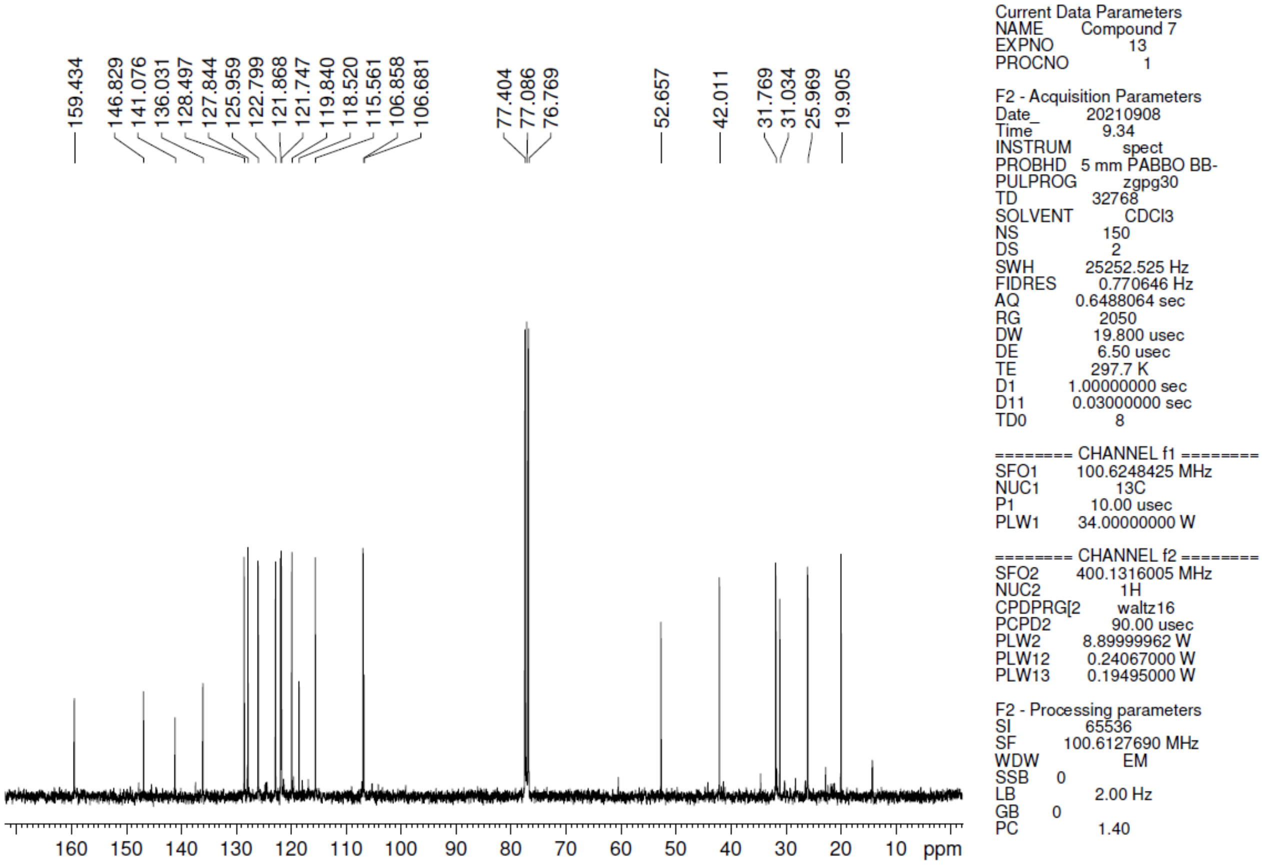

Figure S14. 13C NMR spectra of compound **7** (100 MHz, $CDCl_3$).

Compound 8: ^{1}H NMR (400 MHz, $CDCl_3$): δ = 1.22 (s, 3H), 1.32 (s, 3 H), 1.88 (m, 4H), 3.22 (m, 2H), 3.41 (m, 2H), 5.90 (d, J = 10.4 Hz, 1H), 6.61 (d, J = 7.8 Hz, 1H), 6.77 (d, J = 9.3 Hz, 1H), 6.91 (t, J = 7.4 Hz, 1H), 6.95 (d, J = 10.4 Hz, 1H), 7.12 (d, J = 7.1 Hz, 1H), 7.22 (t, J = 7.6 Hz, 1H), 8.03 (s, 1H), 8.05 (d, J = 2.5 Hz, 1H) ppm. ^{13}C NMR (100 MHz, $CDCl_3$): δ = 19.90, 26.02, 27.68, 30.47, 33.33, 42.99, 52.65, 106.70, 115.57, 118.45, 119.61, 121.79, 121.86, 122.77, 125.95, 127.82, 128.29, 135.97, 140.99, 146.97, 159.57 ppm.

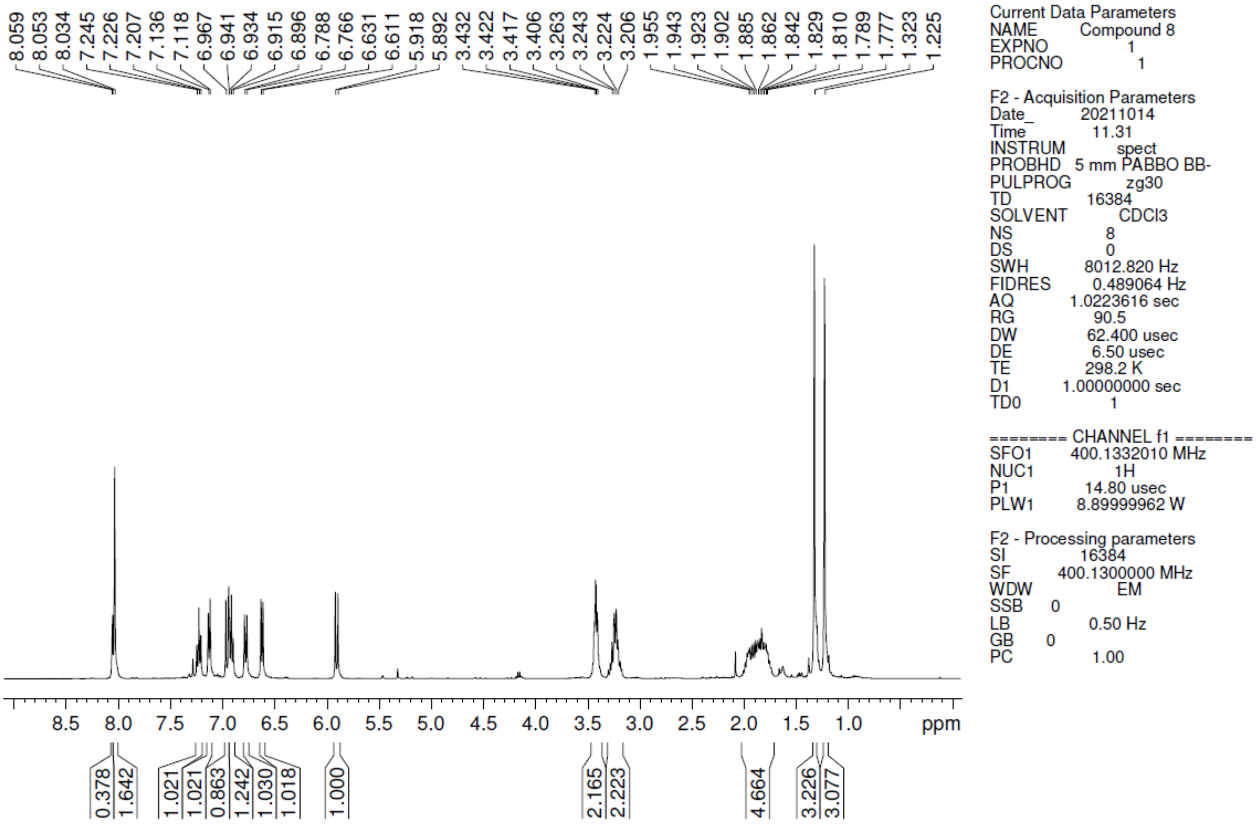

Figure S15. ^{1}H NMR spectra of compound **8** (400 MHz, $CDCl_3$).

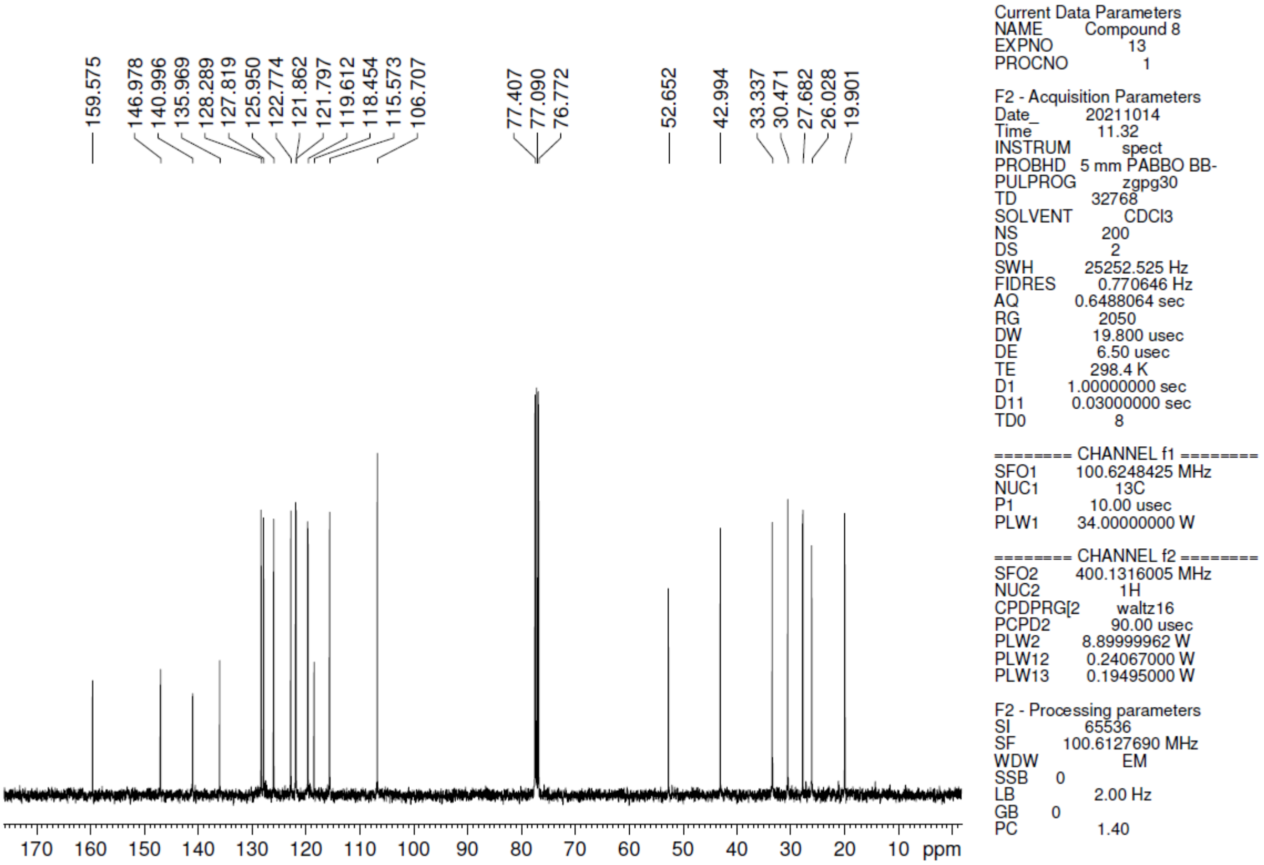

Figure S16. 13C NMR spectra of compound **8** (100 MHz, $CDCl_3$).

Compound 9: ^{1}H NMR (400 MHz, $CDCl_3$): δ = 1.22 (s, 3H), 1.31 (s, 3 H), 1.51 (m, 2H), 1.59 (m, 1H), 1.72 (m, 1H), 1.88 (m, 2H), 3.20 (m, 2H), 3.41 (t, *J* = 6.5 Hz, 2H), 5.91 (d, *J* = 10.3 Hz, 1H), 6.60 (d, *J* = 7.7 Hz, 1H), 6.77 (d, *J* = 9.2 Hz, 1H), 6.90 (t, *J* = 7.4 Hz, 1H), 6.95 (d, *J* = 10.4 Hz, 1H), 7.12 (d, *J* = 7.1 Hz, 1H), 7.22 (t, *J* = 7.5 Hz, 1H), 8.04 (d, *J* = 7.3 Hz, 2H) ppm. ^{13}C NMR (100 MHz, $CDCl_3$): δ = 19.88, 25.87, 26.03, 28.12, 32.41, 33.70, 43.55, 52.65, 106.66, 106.75, 115.54, 118.48, 119.45, 121.76, 121.99, 122.76, 125.92, 127.77, 128.21, 135.97, 140.92, 147.04, 159.64 ppm.

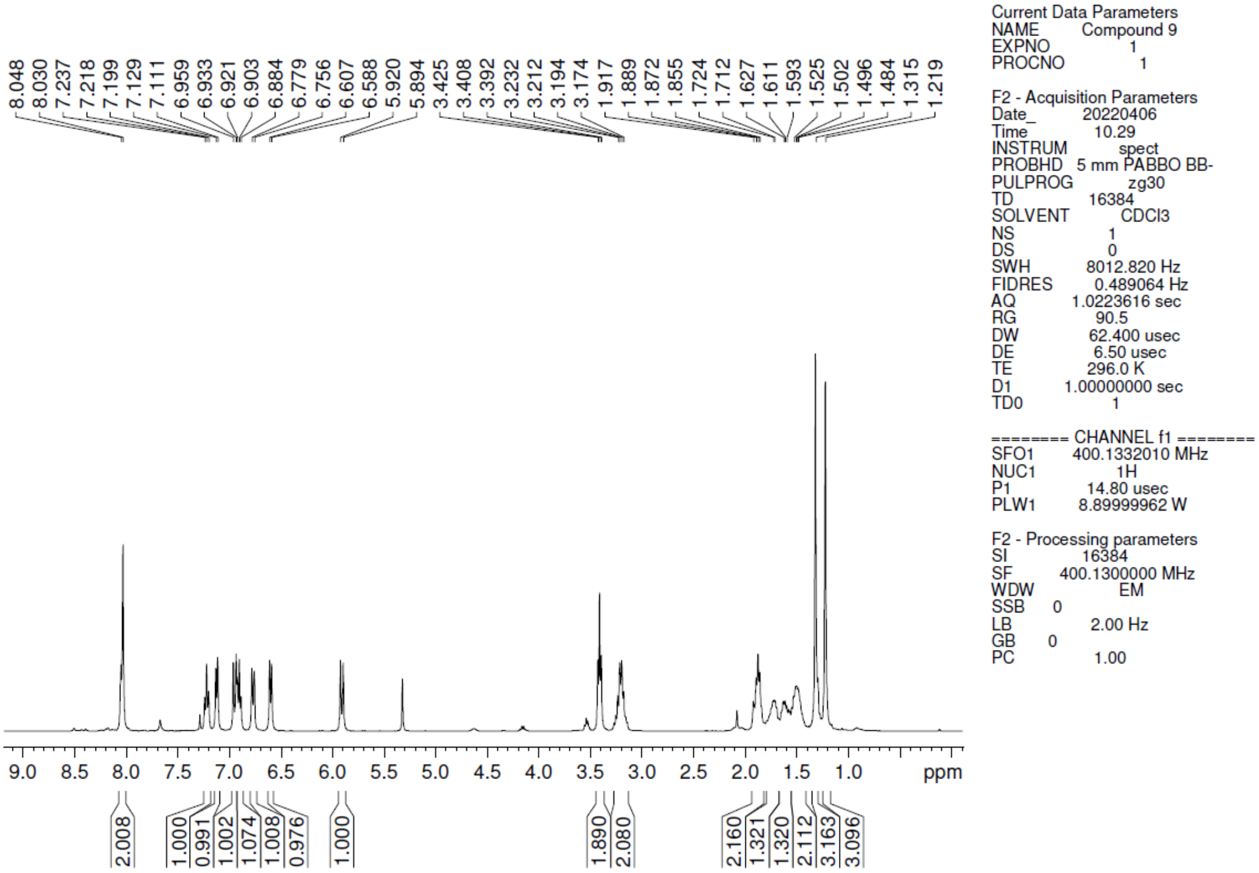

Figure S17. ^{1}H NMR spectra of compound **9** (400 MHz, $CDCl_3$).

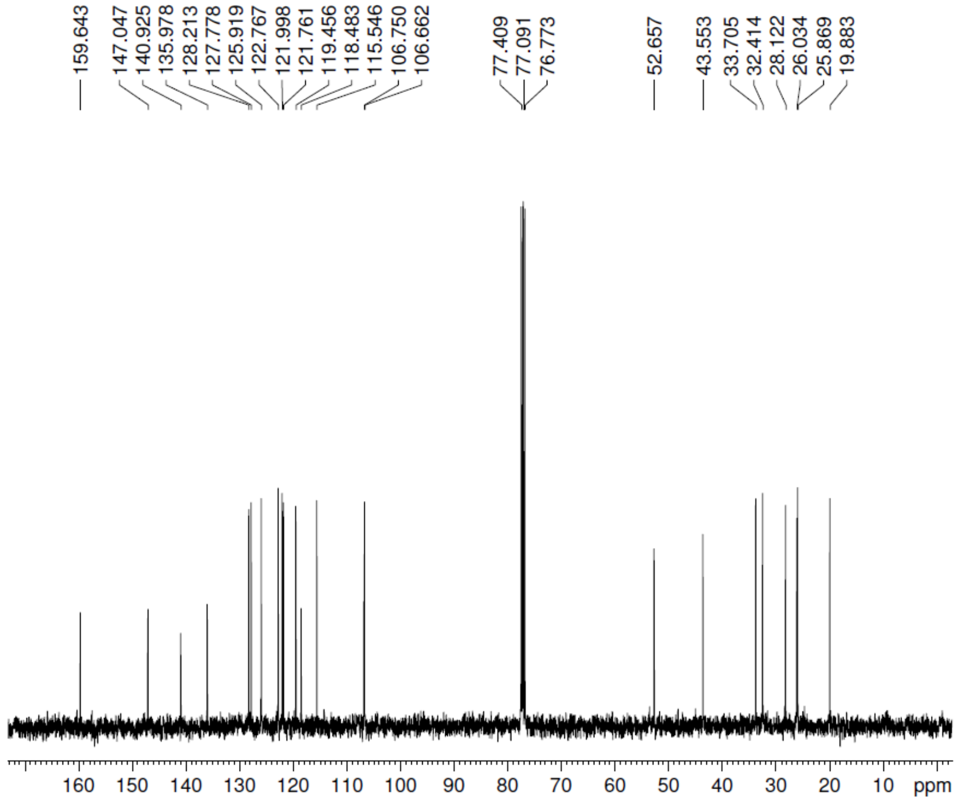

Figure S18. 13C NMR spectra of compound **9** (100 MHz, $CDCl_3$).

Compound 10: ^{1}H NMR (400 MHz, $CDCl_3$): δ = 1.23 (s, 3H), 1.30 (s, 3 H), 1.37 (m, 2H), 1.45 (m, 3H), 1.58 (m, 2H), 1.71 (m, 2H), 1.85 (m, 2H), 3.19 (m, 2H), 3.40 (t, *J* = 6.7 Hz, 2H), 5.89 (d, *J* = 10.4 Hz, 1H), 6.59 (d, *J* = 7.7 Hz, 1H), 6.76 (d, *J* = 8.6 Hz, 1H), 6.89 (t, *J* = 7.4 Hz, 1H), 6.94 (d, *J* = 10.4 Hz, 1H), 7.11 (d, *J* = 7.2 Hz, 1H), 7.21 (t, *J* = 7.5 Hz, 1H), 8.03 (s, 1H), 8.05 (d, *J* = 2.5 Hz, 1H) ppm. ^{13}C NMR (100 MHz, $CDCl_3$): δ = 19.86, 26.05, 26.47, 27.93, 28.80, 32.67, 33.81, 43.60, 52.64, 106.65, 106.74, 115.55, 118.49, 119.37, 121.72, 121.99, 122.74, 125.90, 127.76, 128.16, 135.95, 140.90, 147.09, 159.67 ppm.

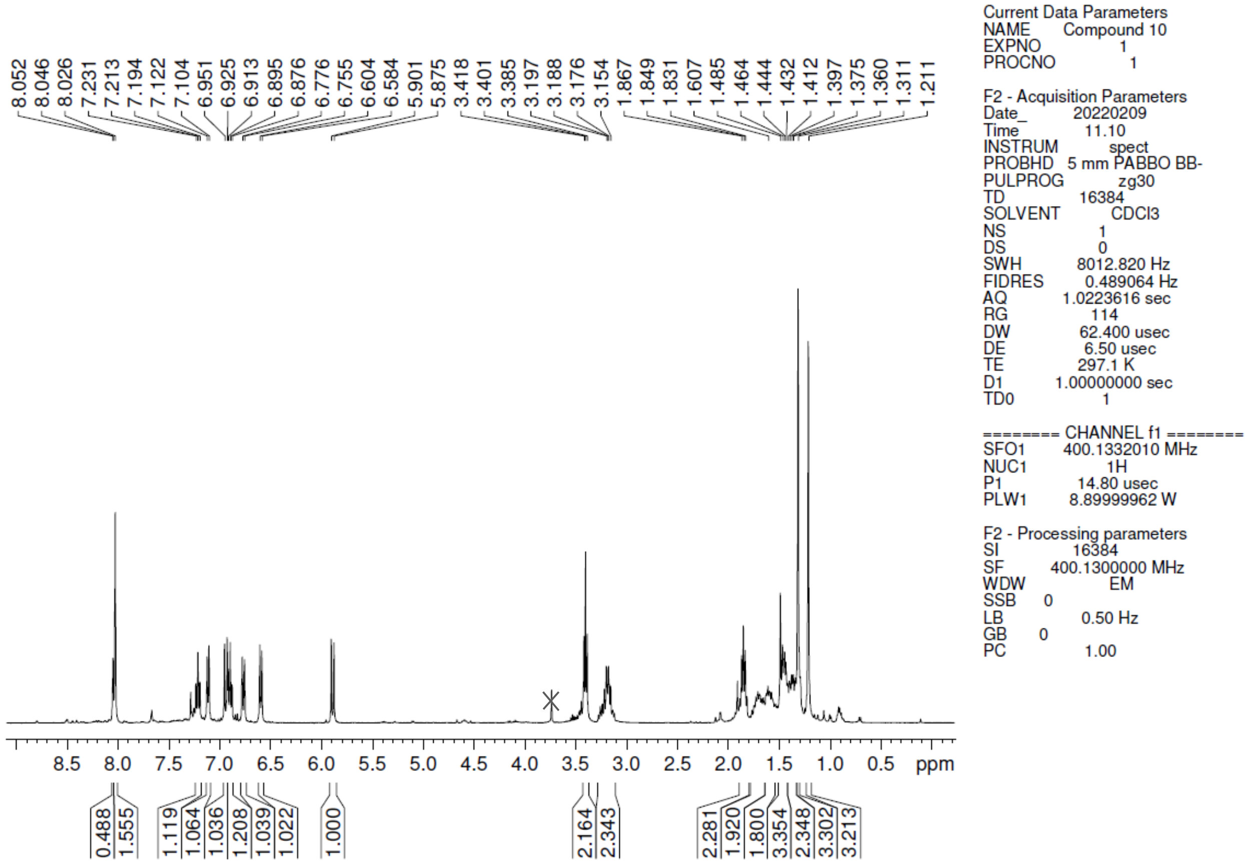

Figure S19. ^{1}H NMR spectra of compound **10** (400 MHz, $CDCl_3$).

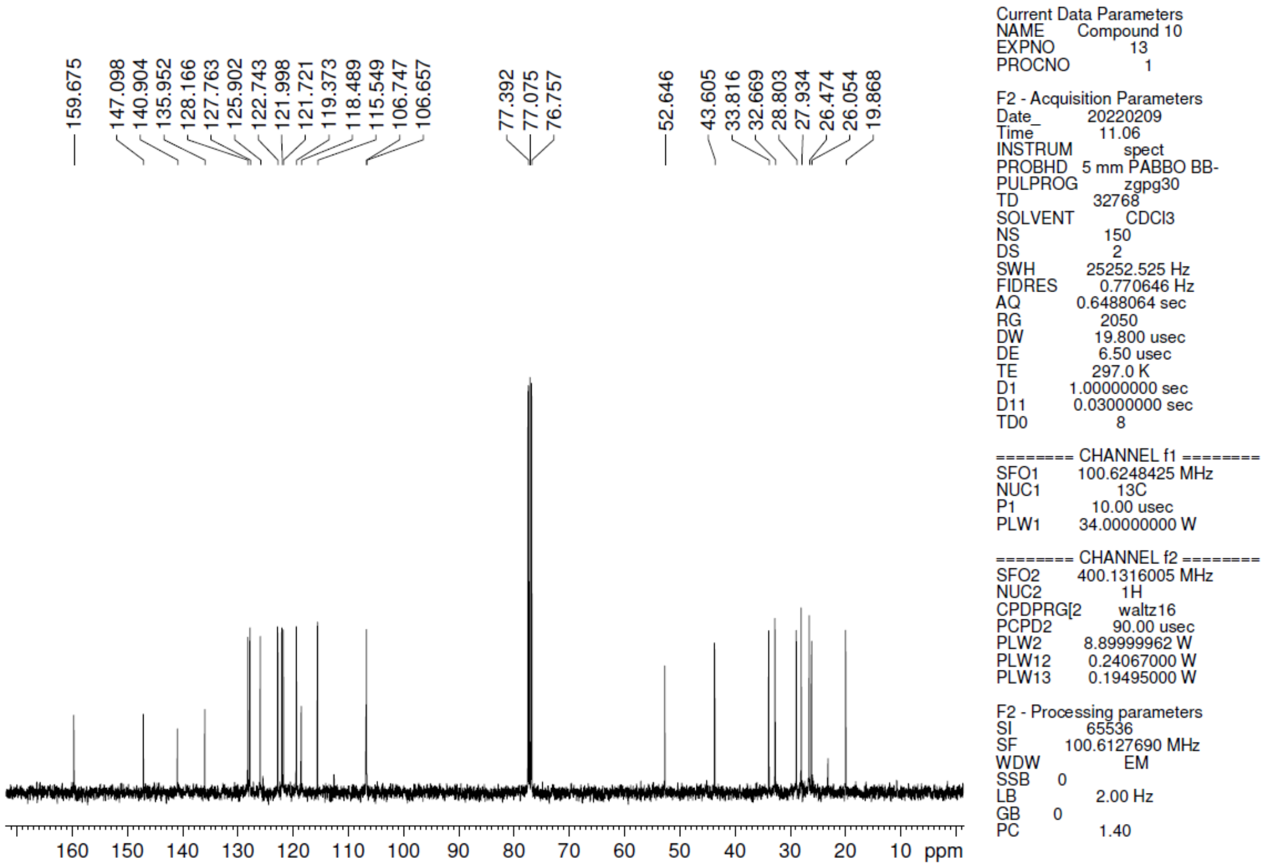

Figure S20. 13C NMR spectra of compound **10** (100 MHz, $CDCl_3$).

Compound 11: ^{1}H NMR (400 MHz, $CDCl_3$): δ = 1.20 (s, 3H), 1.30 (s, 9H), 1.42 (br. s, 2H), 1.56 (br. s, 1H), 1.67 (br. s, 2H), 1.85 (m, 2H), 1.16 (m, 2H), 3.41 (t, *J* = 6.6 Hz, 2H), 5.88 (d, *J* = 10.3 Hz, 1H), 6.59 (d, *J* = 7.7 Hz, 1H), 6.76 (d, *J* = 8.5 Hz, 1H), 6.90 (dd, *J* = 19.1, 9.0 Hz, 2H), 7.10 (d, *J* = 7.0 Hz, 1H), 7.21 (t, *J* = 7.5 Hz, 1H), 8.02 (s, 1H), 8.04 (s, 1H) ppm. ^{13}C NMR (100 MHz, $CDCl_3$): δ = 19.85, 26.04, 27.19, 28.04, 28.65, 28.91, 29.19, 32.70, 33.97, 43.73, 52.64, 106.65, 106.76, 115.54, 118.50, 119.28, 121.68, 122.07, 122.70, 125.86, 127.74, 128.07, 135.93, 140.87, 147.13, 159.71 ppm.

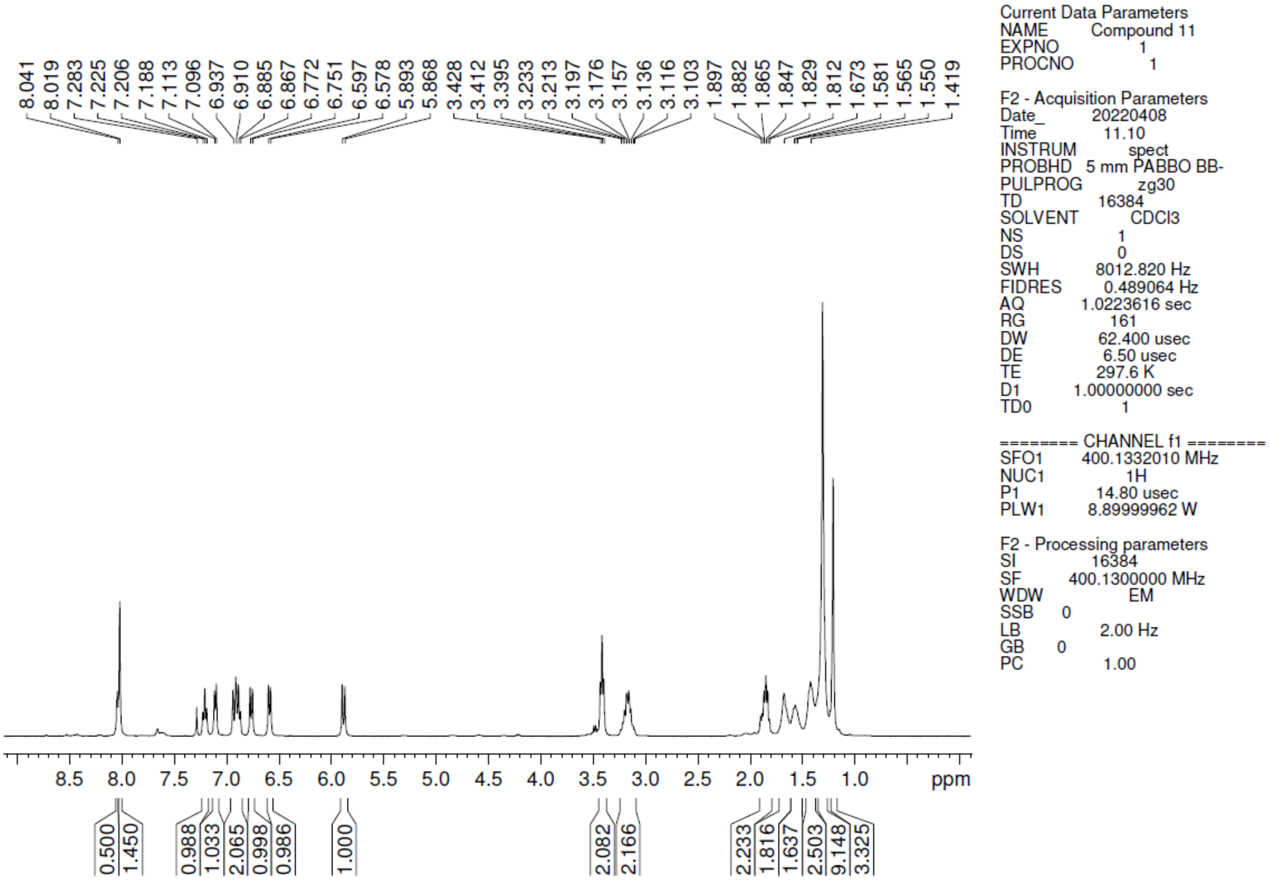

Figure S21. ^{1}H NMR spectra of compound **11** (400 MHz, $CDCl_3$).

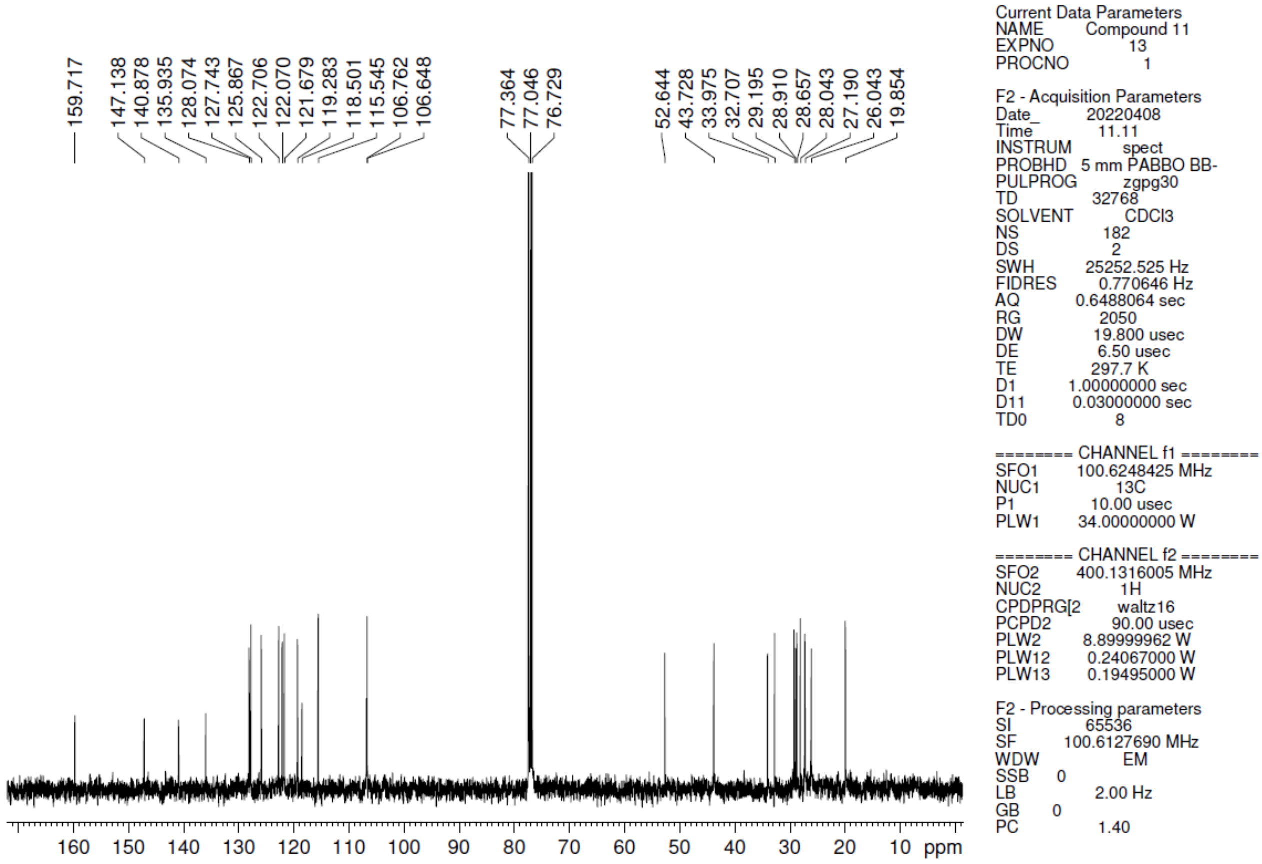

Figure S22. 13C NMR spectra of compound **11** (100 MHz, $CDCl_3$).

General procedure for the synthesis of compounds 12-16: A solution of compounds **7-11** (1.0 eq) and hexamethylenetetramine (4 eq) in CF_3CO_2H were refluxed for 1 h. After cooling, the mixture was neutralized, the organic phase was separated and distilled. Sequential purification via flash chromatography (petroleum ether/EtOAc (10:1); dichloromethane:ethanole 20:1) afforded compounds **12-16** (55-67%) as a light yellow solid.

Compound 12: ^{1}H NMR (400 MHz, $CDCl_3$): δ = 1.22 (s, 3H), 1.34 (s, 3H), 2.12 (m, 1H), 2.25 (m, 1H), 3.43 (m, 3H), 3.53 (m, 1H), 5.88 (d, J = 10.3 Hz, 1H), 6.74 (d, J = 8.1 Hz, 1H), 6.78 (d, J = 8.5 Hz, 1H), 7.01 (d, J = 10.3 Hz, 1H), 7.67 (s, 1H), 7.73 (d, J = 8.2 Hz, 1H), 8.04 (s, 1H), 8.07 (d, J = 2.5 Hz, 1H), 9.83 (s, 1H) ppm. ^{13}C NMR (100 MHz, $CDCl_3$): δ = 19.82, 25.88, 30.60, 31.46, 41.82, 52.11, 106.11, 106.16, 115.61, 118.20, 120.57, 122.02, 122.94, 126.17, 128.98, 129.62, 134.28, 137.10, 141.45, 152.08, 158.58, 190.50 ppm.

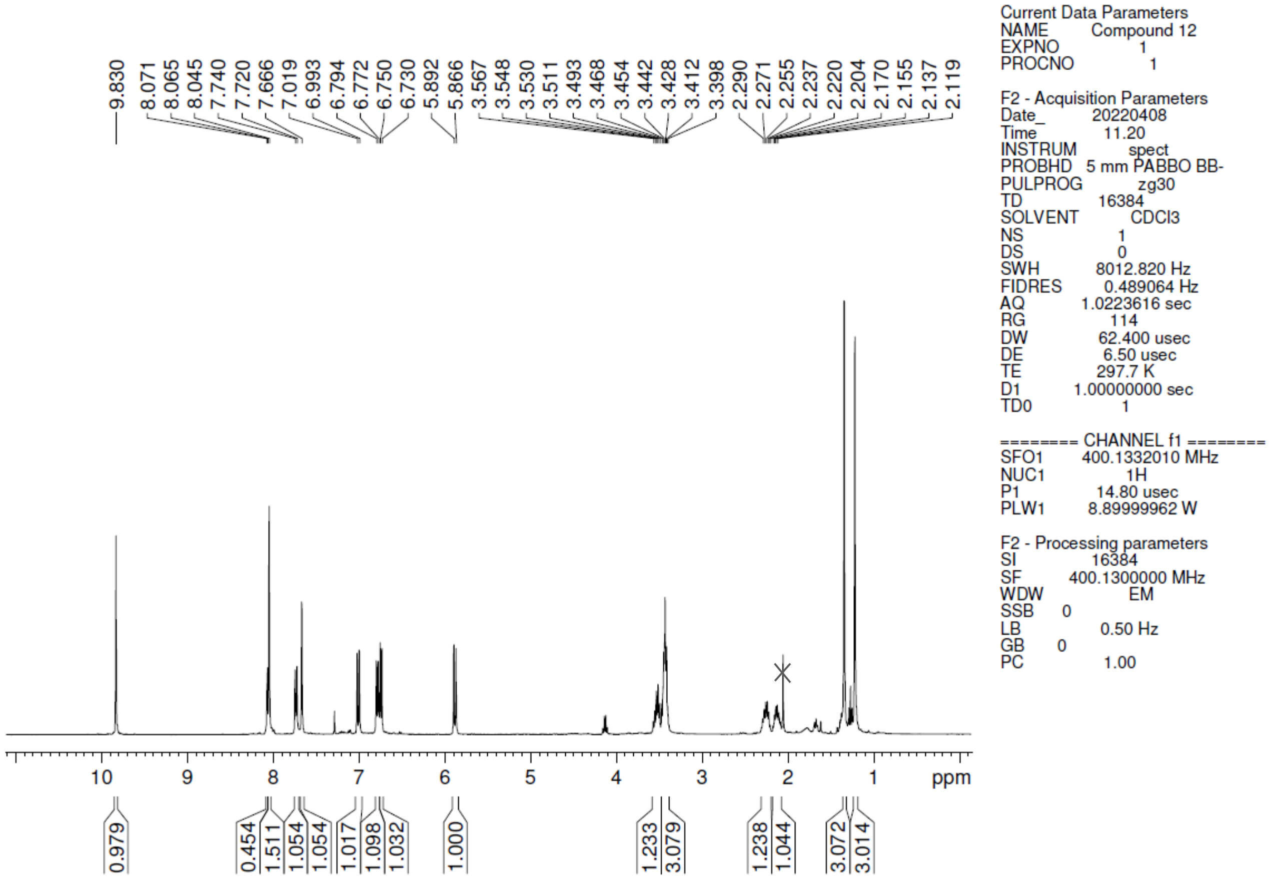

Figure S23. ^{1}H NMR spectra of compound **12** (400 MHz, $CDCl_3$).

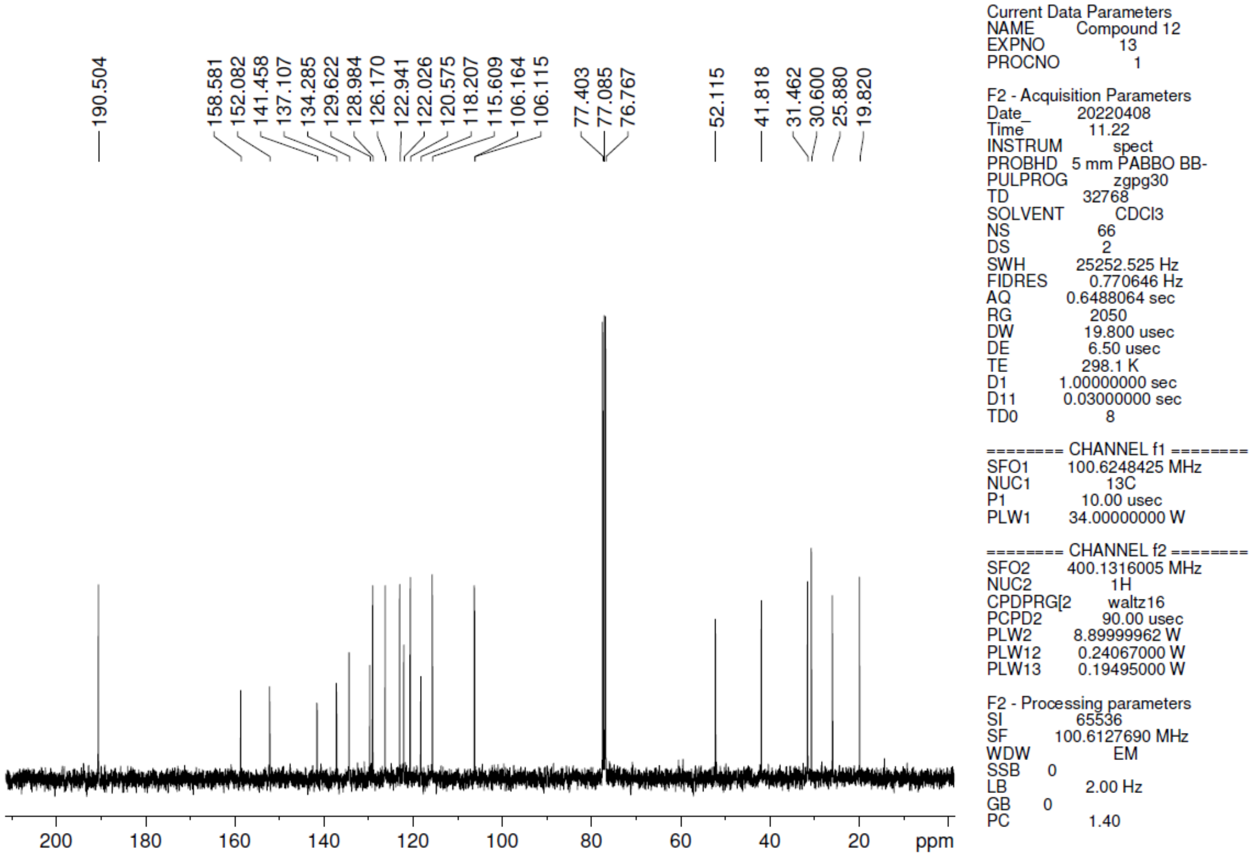

Figure S24. 13C NMR spectra of compound **12** (100 MHz, $CDCl_3$).

Compound 13: ^{1}H NMR (400 MHz, $CDCl_3$): δ = 1.22 (s, 3H), 1.34 (s, 3H), 1.85 (m, 4H), 3.32 (m, 2H), 3.39 (m, 2H), 5.88 (d, *J* = 10.3 Hz, 1H), 6.66 (d, *J* = 7.9 Hz, 1H), 6.78 (d, *J* = 8.4 Hz, 1H), 6.99 (d, *J* = 10.3 Hz, 1H), 7.66 (s, 1H), 7.73 (d, *J* = 7.8 Hz, 1H), 8.04 (s, 1H), 8.06 (s, 1H), 9.83 (s, 1H) ppm. ^{13}C NMR (100 MHz, $CDCl_3$): δ = 19.81, 25.93, 27.41, 30.19, 33.03, 42.73, 52.08, 106.00, 106.08, 115.63, 118.14, 120.67, 122.00, 122.91, 126.16, 128.79, 129.44, 134.30, 137.07, 141.39, 152.21, 158.69, 190.49 ppm.

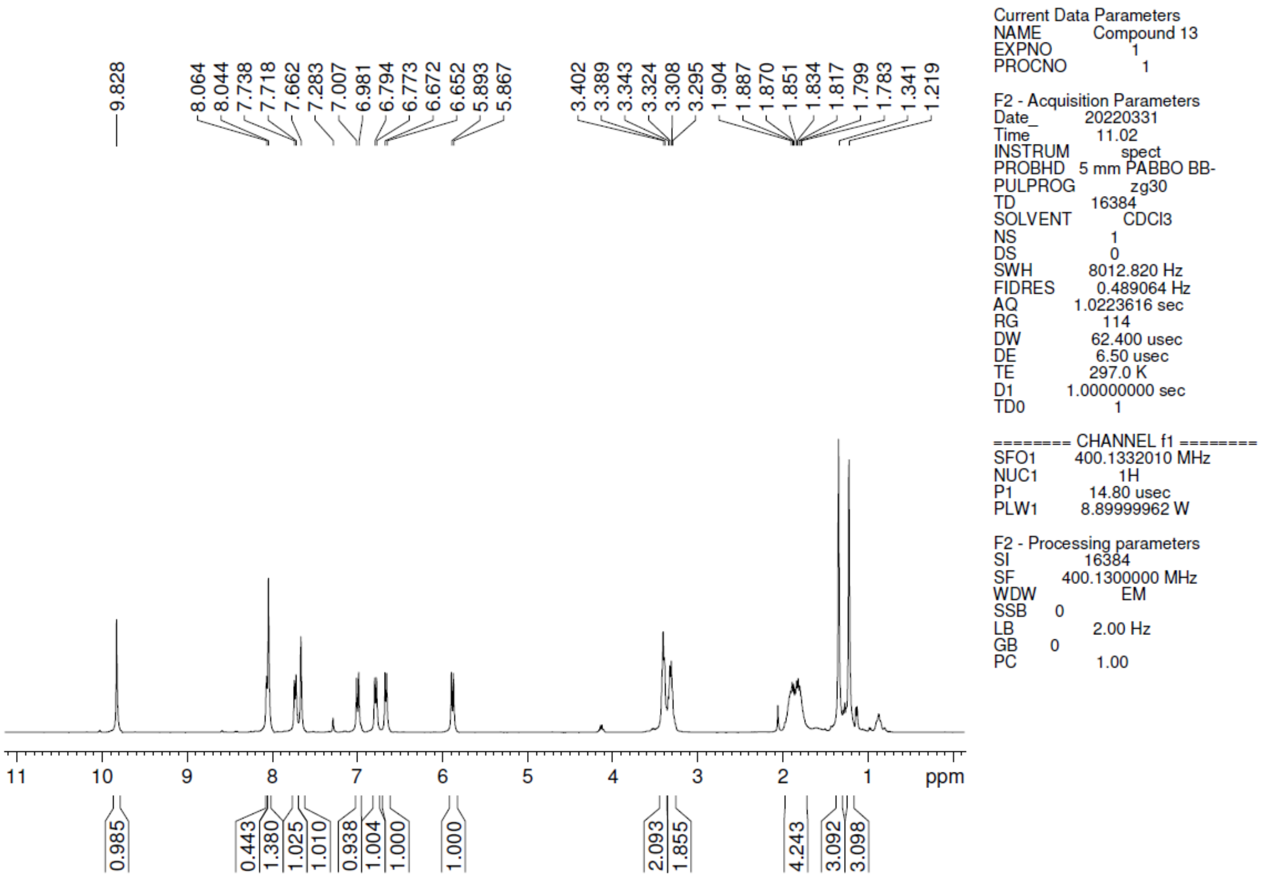

Figure S25. ^{1}H NMR spectra of compound **13** (400 MHz, $CDCl_3$).

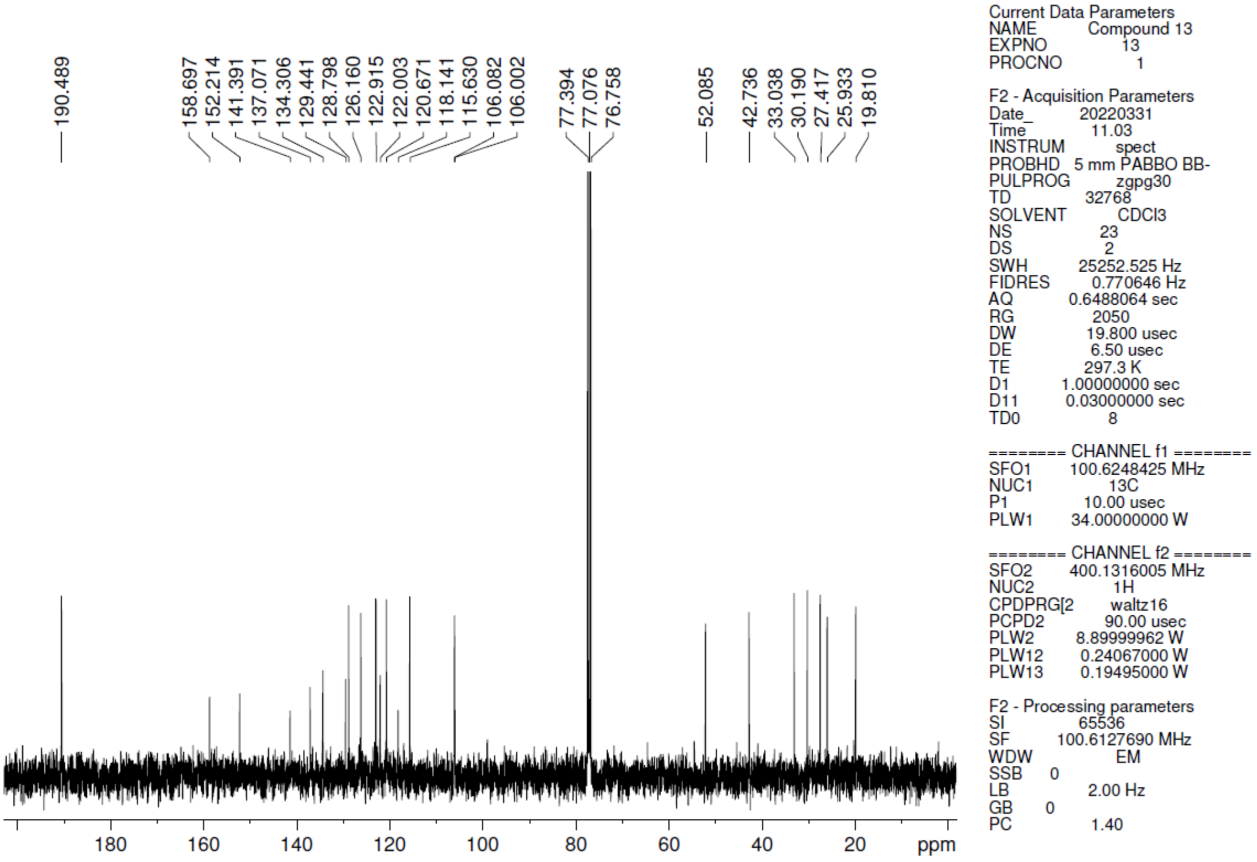

Figure S26. 13C NMR spectra of compound **13** (100 MHz, $CDCl_3$).

Compound 14: ^{1}H NMR (400 MHz, $CDCl_3$): δ = 1.22 (s, 3H), 1.34 (s, 3H), 1.49 (m, 2H), 1.60 (m, 1H), 1.69 (m, 1H), 1.86 (dt, J = 13.6, 6.7 Hz, 2H), 3.29 (dd, J = 13.8, 6.4 Hz, 1H), 3.39 (t, J = 6.5 Hz, 2H), 5.89 (d, J = 10.3 Hz, 1H), 6.64 (d, J = 8.0 Hz, 1H), 6.78 (d, J = 8.9 Hz, 1H), 6.99 (d, J = 10.3 Hz, 1H), 7.66 (s, 1H), 7.72 (d, J = 7.9 Hz, 1H), 8.04 (s, 1H), 8.06 (s, 1H), 9.82 (s, 1H) ppm. ^{13}C NMR (100 MHz, $CDCl_3$): δ = 19.79, 25.74, 25.94, 27.94, 32.21, 33.51, 43.33, 52.07, 105.96, 106.11, 115.59, 118.17, 120.78, 121.98, 122.91, 126.13, 128.72, 129.31, 134.31, 137.08, 141.35, 152.31, 158.75, 190.51 ppm.

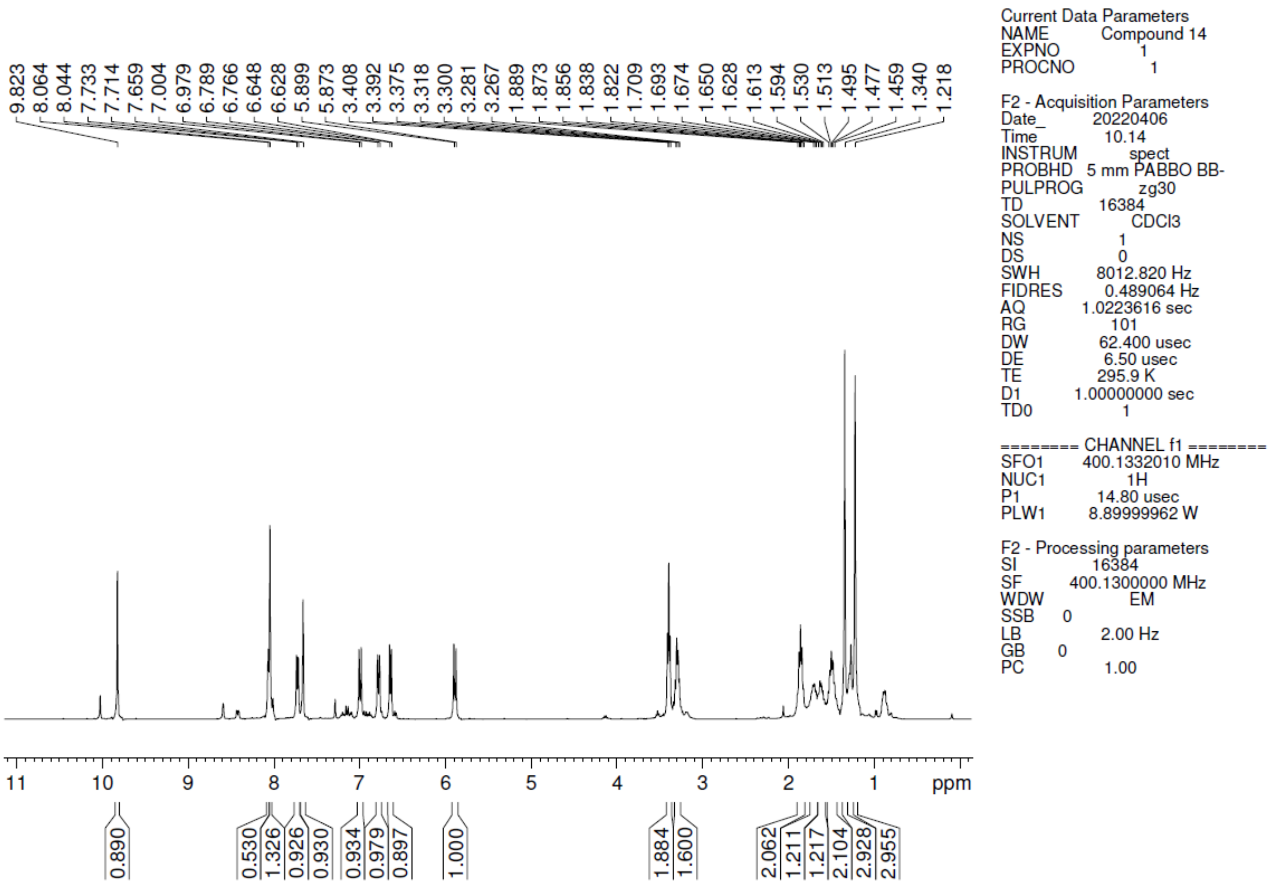

Figure S27. ^{1}H NMR spectra of compound **14** (400 MHz, $CDCl_3$).

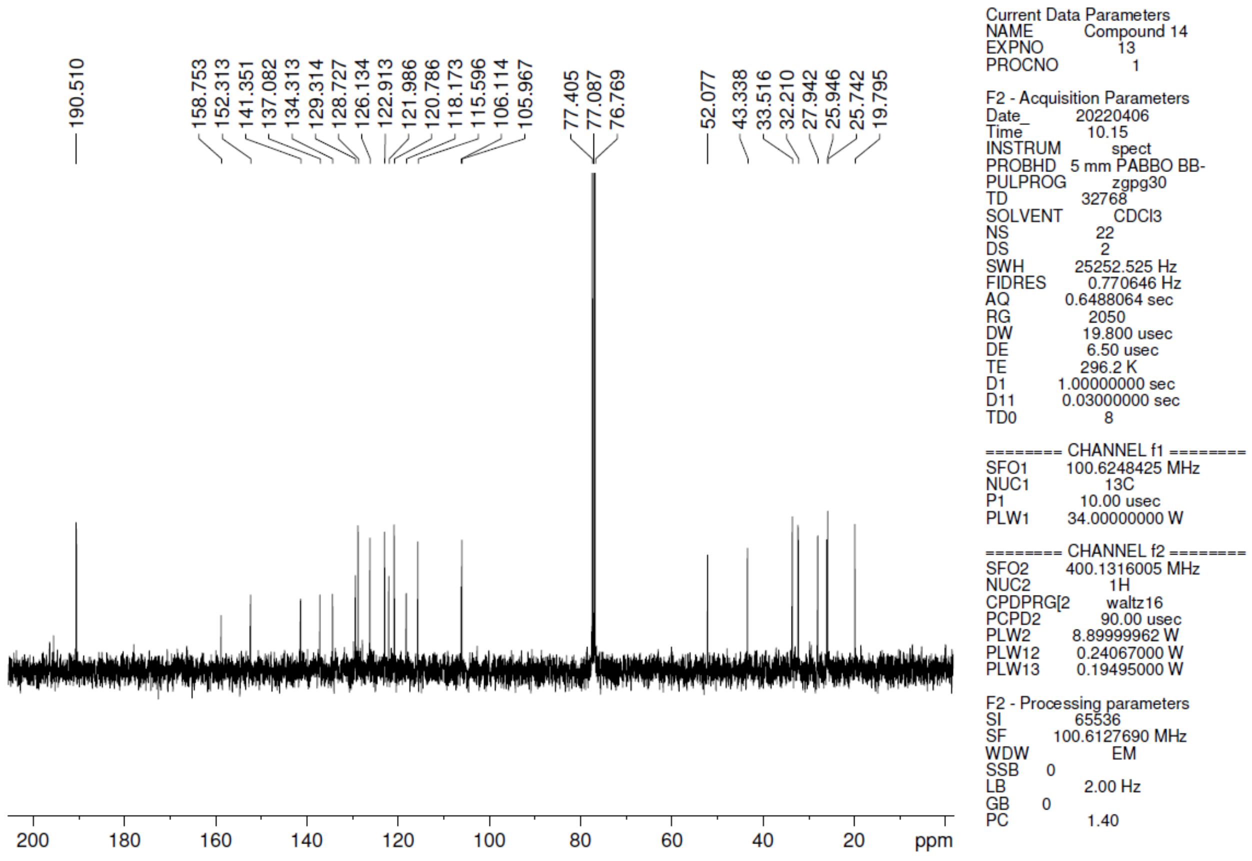

Figure S28. 13C NMR spectra of compound **14** (100 MHz, $CDCl_3$).

Compound 15: ^{1}H NMR (400 MHz, $CDCl_3$): δ = 1.20 (s, 3H), 1.32 (s, 3H), 1.42 (m, 2H), 1.59 (m, 1H), 1.68 (m, 1H), 1.81 (m, 2H), 3.27 (dd, *J* = 15.0, 7.0 Hz, 2H), 3.37 (t, *J* = 6.7 Hz, 2H), 5.87 (d, *J* = 10.3 Hz, 1H), 6.63 (d, *J* = 8.1 Hz, 1H), 6.76 (d, *J* = 9.7 Hz, 1H), 6.98 (d, *J* = 10.4 Hz, 1H), 7.66 (s, 1H), 7.64 (s, 1H), 7.70 (dd, *J* = 8.0, 1.2 Hz, 1H), 8.03 (dd, *J* = 5.6, 2.5 Hz, 2H), 9.80 (s, 1H) ppm. ^{13}C NMR (100 MHz, $CDCl_3$): δ = 19.77, 25.97, 26.37, 27.81, 28.62, 32.53, 33.72, 43.38, 52.06, 105.95, 106.09, 115.59, 118.18, 120.78, 121.95, 122.89, 126.11, 128.69, 129.25, 134.30, 137.05, 141.33, 152.36, 158.77, 190.47 ppm.

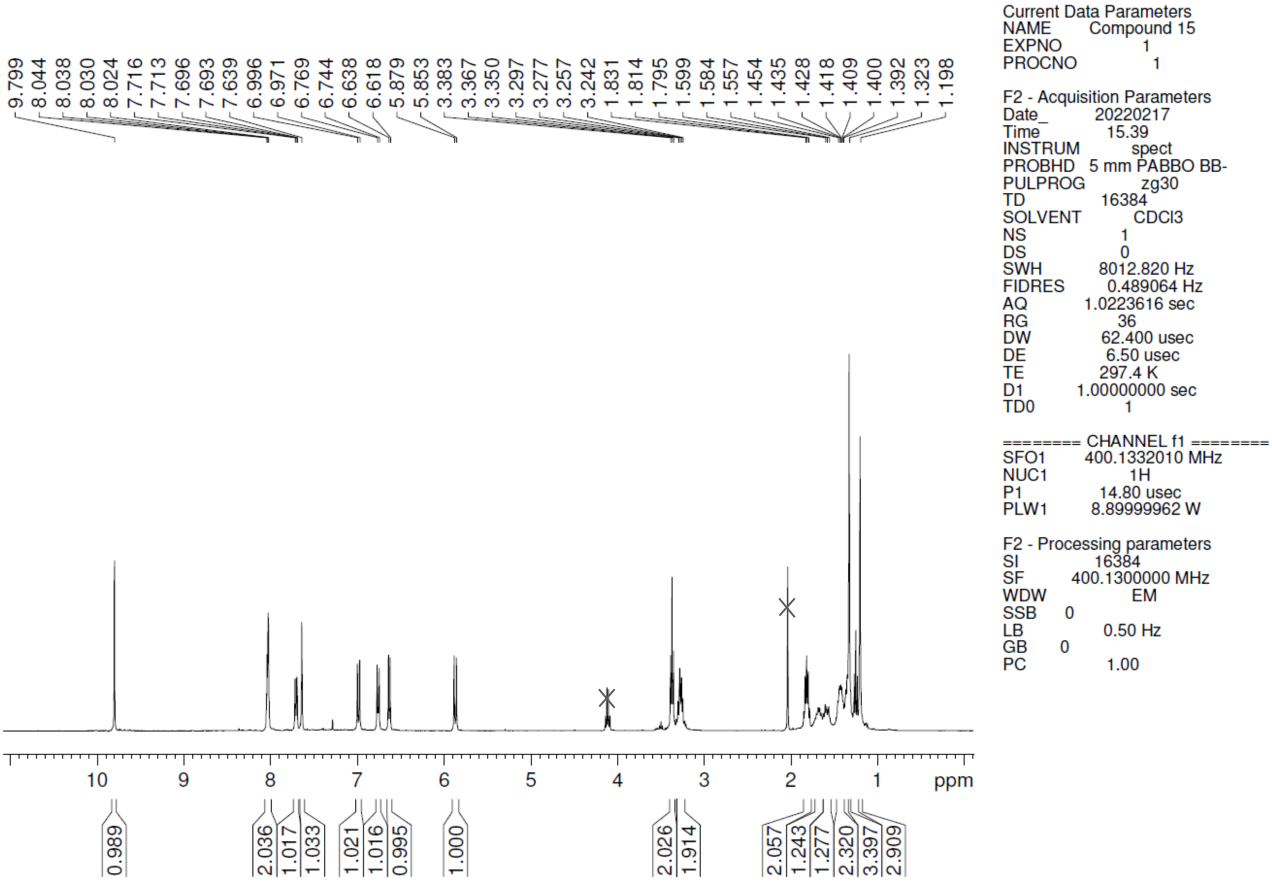

Figure S29. 1H NMR spectra of compound **15** (400 MHz, $CDCl_3$).

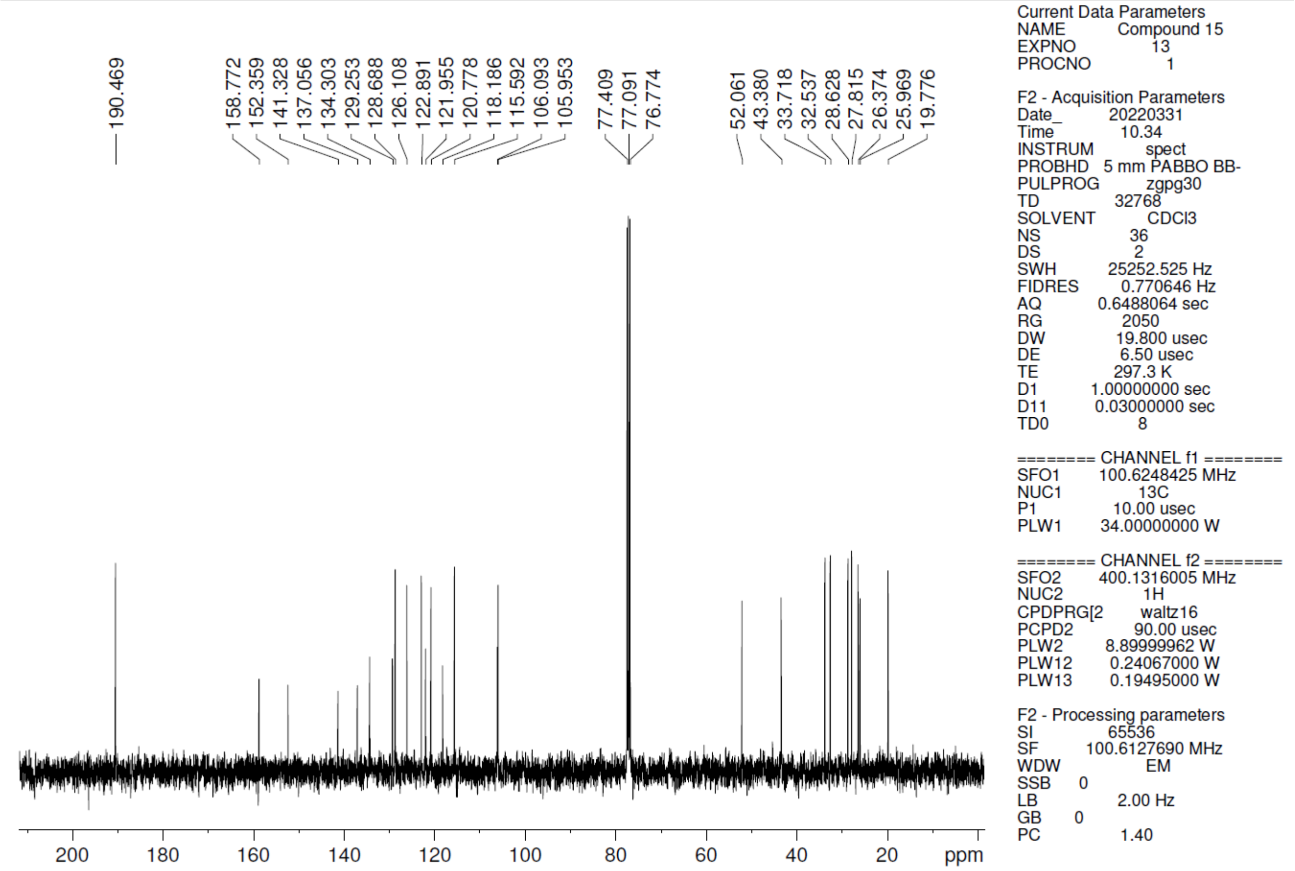

Figure S30. 13C NMR spectra of compound **15** (100 MHz, $CDCl_3$).

Compound 16: ^1H NMR (400 MHz, $CDCl_3$): δ = 1.20 (s, 3H), 1.32 (s, 3H), 1.29 (s, 6H), 1.32 (s, 3H), 1.38 (m, 2H), 1.55 (m, 1H), 1.65 (m, 1H), 1.80 (m, 2H), 3.25 (m, 2H), 3.37 (t, J = 6.8 Hz, 2H), 5.87 (d, J = 10.3 Hz, 1H), 6.63 (d, J = 8.1 Hz, 1H), 6.76 (d, J = 9.7 Hz, 1H), 6.98 (d, J = 10.4 Hz, 1H), 7.63 (s, 1H), 7.64 (s, 1H), 7.70 (d, J = 8.0 1H), 8.02 (dd, J = 6.2, 2.6 Hz, 2H), 9.79 (s, 1H) ppm. ^{13}C NMR (100 MHz, $CDCl_3$): δ = 19.77, 25.97, 27.07, 27.97, 28.56, 28.72, 29.08, 32.65, 33.97, 43.50, 52.04, 105.96, 106.11, 115.56, 118.25, 120.86, 121.92, 122.85, 126.01, 128.60, 129.17, 134.26, 137.04, 141.29, 152.41, 158.81, 190.39 ppm.

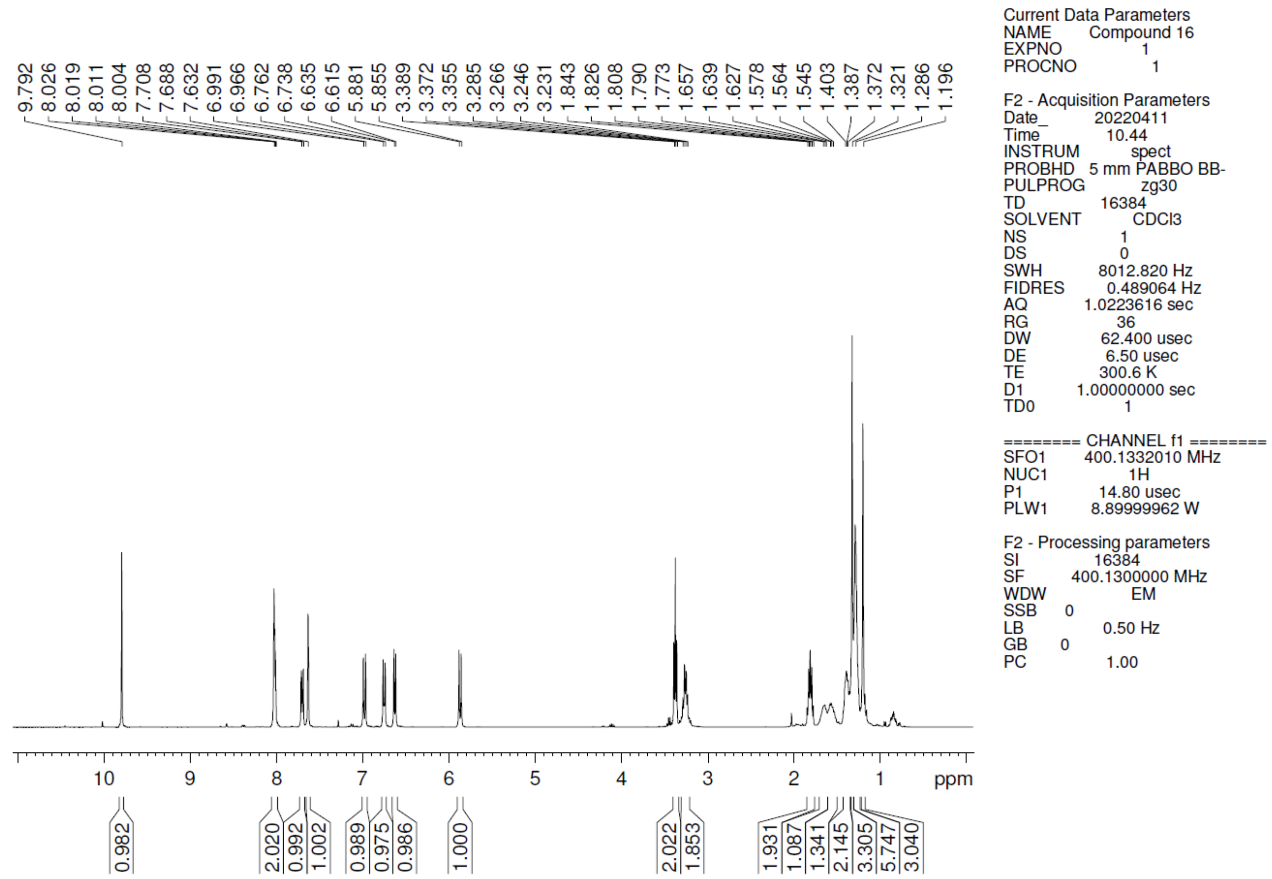

Figure S31. 1H NMR spectra of compound **16** (400 MHz, $CDCl_3$).

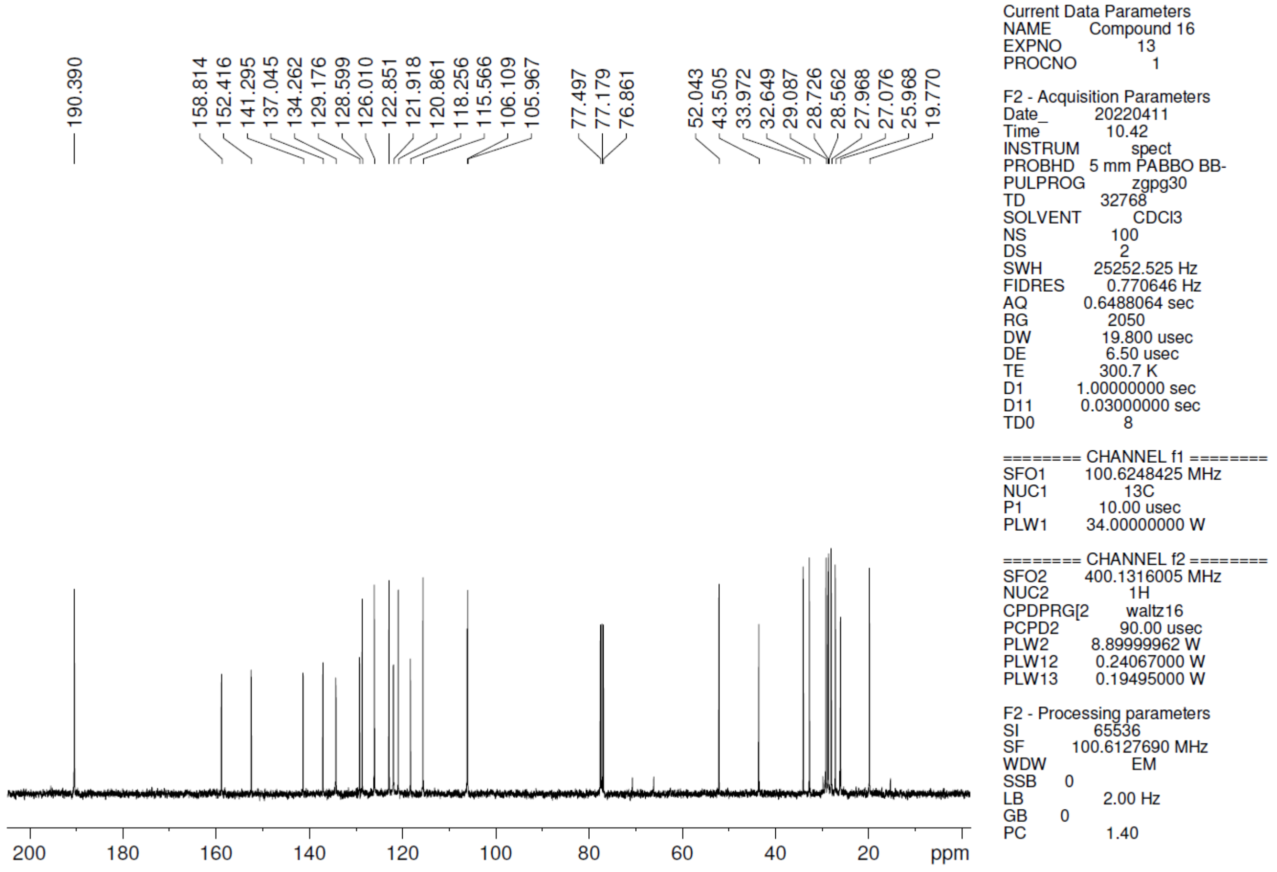

Figure S32. 13C NMR spectra of compound **16** (100 MHz, $CDCl_3$).

General procedure for the synthesis of compounds 17-21: To compounds 12-16 (1eq) in THF in a flask was added 2M Me2NH in THF solution (10 eq). The mixture was then stirred at room temperature overnight. After the solvent was removed under reduced pressure, the resultant residue was purified through silica-gel column chromatography eluting with dichloromethane/ethanol (20:1) to afford the corresponding products **17-21** (yields 70-80%).

Compound 17: ^{1}H NMR (400 MHz, $CDCl_3$): δ = 1.19 (s, 3H), 1.32 (s, 3H), 1.80 (m, 2H), 2.21 (s, 6H), 2.32 (t, *J* = 7.0 Hz, 2H), 3.35 (m, 2H), 5.87 (d, *J* = 10.4 Hz, 1H), 6.70 (d, *J* = 8.1 Hz, 1H), 6.75 (d, *J* = 9.6 Hz, 1H), 6.96 (d, *J* = 10.4 Hz, 1H), 7.63 (s, 1H), 7.70 (dd, *J* = 8.0, 1.1 Hz, 1H), 8.01 (s, 1H), 8.04 (d, *J* = 2.5 Hz, 1H), 9.80 (s, 1H) ppm. ^{13}C NMR (100 MHz, $CDCl_3$): δ = 19.76, 25.90, 26.77, 41.40, 45.25, 52.11, 56.90, 106.04, 106.15, 115.57, 118.23, 120.84, 121.92, 122.84, 126.04, 128.60, 129.27, 134.29, 136.98, 141.32, 152.41, 158.77, 190.44 ppm.

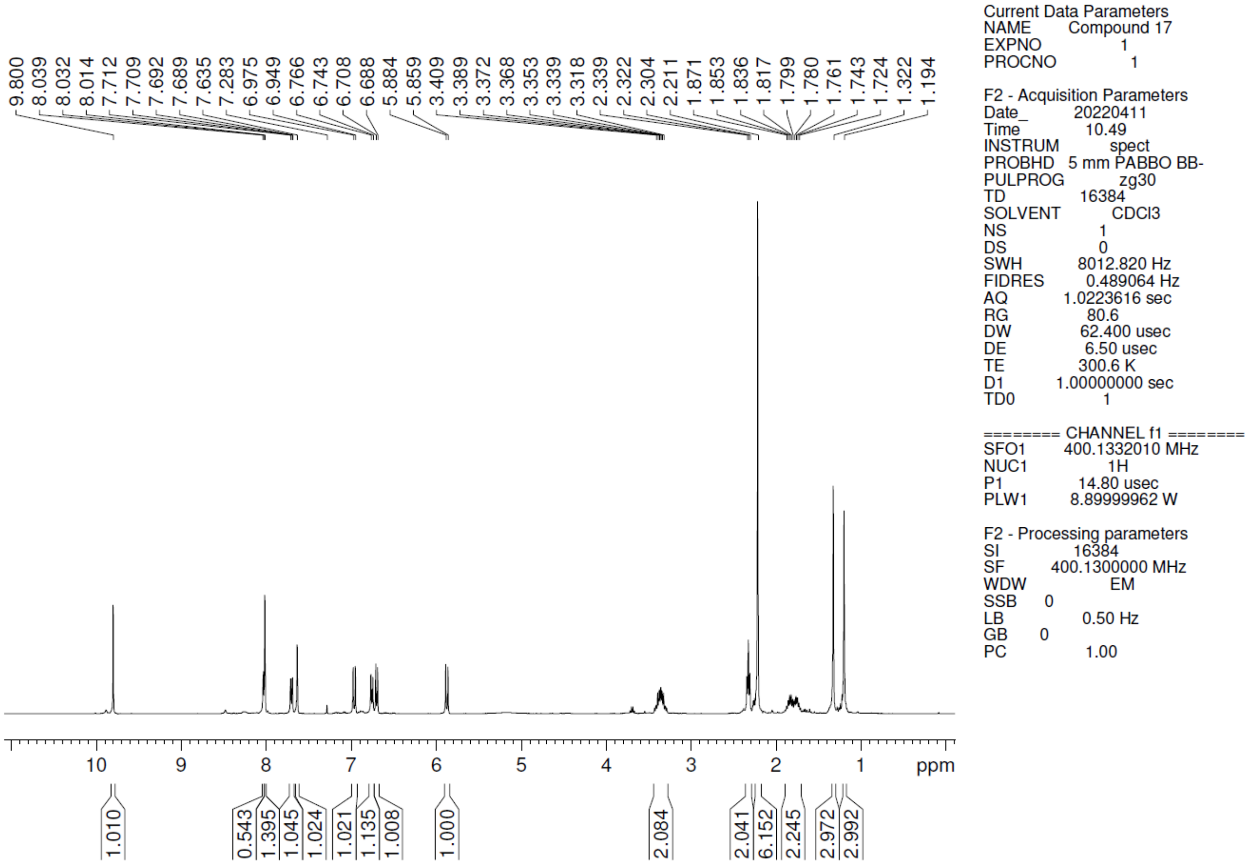

Figure S33. 1H NMR spectra of compound **17** (400 MHz, $CDCl_3$).

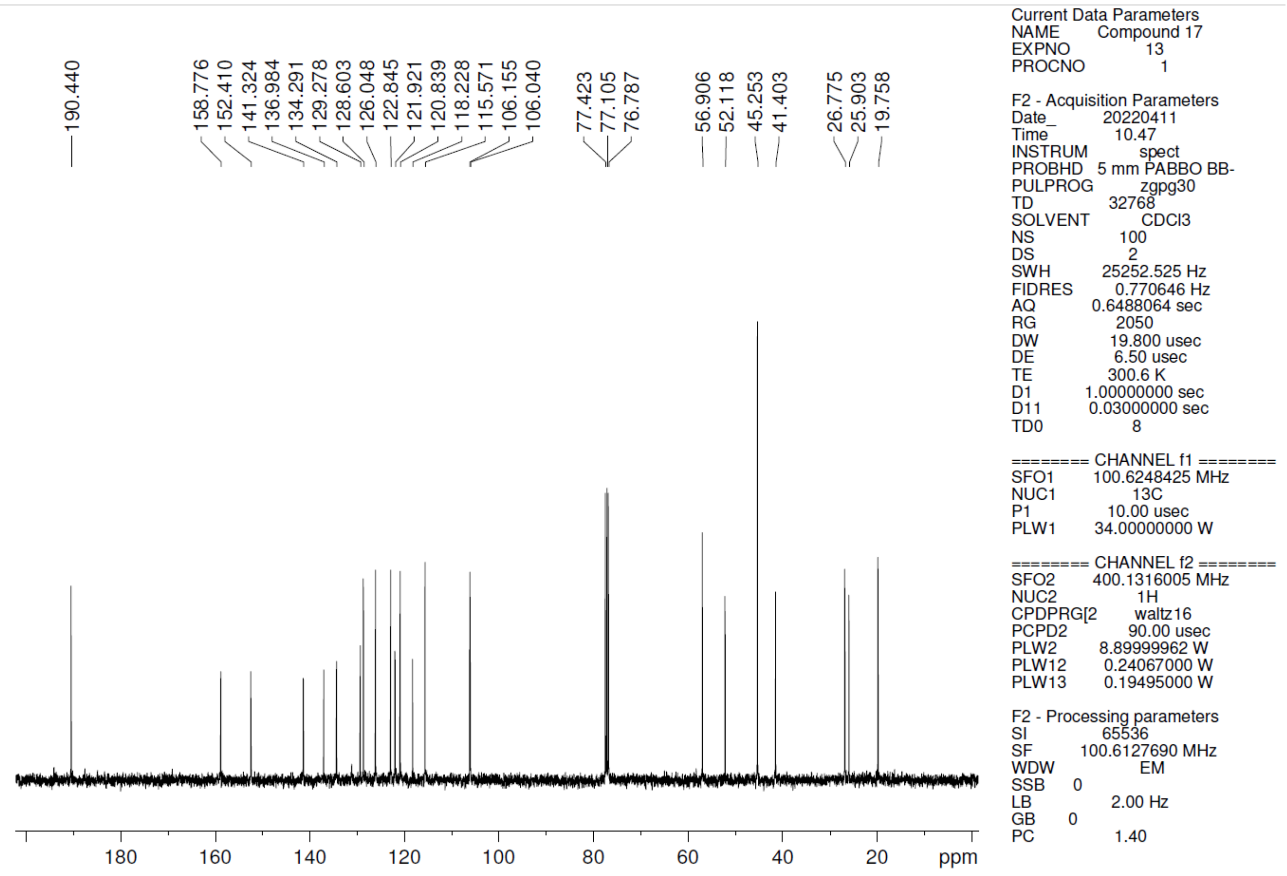

Figure S34. 13C NMR spectra of compound **17** (100 MHz, $CDCl_3$).

Compound 18: ^{1}H NMR (400 MHz, $CDCl_3$): δ = 1.17 (s, 3H), 1.30 (s, 3H), 1.55 (m, 4H), 2.20 (s, 6H), 2.27 (t, J = 7.2 Hz, 2H), 3.26 (m, 2H), 5.86 (d, J = 10.3 Hz, 1H), 6.63 (d, J = 8.1 Hz, 1H), 6.73 (d, J = 9.7 Hz, 1H), 6.95 (d, J = 10.4 Hz, 1H), 7.61 (s, 1H), 7.68 (d, J = 8.1, 1H), 8.00 (dd, J = 4.4, 2.0 Hz, 2H), 9.77 (s, 1H) ppm. ^{13}C NMR (100 MHz, $CDCl_3$): δ = 19.75, 25.16, 25.91, 26.61, 43.39, 45.23, 52.03, 59.09, 106.03, 106.11, 115.56, 118.22, 120.83, 121.89, 122.84, 126.01, 128.62, 129.20, 134.27, 137.02, 141.26, 152.37, 158.79, 190.45 ppm.

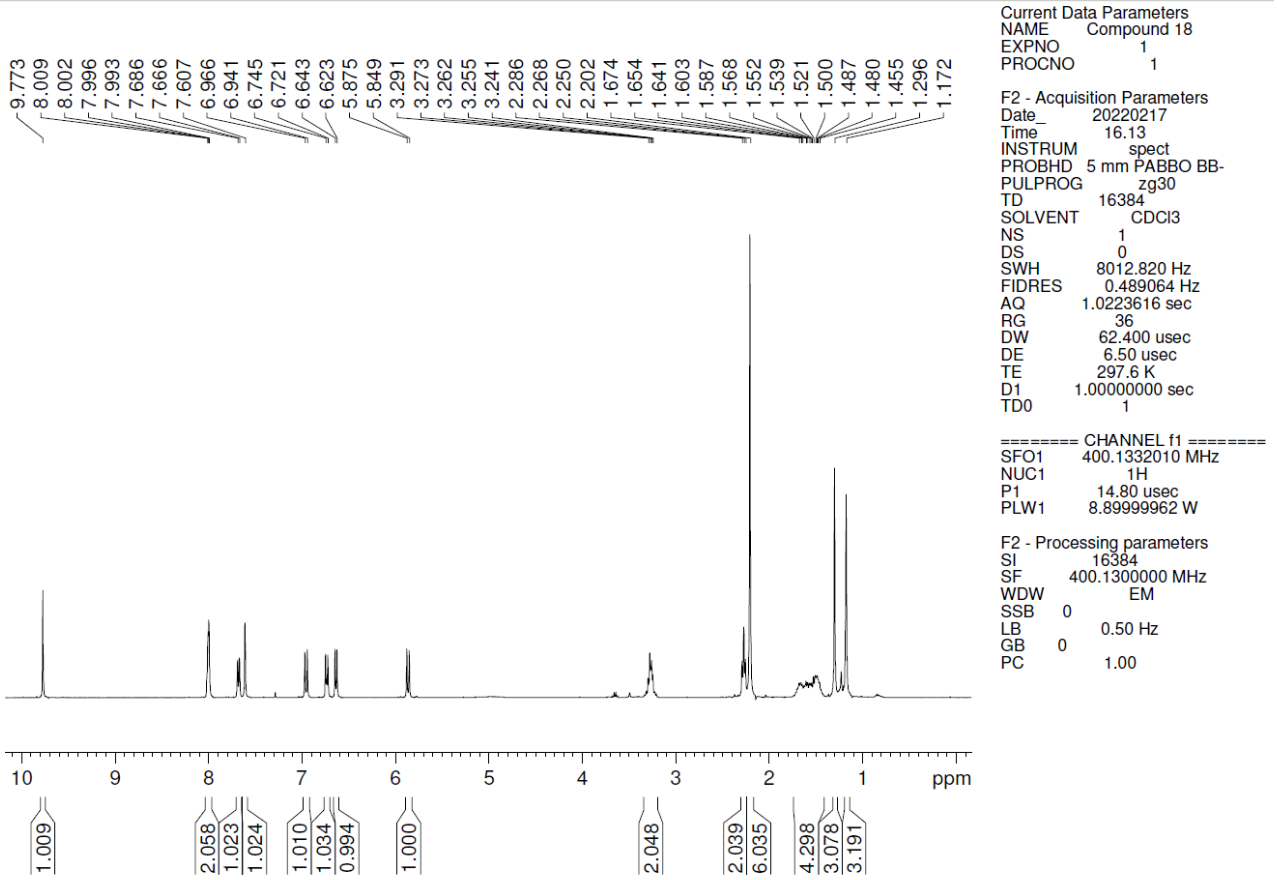

Figure S35. 1H NMR spectra of compound **18** (400 MHz, $CDCl_3$).

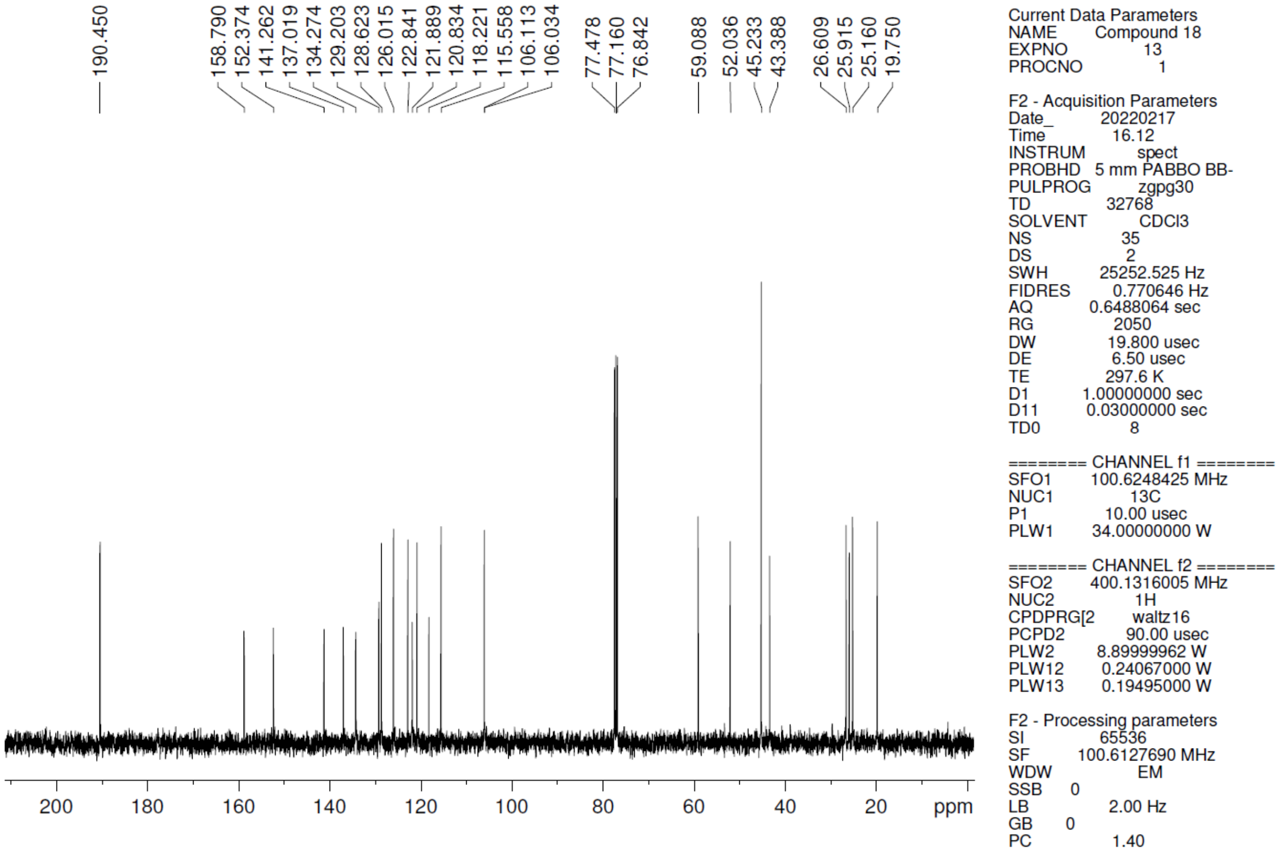

Figure S36. 13C NMR spectra of compound **18** (100 MHz, $CDCl_3$).

Compound 19: ^{1}H NMR (400 MHz, $CDCl_3$): δ = 1.20 (s, 3H), 1.25 (d, *J* = 8.5 Hz, 1H), 1.32 (s, 3H), 1.66 (m, 4H), 2.42 (s, 6H), 2.52 (m, 2H), 3.31 (m, 2H), 5.90 (d, *J* = 10.3 Hz, 1H), 6.65 (d, *J* = 8.0 Hz, 1H), 6.73 (d, *J* = 8.5 Hz, 1H), 6.98 (d, *J* = 10.3 Hz, 1H), 7.64 (s, 1H), 7.71 (d, *J* = 8.0, 1H), 8.03 (s, 1H), 8.05 (s, 1H), 9.81 (s, 1H) ppm. ^{13}C NMR (100 MHz, $CDCl_3$): δ = 19.66, 24.80, 25.88, 26.67, 28.55, 43.30, 44.85, 51.95, 59.06, 105.97, 106.03, 115.47, 118.23, 120.75, 121.82, 122.81, 125.90, 128.62, 129.05, 134.20, 136.96, 141.14, 152.31, 158.73, 190.32 ppm.

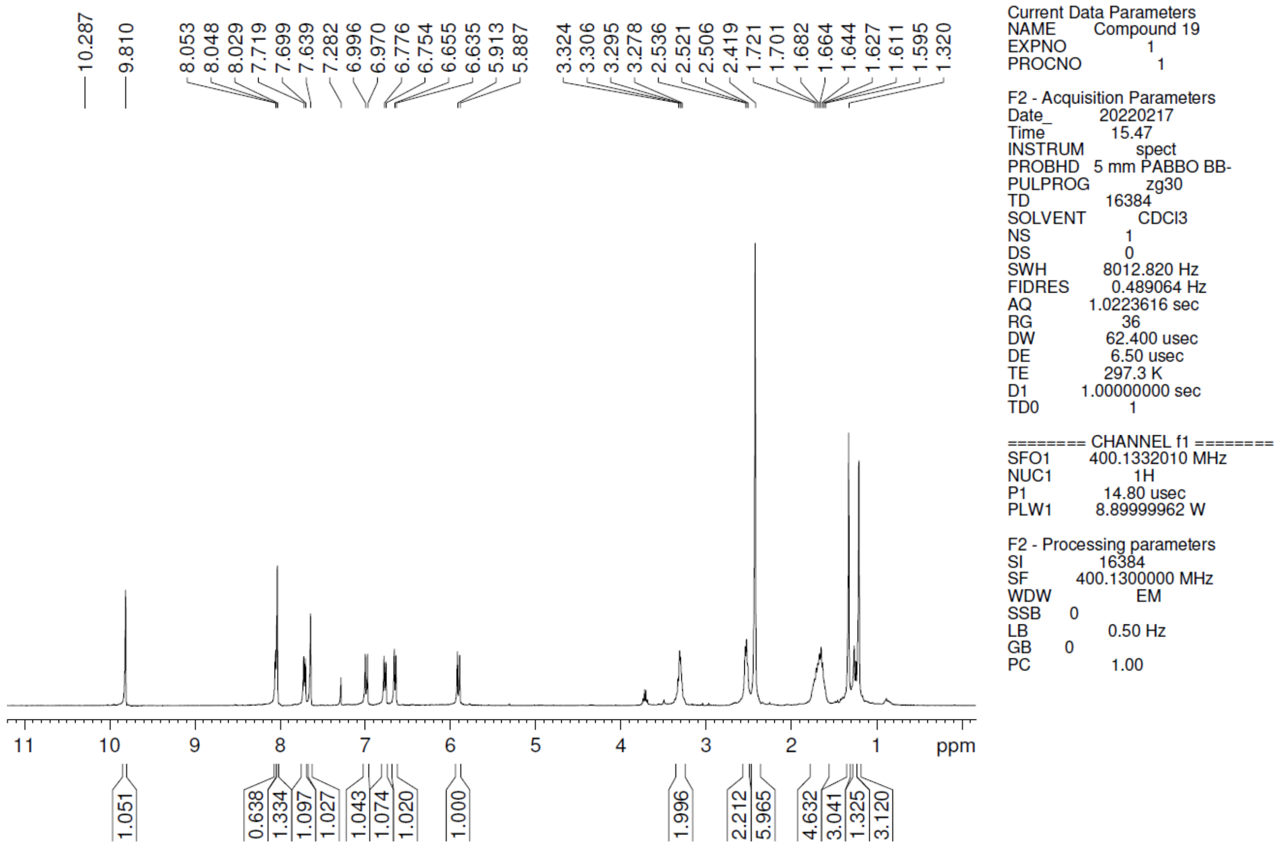

Figure S37. 1H NMR spectra of compound **19** (400 MHz, $CDCl_3$).

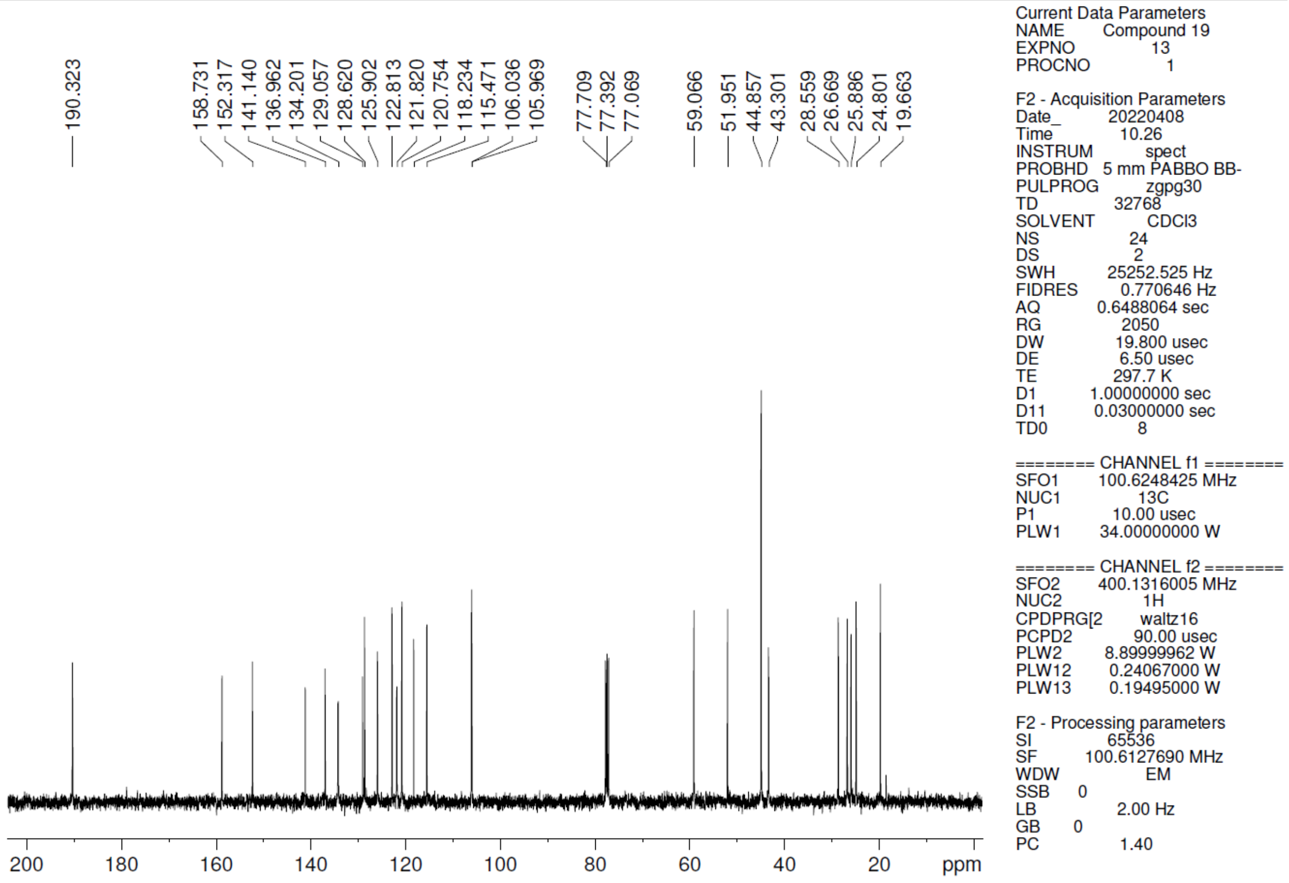

Figure S38. 13C NMR spectra of compound **19** (100 MHz, $CDCl_3$).

Compound 20: ^{1}H NMR (400 MHz, $CDCl_3$): δ = 1.19 (s, 3H), 1.32 (s, 6H), 1.48 (m, 2H), 1.57 (m, 1H), 1.66 (m, 1H), 2.28 (s, 6H), 2.31 (m, 2H), 3.24 (m, 2H), 5.86 (d, J = 10.4 Hz, 1H), 6.62 (d, J = 8.1 Hz, 1H), 6.76 (d, J = 9.2 Hz, 1H), 6.97 (d, J = 10.3 Hz, 1H), 7.64 (s, 1H), 7.70 (d, J = 8.1, 1H), 8.03 (s, 1H), 8.05 (d, J = 2.5 Hz, 1H), 9.80 (s, 1H) ppm. ^{13}C NMR (100 MHz, $CDCl_3$): δ = 19.76, 25.95, 27.03, 27.13, 27.17, 28.73, 43.48, 45.09, 52.05, 59.39, 105.95, 106.10, 115.58, 118.19, 120.84, 121.90, 122.86, 126.07, 128.61, 129.18, 134.32, 137.03, 141.29, 152.40, 158.80, 190.48 ppm.

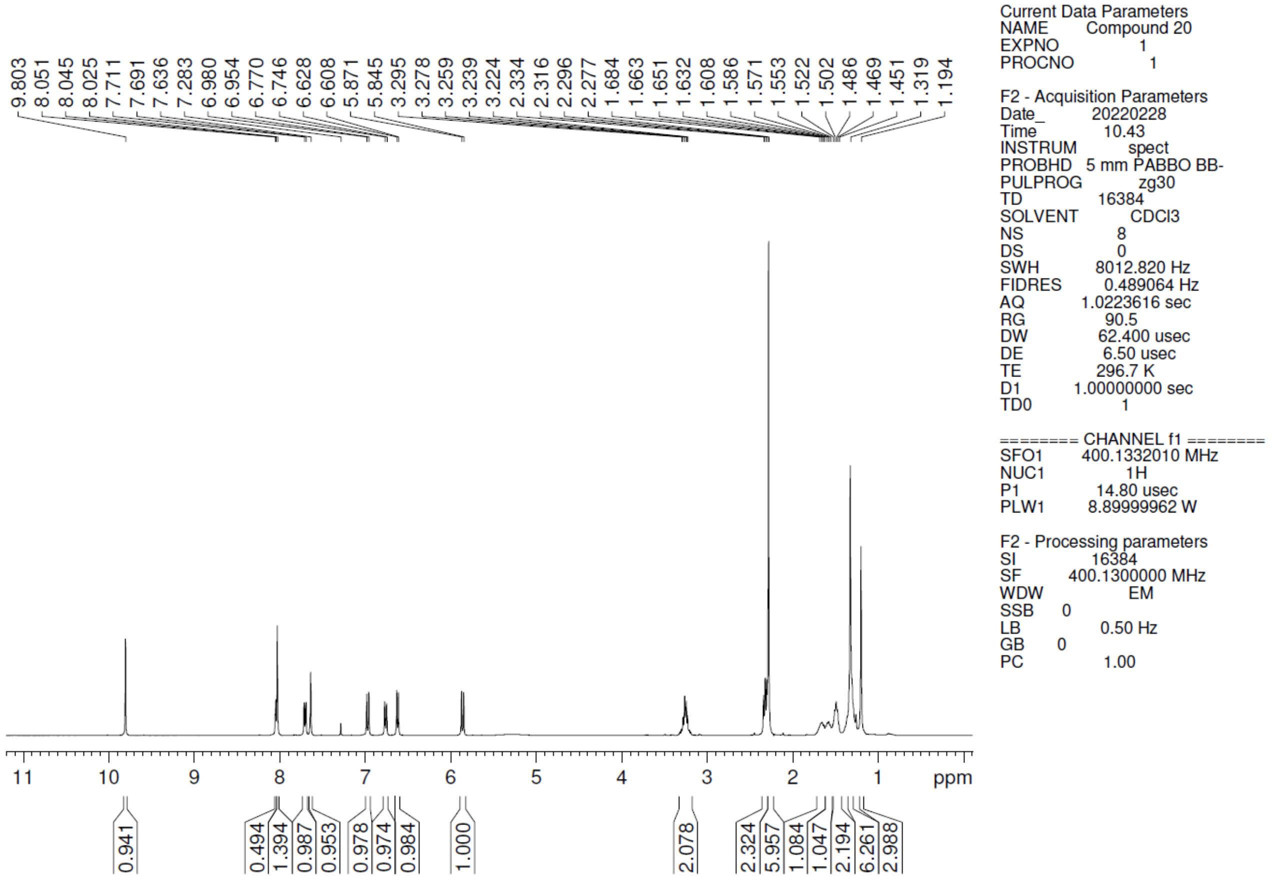

Figure S39. 1H NMR spectra of compound **20** (400 MHz, $CDCl_3$).

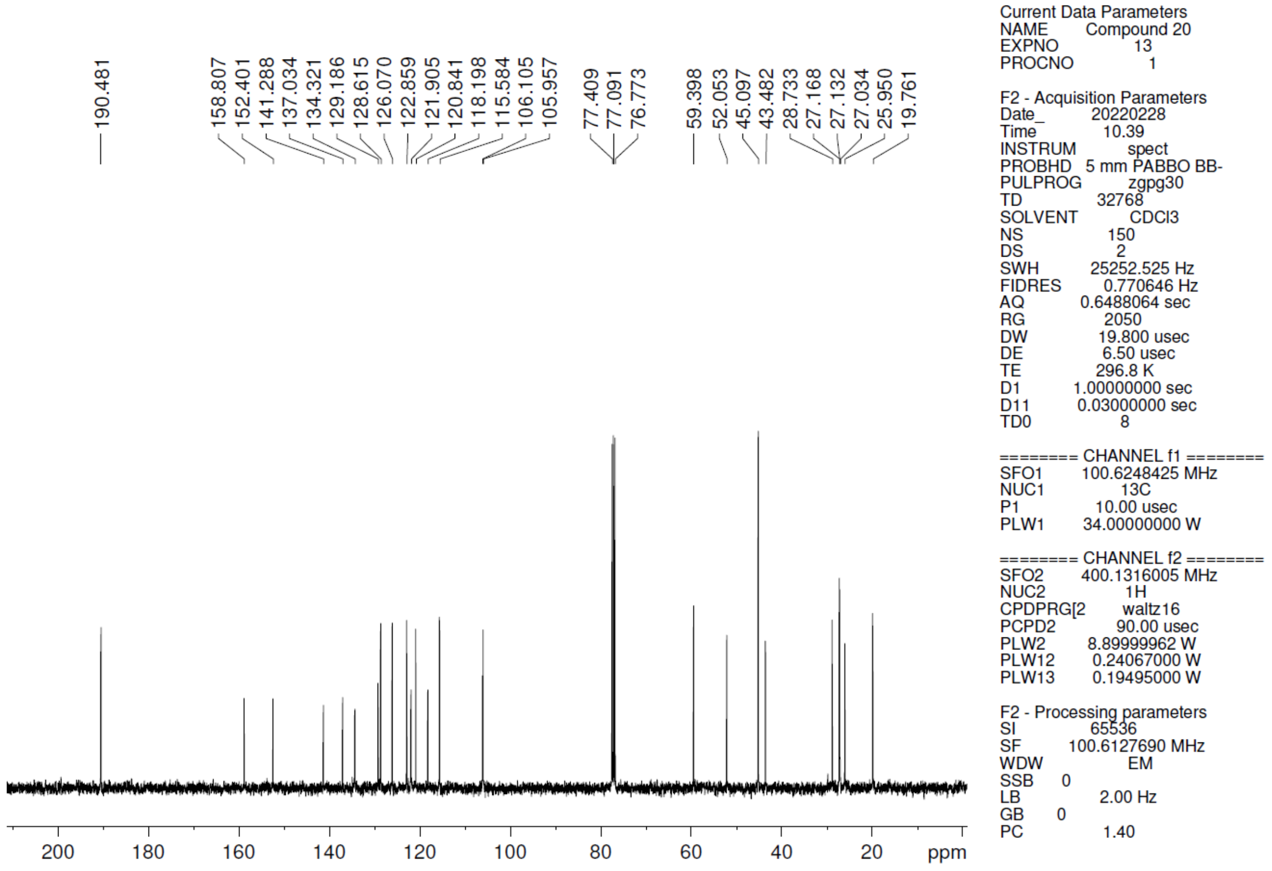

Figure S40. 13C NMR spectra of compound **20** (100 MHz, $CDCl_3$).

Compound 21: ^{1}H NMR (500 MHz, $CDCl_3$): δ = 1.19 (s, 3H), 1.26 (br. s, 8H), 1.31 (s, 3H), 1.53 (m, 3H), 1.64 (m, 1H), 2.33 (s, 6H), 2.38 (t, *J* = 7.5 Hz, 2H), 3.23 (m, 2H), 5.85 (d, *J* = 10.3 Hz, 1H), 6.61 (d, *J* = 8.1 Hz, 1H), 6.75 (d, *J* = 9.7 Hz, 1H), 6.97 (d, *J* = 10.4 Hz, 1H), 7.63 (s, 1H), 7.70 (d, *J* = 8.1, 1H), 8.03 (dd, *J* = 6.1, 2.5 Hz, 2H), 9.80 (s, 1H) ppm. ^{13}C NMR (125 MHz, $CDCl_3$): δ = 19.75, 25.97, 26.93, 26.57, 27.12, 27.16, 28.74, 29.18, 29.32, 43.50, 44.89, 52.02, 59.41, 105.94, 106.07, 115.58, 118.22, 120.81, 121.88, 122.85, 126.04, 128.59, 129.15, 134.31, 137.03, 141.27, 152.40, 158.81, 190.45 ppm.

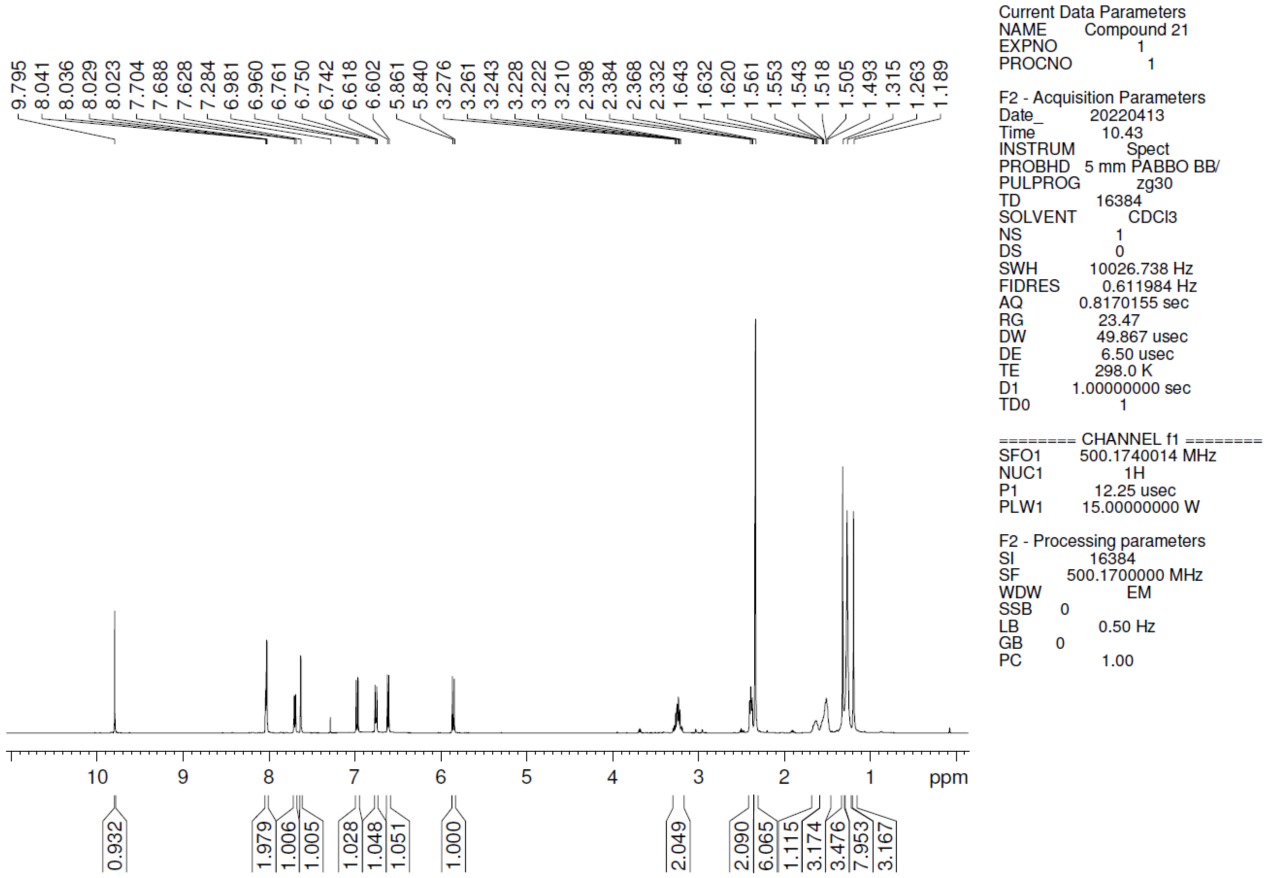

Figure S41. 1H NMR spectra of compound **21** (500 MHz, $CDCl_3$).

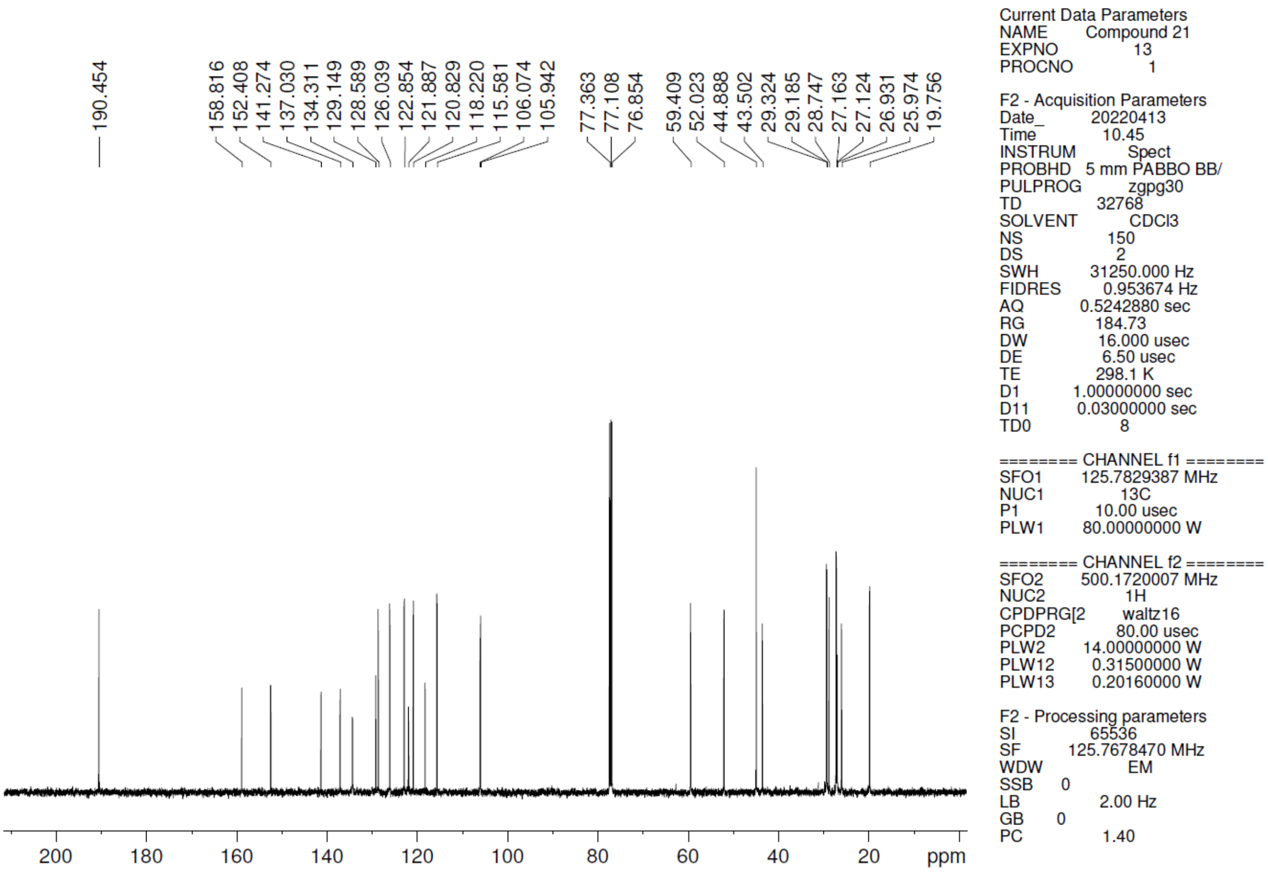

Figure S42. 13C NMR spectra of compound **21** (125 MHz, $CDCl_3$).

General procedure for the synthesis of compounds 22-26: Compouds **17-21** (1 eq) was dissolved in EtOH. The solution was cooled on an ice-bath and 0.2 M HBr in EtOH (1 eq) was added dropwise. After the solvent was removed under reduced pressure, the resultant residue was washed with diethyl ether to afford the corresponding products **22-26** (yield, 90-95%).

Compound 22: ^{1}H NMR (500 MHz, $CDCl_3$): δ = 1.19 (s, 3H), 1.30 (s, 3H), 2.03(m, 2H), 2.52 (s, 5H), 2.75 (t, J = 6.7 Hz, 2H), 3.35 (m, 1H), 3.43 (m, 1H), 5.93 (d, J = 10.3 Hz, 1H), 6.74 (t, J = 8.0 Hz, 2H), 6.99 (d, J = 10.3 Hz, 1H), 7.61 (s, 1H), 7.69 (d, J = 7.6, 1H), 8.00 (s, 2H), 9.78 (s, 1H) ppm. ^{13}C NMR (125 MHz, $CDCl_3$): δ = 19.81, 25.45, 25.93, 41.01, 44.03, 52.15, 56.20, 106.15, 106.19, 115.55, 118.25, 120.77, 122.09, 122.96, 126.11, 128.88, 129.52, 134.24, 136.99, 141.36, 152.09, 158.64, 190.50 ppm.

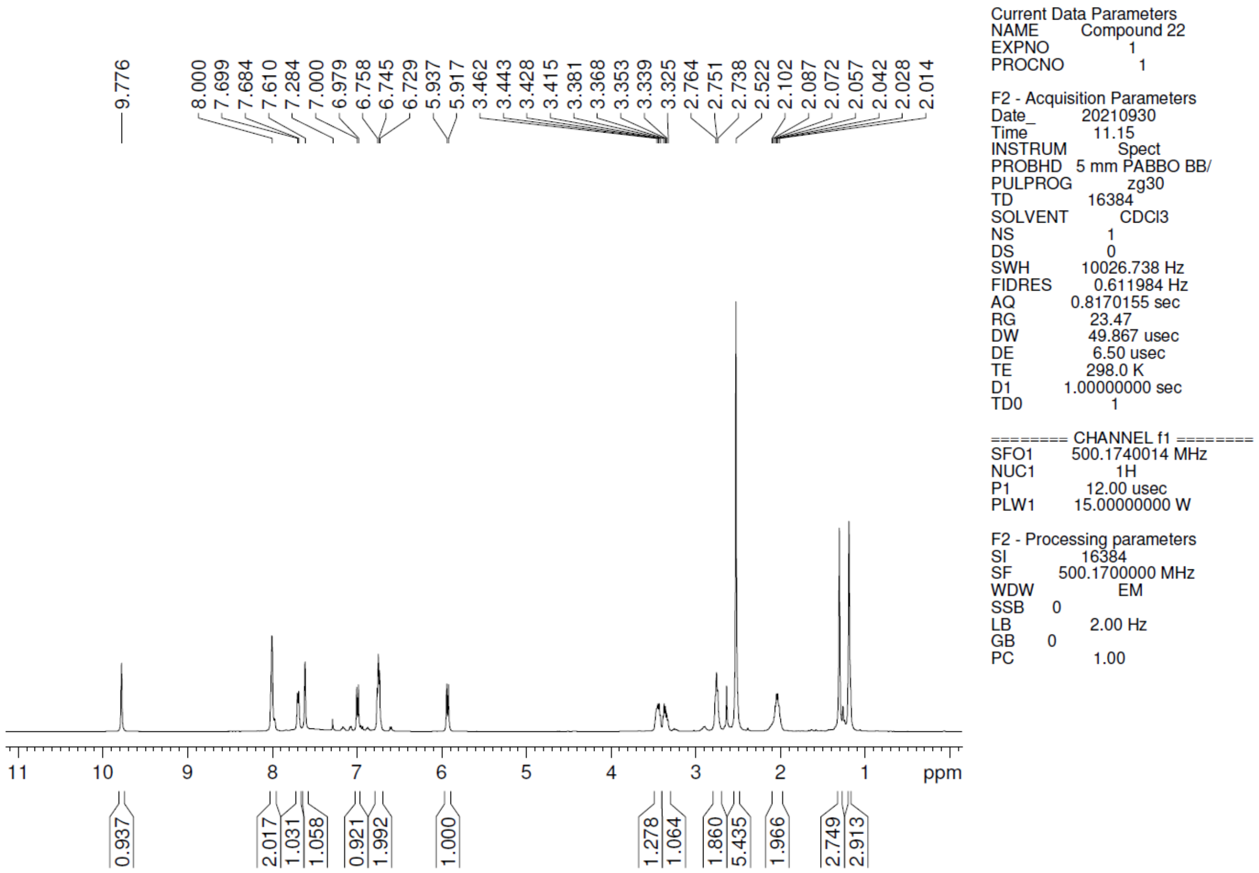

Figure S43. 1H NMR spectra of compound **22** (500 MHz, $CDCl_3$).

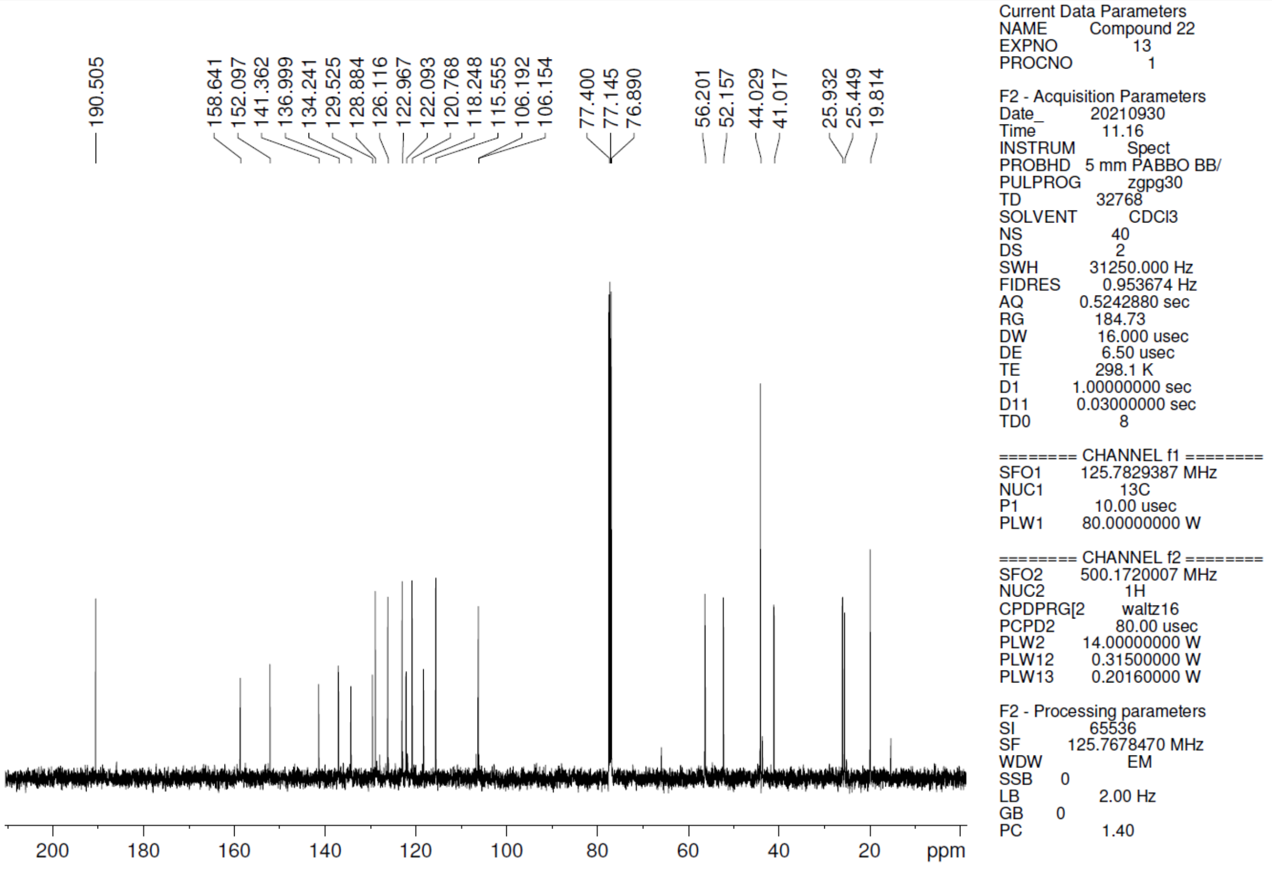

Figure S44. 13C NMR spectra of compound **22** (125 MHz, $CDCl_3$).

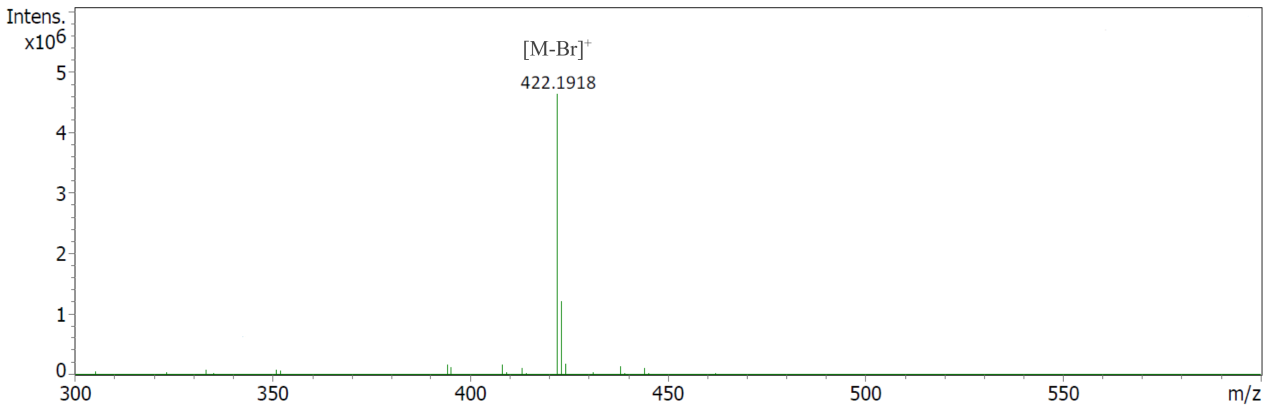

Figure S45. ESI-HRMS spectra of compound **22**.

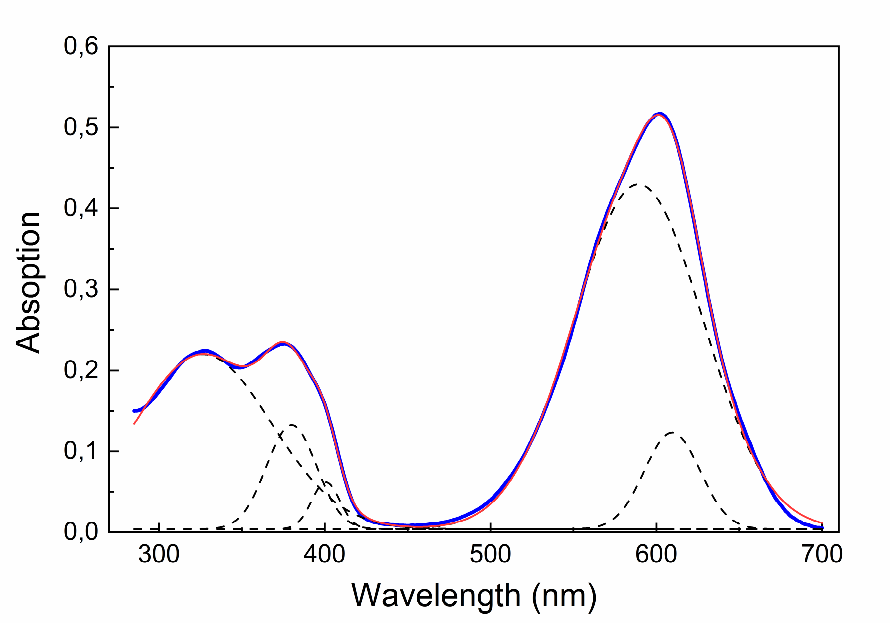

Figure S46. Decomposition of the experimental absorption spectrum of compound **22** (blue line) in the open merocyanine form into individual Gaussian bands (dash lines). The red curve is the sum of the individual Gaussian bands.The program used is the Origin2018.

Compound 23: ^{1}H NMR (400 MHz, $CDCl_3$): δ = 1.19 (s, 3H), 1.30 (s, 3H), 1.69 (m, 1H), 1.77 (m, 1H), 1.94 (m, 2H), 2.82 (d, *J* = 4.9 Hz, 6H), 3.04 (dt, *J* = 15.3, 7.7 Hz, 2H), 3.31 (m, 1H), 5.94 (d, *J* = 10.3 Hz, 1H), 6.64 (d, *J* = 8.1 Hz, 1H), 6.74 (d, *J* = 10.4 Hz, 1H), 7.01 (d, *J* = 10.4 Hz, 1H), 7.61 (s, 1H), 7.69 (d, *J* = 7.9 Hz, 1H), 8.00 (m, 2H), 9.79 (s, 1H) ppm. ^{13}C NMR (100 MHz, $CDCl_3$): δ = 19.81, 22.34, 25.95, 42.88, 43.04, 43.08, 52.13, 57.72, 106.12, 106.16, 115.56, 118.23, 120.82, 122.22, 123.00, 126.13, 129.00, 129.52, 134.06, 137.10, 141.35, 151.97, 158.63, 190.53 ppm.

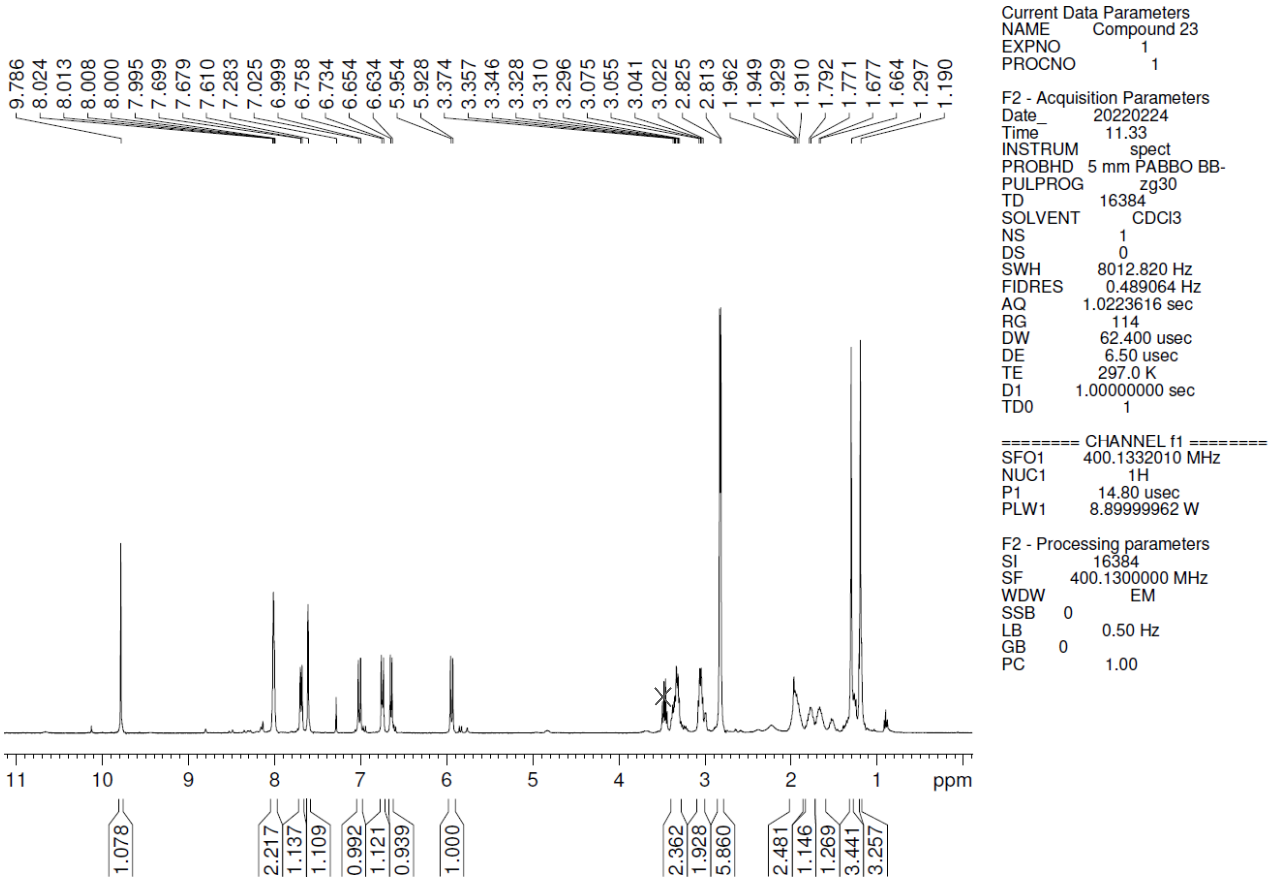

Figure S47. 1H NMR spectra of compound **23** (400 MHz, $CDCl_3$).

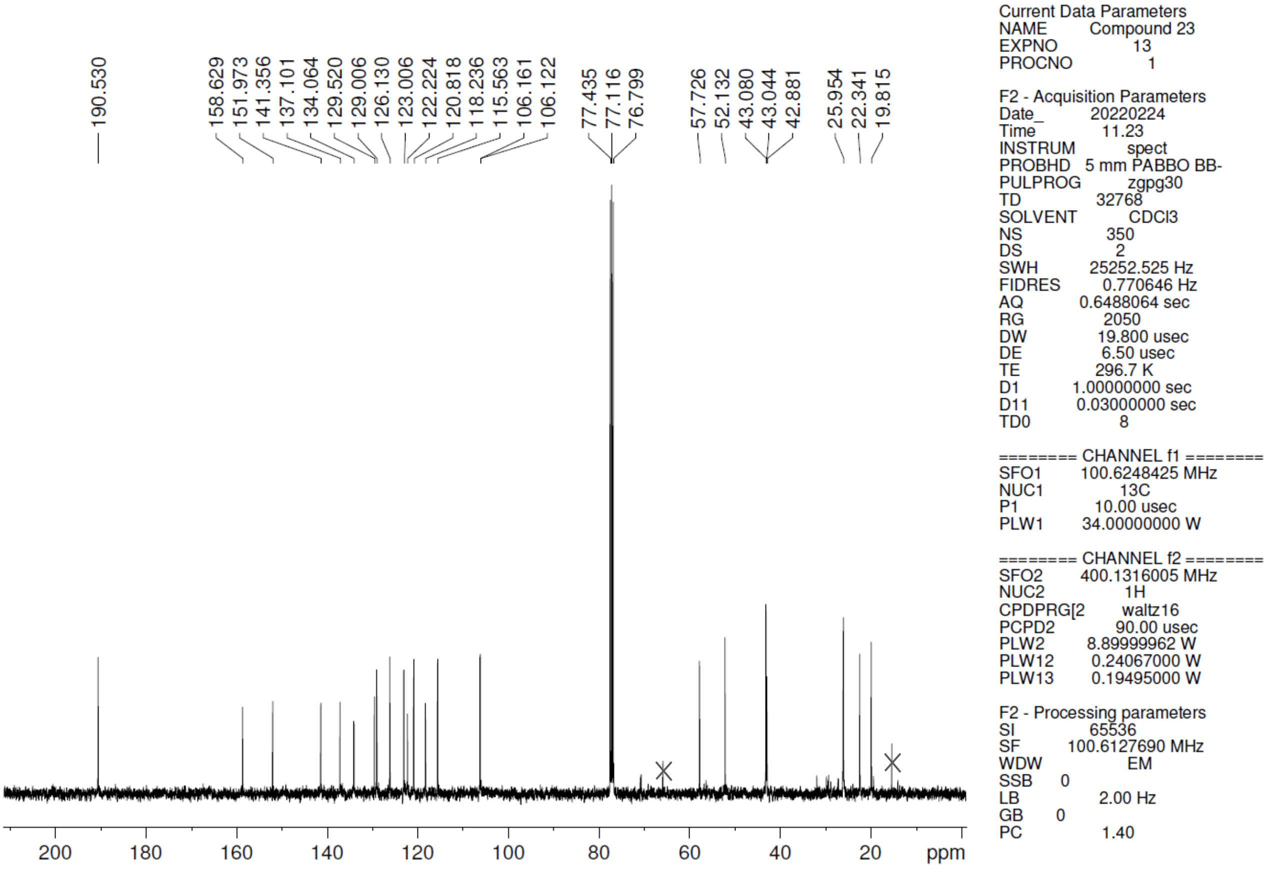

Figure S48. 13C NMR spectra of compound **23** (100 MHz, $CDCl_3$).

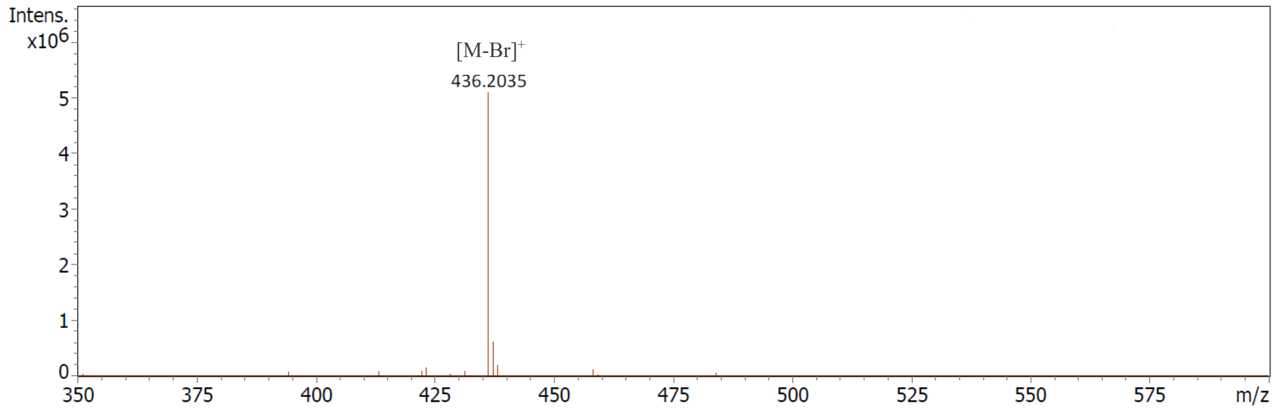

Figure S49. ESI-HRMS spectra of compound **23**.

Compound 24: ^{1}H NMR (500 MHz, $CDCl_3$): δ = 1.19 (s, 3H), 1.31 (s, 3H), 1.42 (m, 2H), 1.62 (m, 1H), 1.73 (m, 2H), 1.86 (m, *2*H), 2.81 (t, *J* = 4.3 Hz, 6H), 3.01 (m, 2H), 3.01 (m, 2H), 5.89 (d, *J* = 10.3 Hz, 1H), 6.64 (d, *J* = 8.1 Hz, 1H), 6.76 (d, *J* = 9.6 Hz, 1H), 7.03 (d, *J* = 10.4 Hz, 1H), 7.63 (d, *J* = 1.1 Hz, 1H), 7.71 (dd, *J* = 8.0, 1.5 Hz, 1H), 8.02 (d, *J* = 2.6 Hz, 1H), 8.04 (s, 1H), 9.80 (s, 1H) ppm. ^{13}C NMR (125 MHz, $CDCl_3$): δ = 19.75, 24.28, 24.33, 25.98, 28.40, 42.86, 43.02, 43.12, 52.07, 57.76, 106.08, 106.14, 115.59, 118.26, 120.75, 122.06, 122.98, 126.11, 128.95, 129.35, 134.24, 137.08, 141.33, 152.21, 158.73, 190.55 ppm.

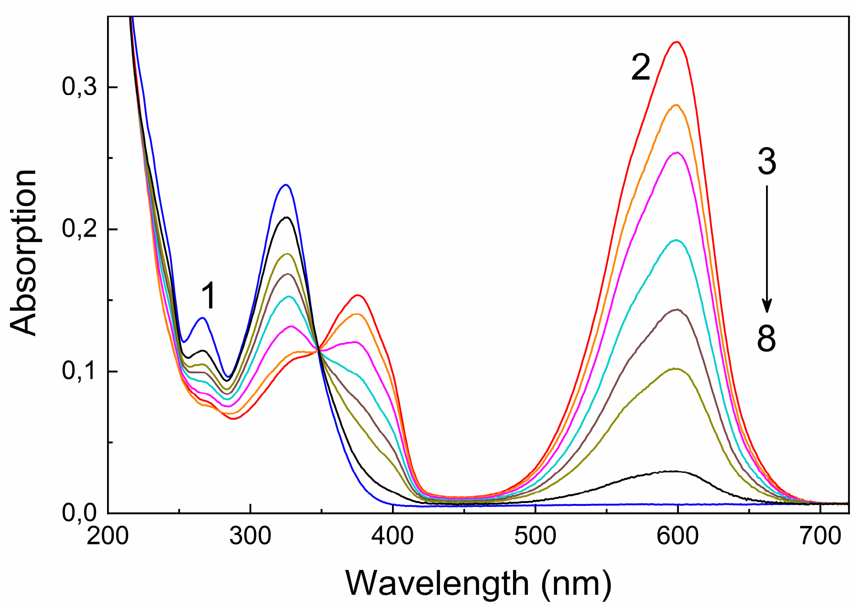

Figure S50. Absorption spectra of **23** in THF in spiropyran (1) and merocyanine forms (2-8) measured before (1) and upon UV-irradiation (2) through a UFS-1 light filter and during bleaching in the dark. (3-8). C = 10^{-4} M, L = 0.1 cm.

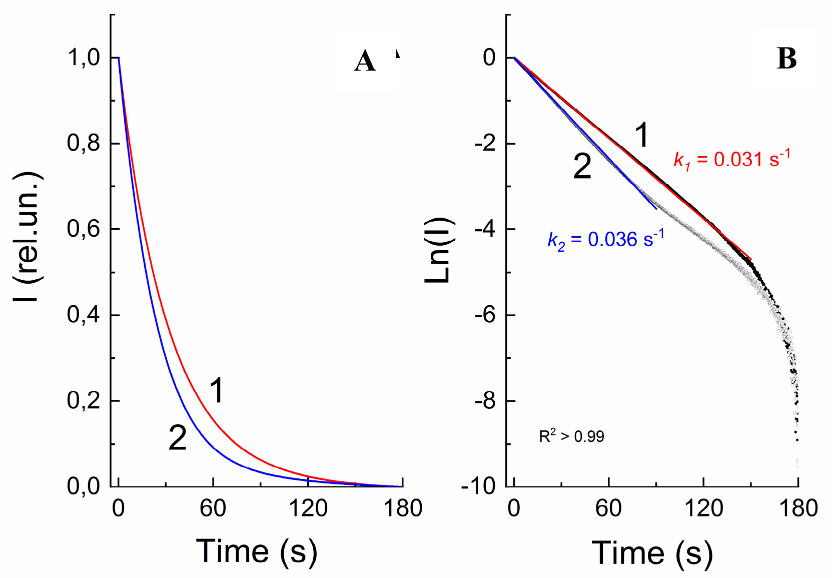

Figure S51. Normalized kinetic curves (A) of the transition process of the merocyanine form of **23** to the spiropyran form measured at 605 nm without (1) (dark bleaching) and upon irradiation (2) through a SZS-9 light filter (photobleaching). Linear anamorphoses (B) of kinetic curves 1 and 2 in the Ln(I) – time(s) coordinates.

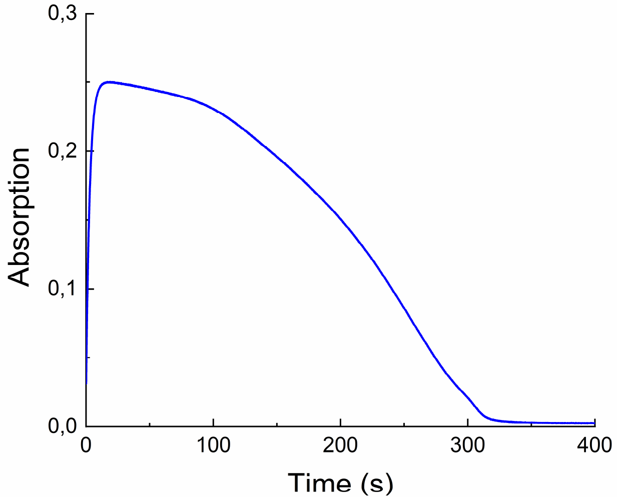

Figure S52. Kinetics of photodegradation of **23** upon UV-irradiation through a UFS-1 light filter, recorded at 605 nm.

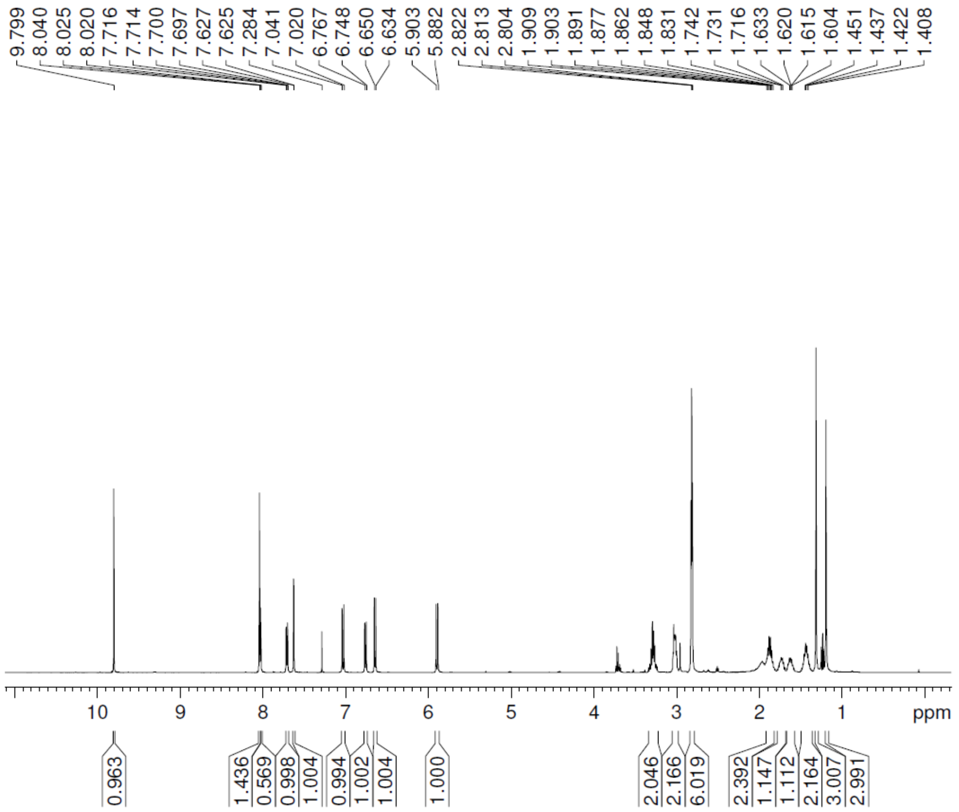

Figure S53. 1H NMR spectra of compound **24** (500 MHz, $CDCl_3$).

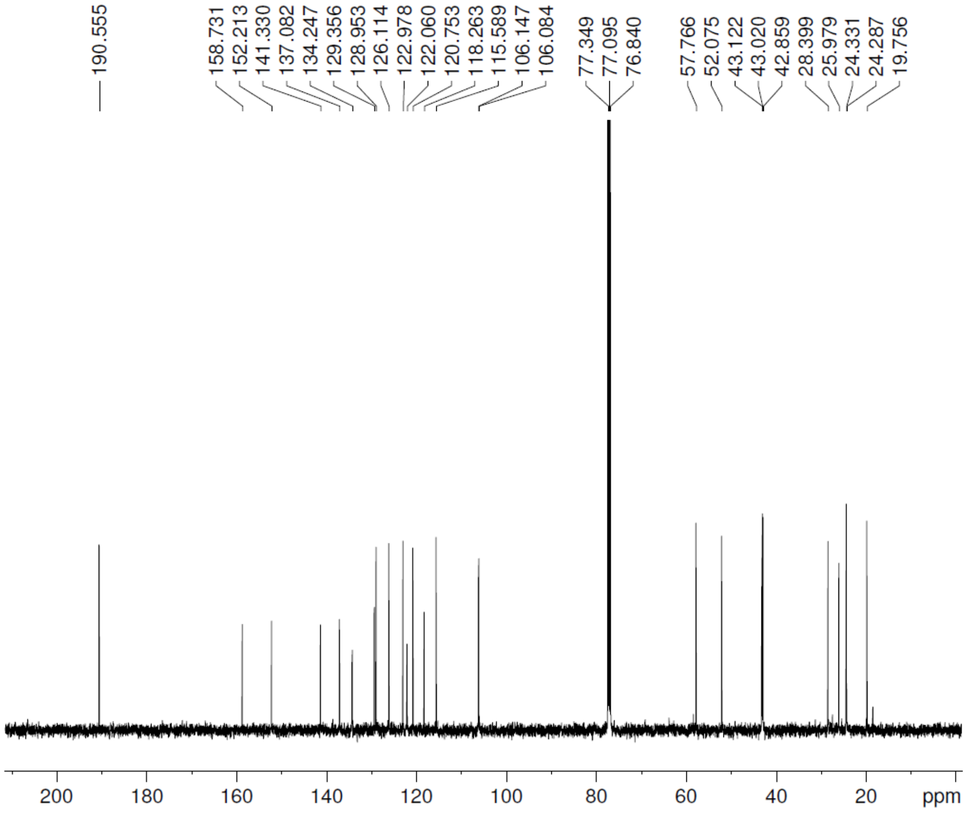

Figure S54. 13C NMR spectra of compound **24** (125 MHz, $CDCl_3$).

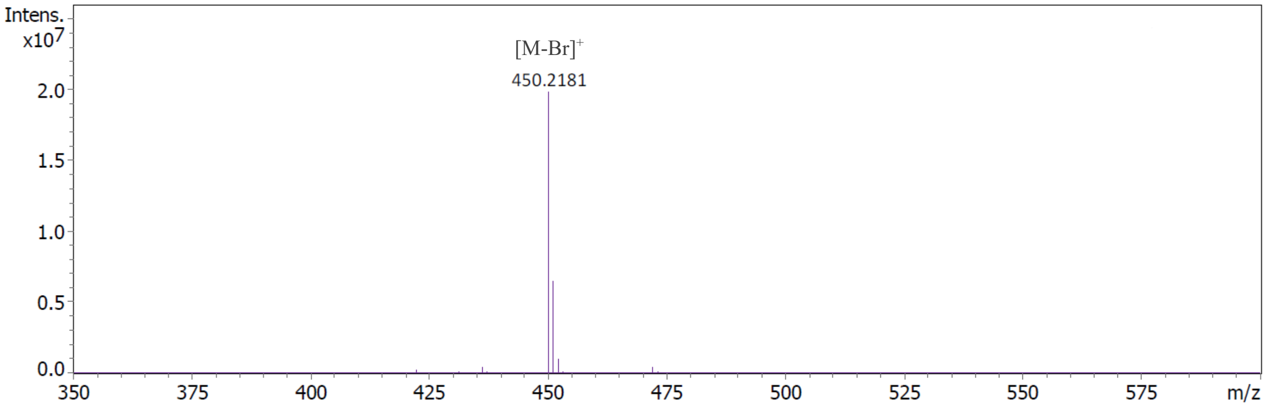

Figure S55. ESI-HRMS spectra of compound **24**.

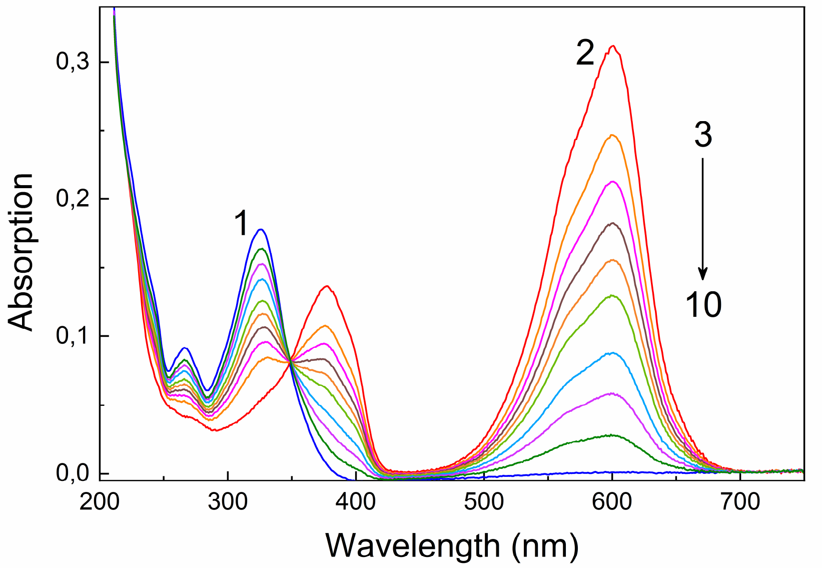

Figure S56. Absorption spectra of **24** in THF in spiropyran (1) and merocyanine forms (2-10) measured before (1) and upon UV-irradiation (2) through a UFS-1 light filter and during bleaching in the dark. (3-10). C = 10^{-4} M, L = 0.1 cm.

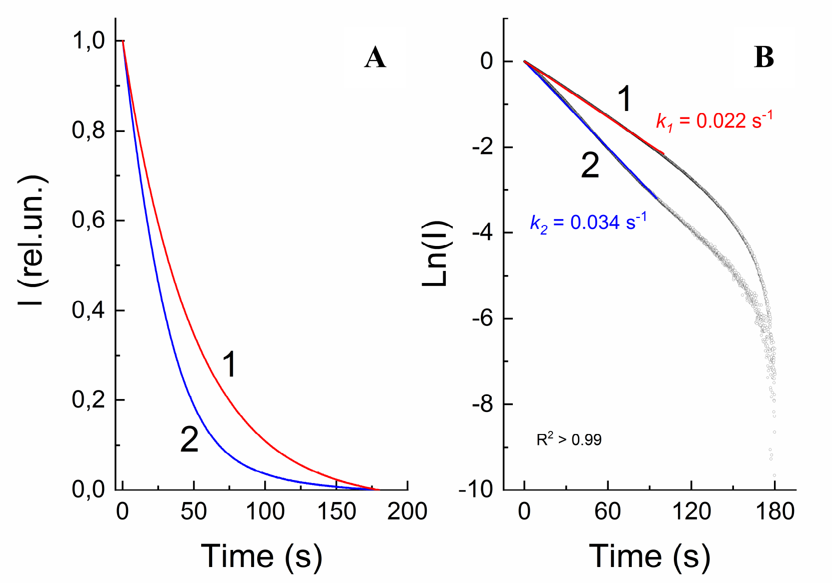

Figure S57. Normalized kinetic curves (A) of the transition process of the merocyanine form of **24** to the spiropyran form measured at 605 nm without (1) (dark bleaching) and upon irradiation (2) through a SZS-9 light filter (photobleaching). Linear anamorphoses (B) of kinetic curves 1 and 2 in the Ln(I) – time(s) coordinates.

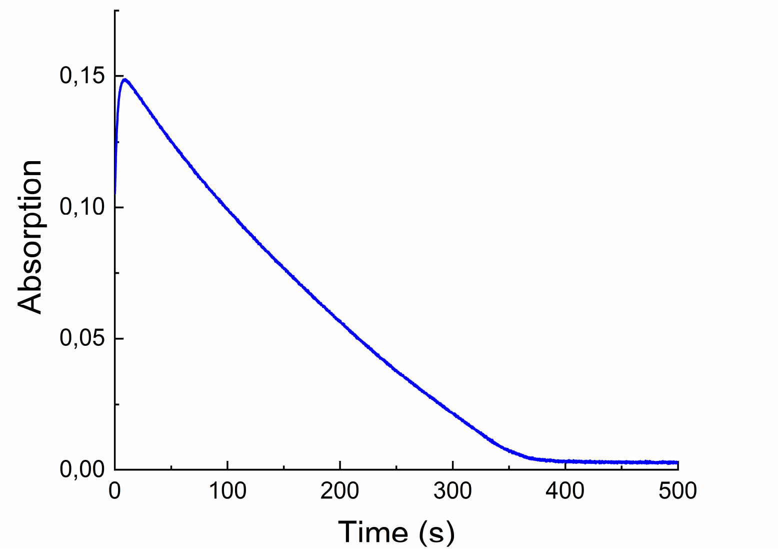

Figure S58. Kinetics of photodegradation of **24** upon UV-irradiation through a UFS-1 light filter, recorded at 605 nm.

Compound 25: ^{1}H NMR (400 MHz, $CDCl_3$): δ = 1.17 (s, 3H), 1.30 (s, 3H), 5.86 (br. d, *J* = 5.0 Hz, 3H), 1.55 (m, 1H), 1.65 (m, 1H), 1.80 (m, 2H), 1.75 (s, 6H), 2.93 (dd, *J* = 8.9, 7.2 Hz, 2H), 3.23 (m, 2H), 3.01 (m, 2H), 5.86 (d, *J* = 10.3 Hz, 1H), 6.61 (d, *J* = 8.1 Hz, 1H), 6.74 (d, *J* = 9.7 Hz, 1H), 7.00 (d, *J* = 10.4 Hz, 1H), 7.61 (s, 1H), 7.69 (d, *J* = 8.0 1H), 8.02 (d, *J* = 6.8 Hz, 2H), 9.78 (s, 1H) ppm. ^{13}C NMR (100 MHz, $CDCl_3$): δ = 19.75, 24.62, 25.99, 26.47, 26.72, 28.60, 43.09, 43.09, 43.30, 52.02, 57.97, 106.01, 106.08, 115.60, 118.24, 120.78, 122.96, 122.92, 126.07, 128.75, 129.20, 134.29, 137.03, 141.25, 152.29, 158.78, 190.52 ppm.

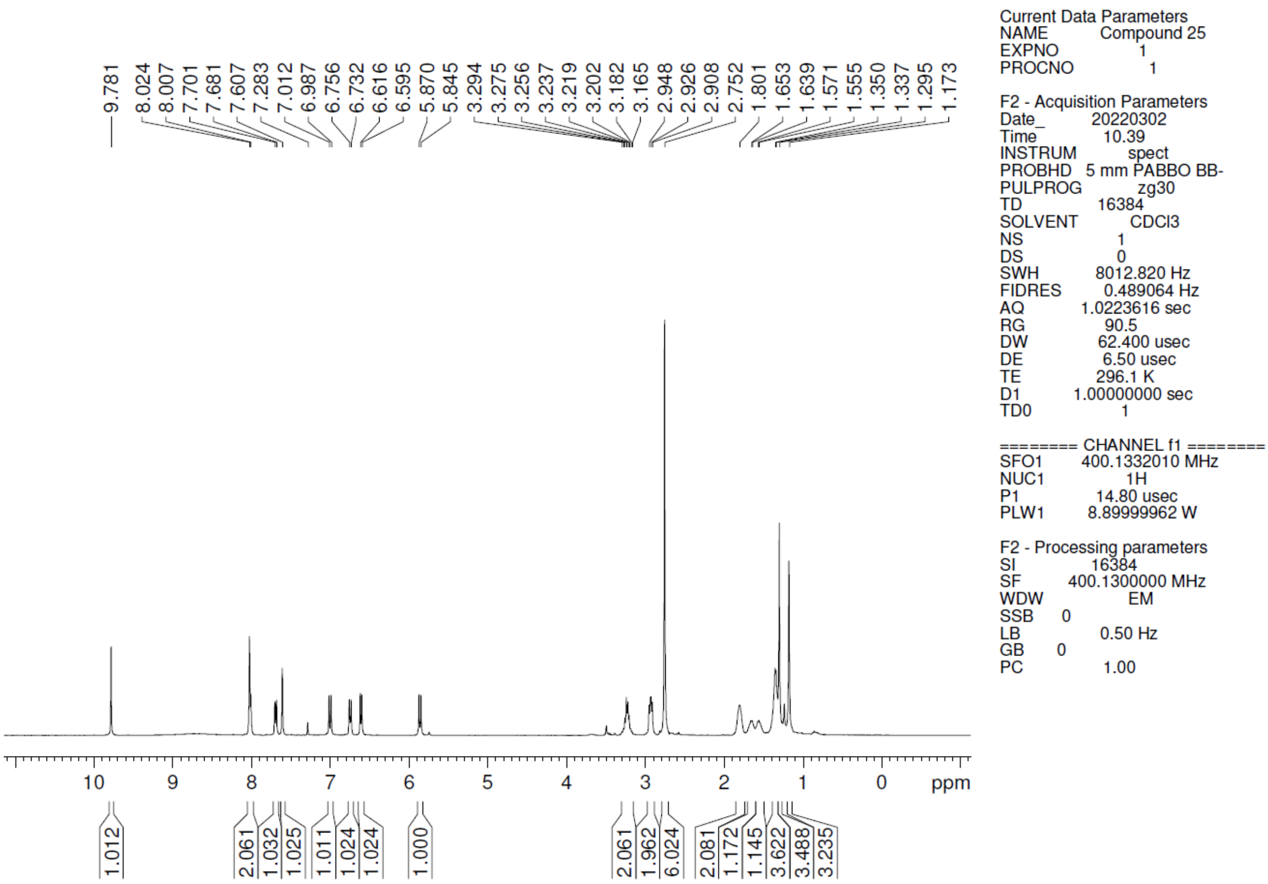

Figure S59. 1H NMR spectra of compound **25** (400 MHz, $CDCl_3$).

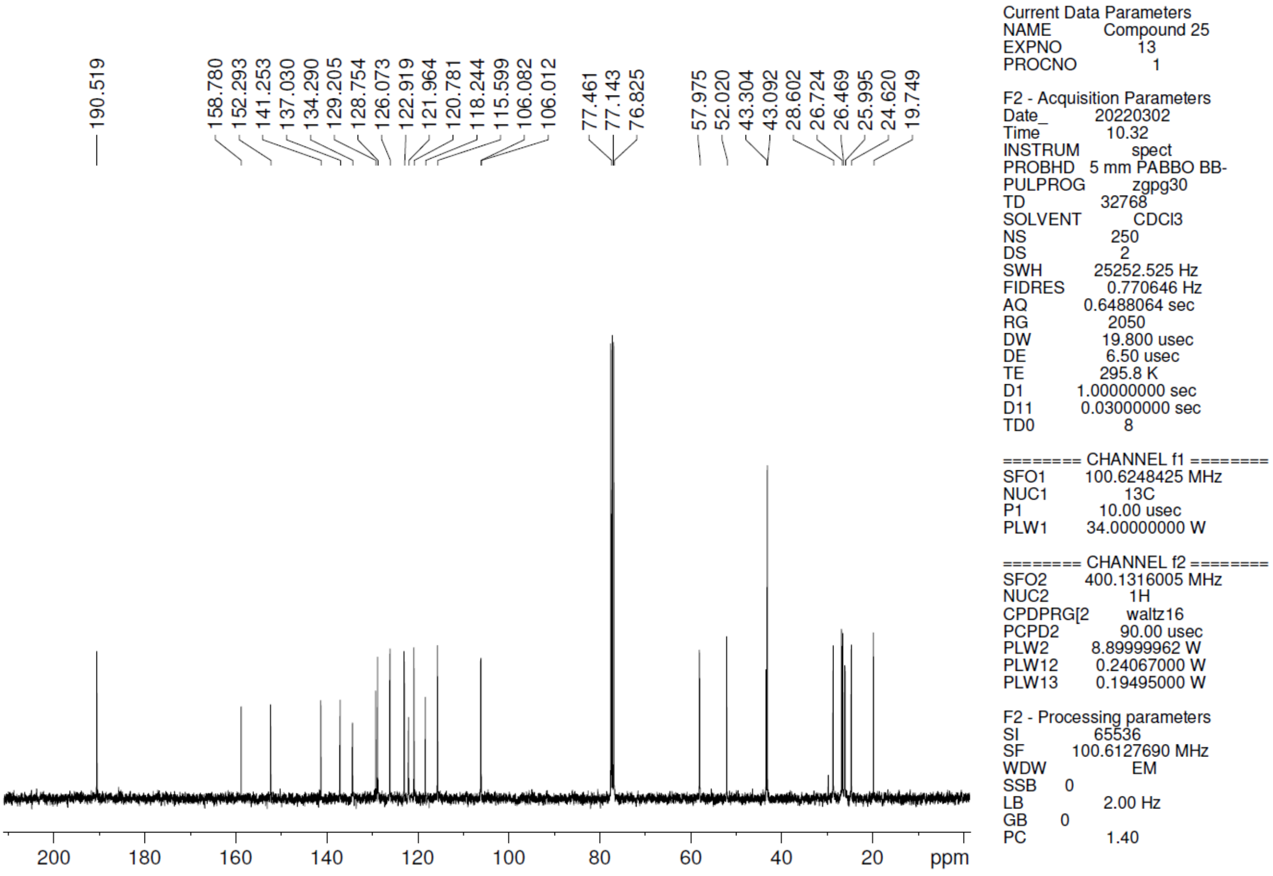

Figure S60. 13C NMR spectra of compound **25** (100 MHz, $CDCl_3$).

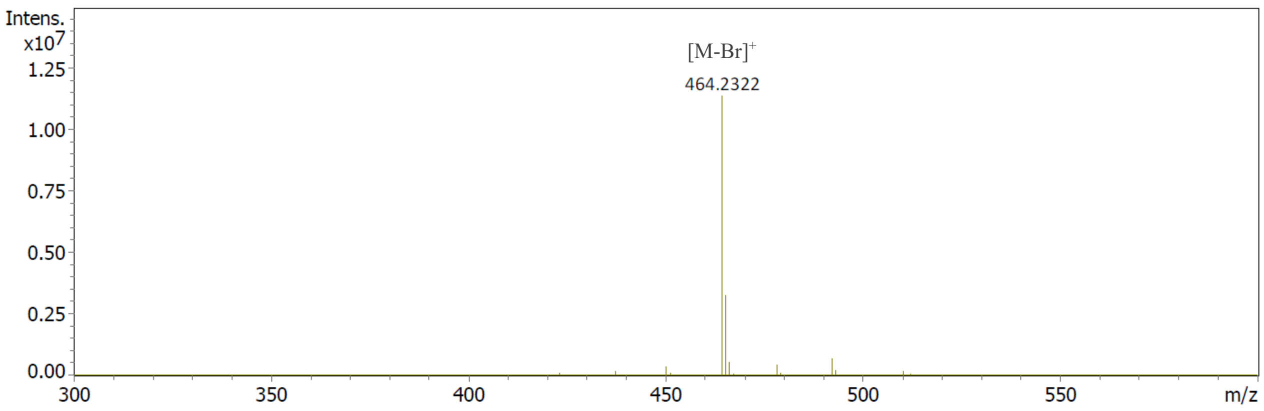

Figure S61. ESI-HRMS spectra of compound **25**.

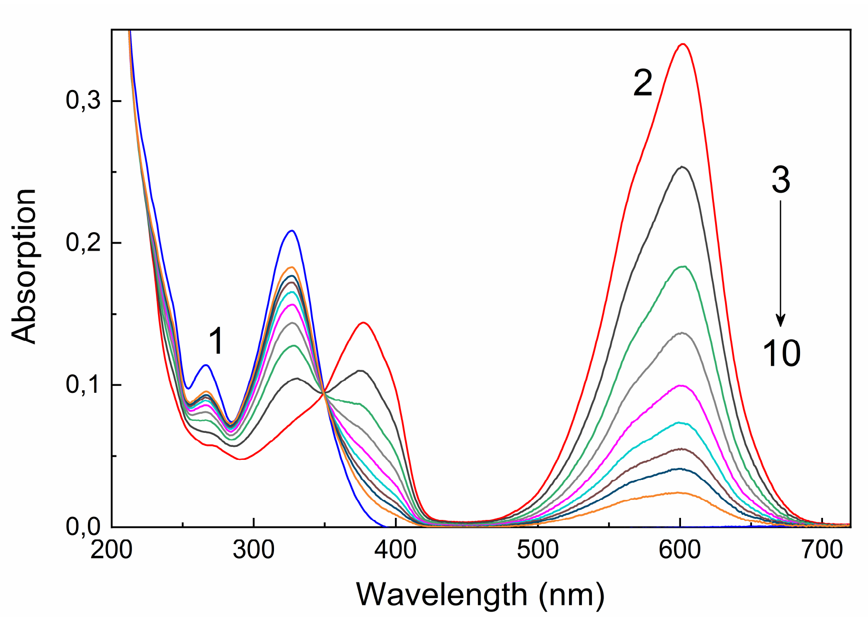

Figure S62. Absorption spectra of **25** in THF in spiropyran (1) and merocyanine forms (2-10) measured before (1) and upon UV-irradiation (2) through a UFS-1 light filter and during bleaching in the dark. (3-10). C = 10^{-4} M, L = 0.1 cm.

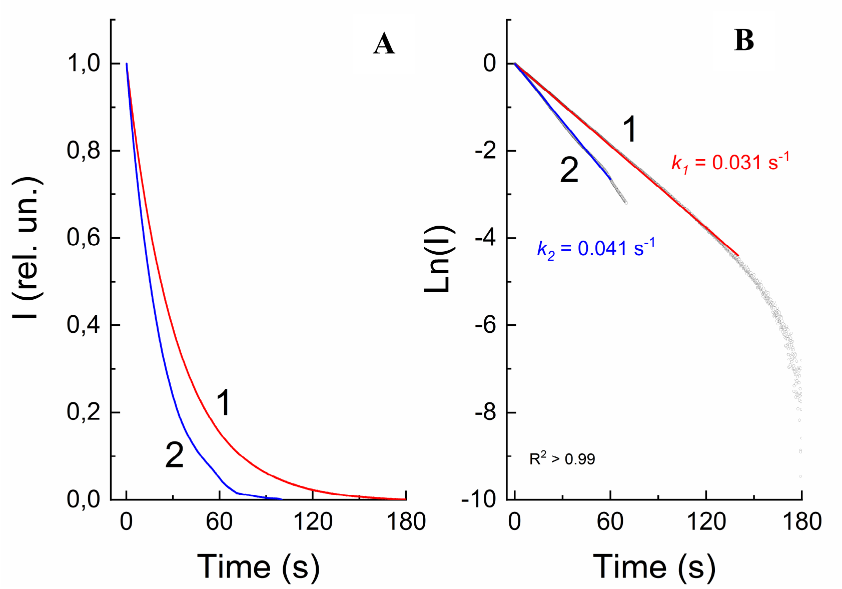

Figure S63. Normalized kinetic curves (A) of the transition process of the merocyanine form of **25** to the spiropyran form measured at 605 nm without (1) (dark bleaching) and upon irradiation (2) through a SZS-9 light filter (photobleaching). Linear anamorphoses (B) of kinetic curves 1 and 2 in the Ln(I) – time(s) coordinates.

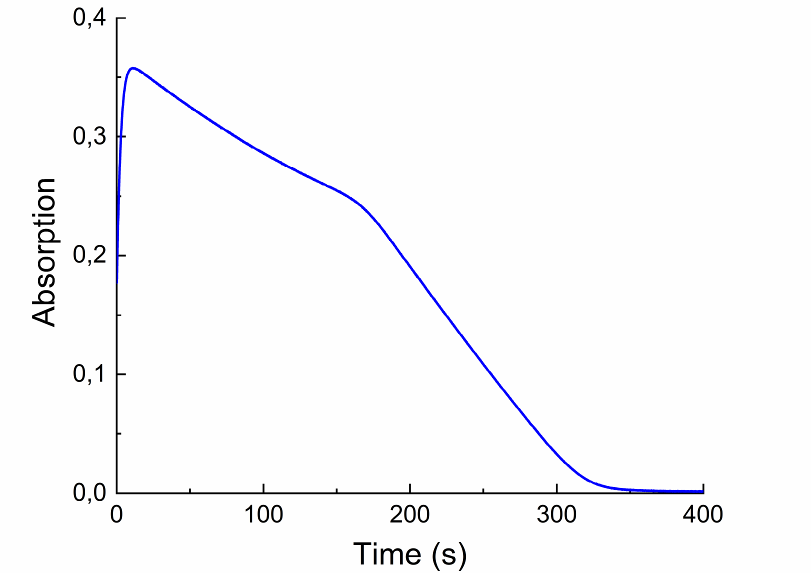

Figure S64. Kinetics of photodegradation of **25** upon UV-irradiation through a UFS-1 light filter, recorded at 605 nm.

Compound 26: 1 H NMR (400 MHz, CDCl 3): δ = 1.19 (s, 3H), 1.27 (dd, J = 8.7, 6.0 Hz, 7H), 1.32 (s, 4H), 1.52 (dd, J = 16.6, 9.9 Hz, 1H), 1.63 (m, 1H), 1.82 (m, 2H), 2.79 (s, 6H), 2.97 (m, 2H), 3.23 (m, 2H), 5.86 (d, J = 10.4 Hz, 1H), 6.61 (d, J = 8.1 Hz, 1H), 6.76 (d, J = 8.6 Hz, 1H), 7.02 (d, J = 10.3 Hz, 1H), 7.63 (s, 1H), 7.71 (d, J = 8.0, 1H), 8.03 (d, J = 2.6 Hz, 1H), 8.05 (s, 1H), 9.80 (s, 1H) ppm. 13 C NMR (100 MHz, CDCl 3): δ = 19.75, 24.40, 26.04, 26.57, 27.01, 28.64, 28.89, 28.97, 42.99, 43.40, 51.98, 58.10, 105.97, 106.02, 115.62, 118.28, 120.77, 121.94, 122.90, 126.04, 128.67, 129.16, 134.30, 137.05, 141.26, 152.33, 158.82, 190.50 ppm.

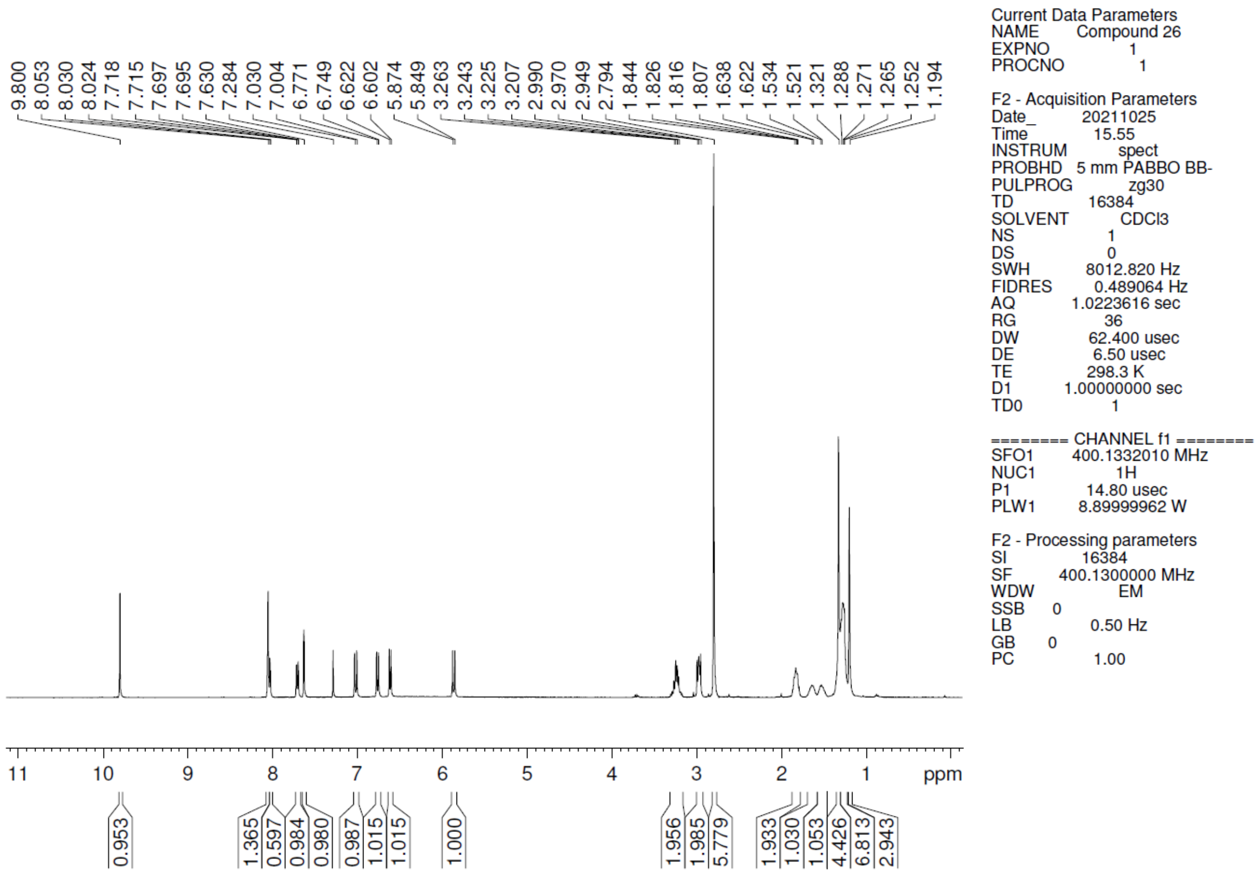

Figure S65. 1H NMR spectra of compound **26** (400 MHz, $CDCl_3$).

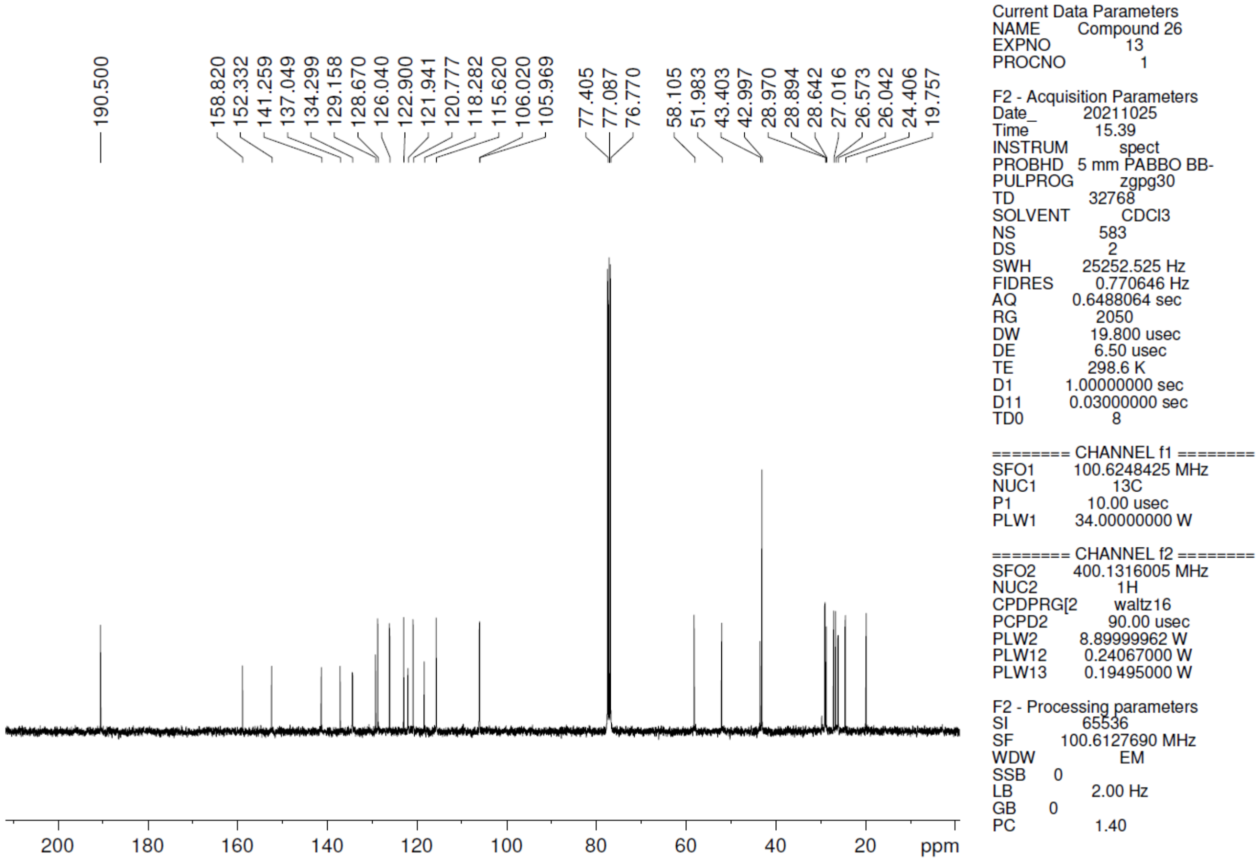

Figure S66. 13C NMR spectra of compound **26** (100 MHz, $CDCl_3$).

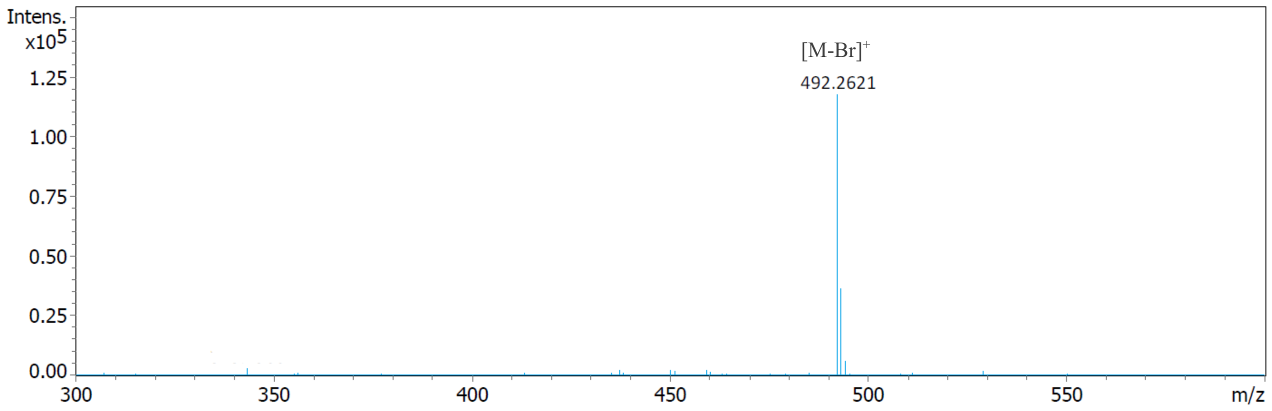

Figure S67. ESI-HRMS spectra of compound **26**.

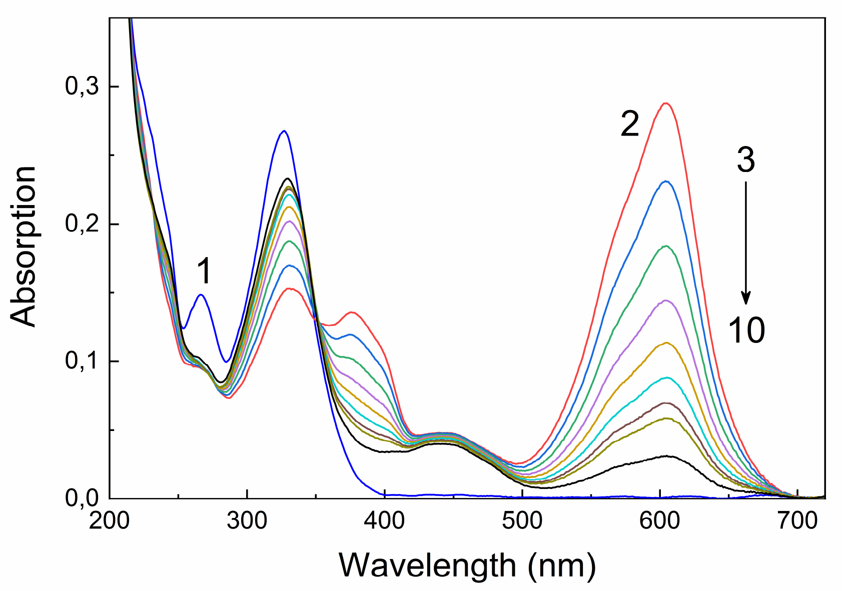

Figure S68. Absorption spectra of **26** in THF in spiropyran (1) and merocyanine forms (2-10) measured before (1) and upon UV-irradiation (2) through a UFS-1 light filter and during bleaching in the dark. (3-10). C = 10^{-4} M, L = 0.1 cm.

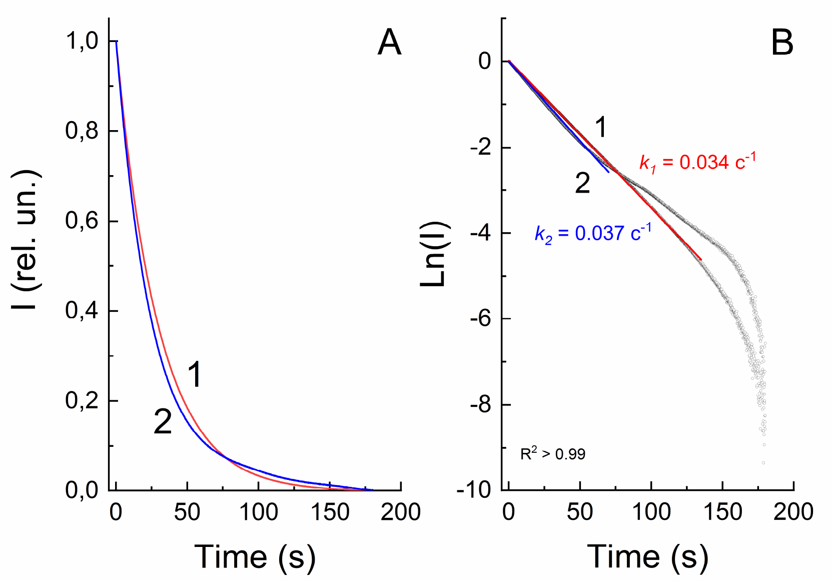

Figure S69. Normalized kinetic curves (A) of the transition process of the merocyanine form of **26** to the spiropyran form measured at 605 nm without (1) (dark bleaching) and upon irradiation (2) through a SZS-9 light filter (photobleaching). Linear anamorphoses (B) of kinetic curves 1 and 2 in the Ln(I) – time(s) coordinates.

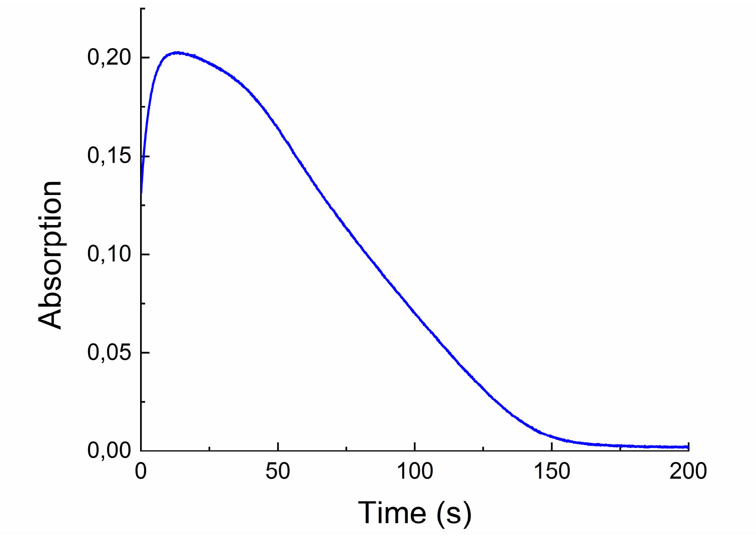

Figure S70. Kinetics of photodegradation of **26** upon UV-irradiation through a UFS-1 light filter, recorded at 605 nm.
